# Protein-centric omics integration analysis identifies candidate plasma proteins for multiple autoimmune diseases

**DOI:** 10.1007/s00439-023-02627-0

**Published:** 2023-12-24

**Authors:** Yingxuan Chen, Shuai Liu, Weiming Gong, Ping Guo, Fuzhong Xue, Xiang Zhou, Shukang Wang, Zhongshang Yuan

**Affiliations:** 1 Department of Biostatistics, School of Public Health, Cheeloo College of Medicine, Shandong University, 44, Wenhua West Road, Jinan, 250012 Shandong China; 2 Institute for Medical Dataology, Shandong University, 12550, Erhuan East Road, Jinan, 250003 Shandong China; 3 Department of Biostatistics, University of Michigan, Ann Arbor, MI 48109 USA; 4 Center for Statistical Genetics, University of Michigan, Ann Arbor, MI 48109 USA

**Keywords:** Autoimmune disease, Plasma protein, Proteome-wide association study, Mendelian randomization, Colocalization, Therapeutic target

## Abstract

It remains challenging to translate the findings from genome-wide association studies (GWAS) of autoimmune diseases (AIDs) into interventional targets, presumably due to the lack of knowledge on how the GWAS risk variants contribute to AIDs. In addition, current immunomodulatory drugs for AIDs are broad in action rather than disease-specific. We performed a comprehensive protein-centric omics integration analysis to identify AIDs-associated plasma proteins through integrating protein quantitative trait loci datasets of plasma protein (1348 proteins and 7213 individuals) and totally ten large-scale GWAS summary statistics of AIDs under a cutting-edge systematic analytic framework. Specifically, we initially screened out the protein-AID associations using proteome-wide association study (PWAS), followed by enrichment analysis to reveal the underlying biological processes and pathways. Then, we performed both Mendelian randomization (MR) and colocalization analyses to further identify protein-AID pairs with putatively causal relationships. We finally prioritized the potential drug targets for AIDs. A total of 174 protein-AID associations were identified by PWAS. AIDs-associated plasma proteins were significantly enriched in immune-related biological process and pathways, such as inflammatory response (*P* = 3.96 × 10^–10^). MR analysis further identified 97 protein-AID pairs with potential causal relationships, among which 21 pairs were highly supported by colocalization analysis (PP.H4 > 0.75), 10 of 21 were the newly discovered pairs and not reported in previous GWAS analyses. Further explorations showed that four proteins (*TLR3*, *FCGR2A*, *IL23R*, *TCN1*) have corresponding drugs, and 17 proteins have druggability. These findings will help us to further understand the biological mechanism of AIDs and highlight the potential of these proteins to develop as therapeutic targets for AIDs.

**Supplementary Information:**

The online version contains supplementary material available at 10.1007/s00439-023-02627-0.

## Introduction

Autoimmune diseases (AIDs) are a group of diseases characterized with the immune system being misdirected to attack the host itself and cause damage to its own tissues (Rosenblum et al. 2015; Wang et al. 2015). AIDs have become one of the leading causes of death, especially in young and middle-aged women (Cooper and Stroehla 2003; Rogers et al. 2020; Walsh and Rau 2000). Genome-wide association studies (GWAS) have identified hundreds of thousands of genetic variants that are associated with AIDs (Buniello et al. 2019; Kochi 2016; Lettre and Rioux 2008). However, it is still in the infancy to clinically translate GWAS findings into intervention targets, which may be presumably due to the lack of knowledge on how the GWAS risk variants contribute to AIDs (Miller 2023; Orozco 2022). So far, the treatment for AIDs is either to alleviate the consequences caused by the pathological changes and tissue damage, or to block the disease process by modulating the immune response (Chandrashekara 2012). In addition, current immunomodulatory drugs for AIDs are broad in action rather than disease-specific, it is desirable to further elucidate the functional interpretation of GWAS risk variants, perhaps through comprehensive omics integration analysis, so as to expectedly provide some clues for the potential disease-specific intervention targets.

Human immune processes are closely related to proteins, which are the product of translated DNA and functional elements that could bridge the genetic variants codes and disease. The plasma proteins originate from various organ tissues and can play significant roles in the development of AIDs (Brzezicka and Paulson 2022; Gupta and Hawkins 2015; Virtanen et al. 2019; Yin et al. 2018). More importantly, plasma proteins are well known as effective therapeutic targets, with most approved drugs targeting specific proteins (Fugger et al. 2020). On the other hand, the recent proliferation of publicly available GWAS of AIDs as well as the protein quantitative trait loci (pQTL) studies provide a rich resource of large-scale summary data without the privacy and ethical issue and promote the omics-integration analysis to combine GWAS with pQTL studies, which are expected to provide novel insights into the pathophysiology of AIDs and to benefit the targeted therapy (Jiang et al. 2020; Patterson et al. 2014).

Several statistical genetics methods have been developed to integrate GWAS and pQTL summary statistics, aiming to identify potential disease-related proteins. In particular, proteome-wide association study (PWAS) is able to detect the protein-coding genes associated with phenotypes through protein function alterations (Wingo et al. 2021a, 2021c). Mendelian randomization (MR) analysis can evaluate the causal effect of an exposure (e.g., protein) on an outcome of interest (e.g., AID) via instrumental variables (IVs). Since genetic variants were randomly allocated from parents to offspring at conception and would not be modified, MR can be thought of a “naturally” randomized controlled trials (Haycock et al. 2016) and is well acknowledged to be an efficient and cost-effective method to investigate the causal relationships among molecular traits and disease (Liu et al. 2021; Yuan et al. 2020). In addition, colocalization analysis is able to examine and identify the shared causal variants between proteins and diseases. Some previous studies have applied MR analysis and colocalization to identify the potentially causal plasma proteins for AIDs, such as MS (A. Staley 2020), hypothyroidism (Yang et al. 2023), inflammatory bowel disease (IBD) (Chen et al. 2023; Mi et al. 2022), and type 1 diabetes (T1D) (Yazdanpanah et al. 2022). However, these studies mainly rely on MR analysis and lack comprehensive analysis. Only conducting MR analysis will be vulnerable to model misspecification of a univariate method, insufficient to systematically evaluate the findings. Indeed, different analysis techniques, though with different focus, could complement each other. For example, PWAS analysis could be readily adopted prior to MR causal analysis to initially screen out the plausible protein-disease associations, while colocalization analysis can be used to examine the bias from MR analysis due to the linkage disequilibrium (LD) (Zuber et al. 2022). Thus, joint analysis using different methods can provide a better understanding of the relationship between proteins and AIDs.

In this study, we aimed to integrate the publicly available large pQTL datasets of plasma proteins and ten large-scale GWAS summary statistics of AIDs, including ankylosing spondylitis (AS), celiac disease (CD), hypothyroidism, IBD, multiple sclerosis (MS), myasthenia gravis (MG), pernicious anemia (PA), rheumatoid arthritis (RA), systemic lupus erythematosus (SLE) and T1D, to identify disease-associated plasma proteins under a cutting-edge analytic framework by sequentially using PWAS, MR, and colocalization. Specifically, we first performed PWAS analysis to initially identify the protein-disease associations, followed by enrichment analysis and protein–protein interaction (PPI) network analysis to explore the underlying biological processes and pathways. Targeting on PWAS significant proteins, we then used two-sample MR analysis (Hemani et al. 2018) parallelized with colocalization analysis to screen out potentially causal proteins. We finally investigated the protein function for multiple AIDs and explored the potential drug targets using the Drug-Gene Interaction Database (DGIdb).

## Materials and methods

### Study design

**Fig. 1:**
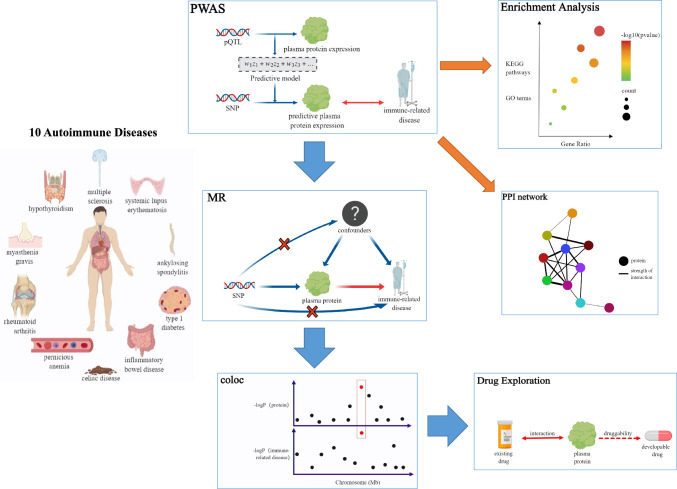
Study design. *PWAS* proteome-wide association study, *pQTL* protein quantitative trait loci, *SNP* single nucleotide polymorphism, *MR* Mendelian Randomization, *KEGG* Kyoto Encyclopedia of Genes and Genomes, *GO* Gene Ontology, *PPI* protein–protein interaction

The study design overview is presented in Fig. 1. By combining protein quantitative trait loci datasets of plasma protein and a total of 10 large-scale GWAS summary statistics of AIDs, we performed comprehensively protein-centric omics integration analysis through sequentially using PWAS, MR and colocalization analyses to identify the plasma proteins that are associated with AIDs. All these analyses, paired with enrichment analysis and drug exploration analysis, further help investigate the homogeneity and heterogeneity across multiple AIDs as well as to prioritize the potential drug targets.

### Data source

#### GWAS summary data

We collected publicly available GWAS summary statistics with European ancestry for AIDs. To ensure statistical power, we screened GWAS with sample sizes larger than 10,000 and the number of cases larger than 1000 and finally included 10 GWAS summary statistics. Specifically, nine GWASs were obtained from the GWAS catalog, including AS (1344 cases and 324,074 controls), CD (2364 cases and 324,074 controls), hypothyroidism (23,497 cases and 461,101 controls), MS (1683 cases and 324,074 controls), MG (1873 cases and 36,370 controls), PA (1555 cases and 324,074 controls), RA (6360 cases and 324,074 controls), SLE (5201 cases and 9066 controls), and T1D (18,942 cases and 501,638 controls). GWAS summary statistics for IBD were obtained from the UK Biobank (Wu et al. 2021). All GWASs were approved by relevant ethics committees, with more details provided in Table S1.

#### Human plasma pQTL data and imputation model

The pQTL studies aim to investigate the association between genetic variants and protein expression levels, which have recently been used to combine with GWASs to illuminate the underlying mechanisms of complex diseases. Here, we used the plasma pQTL data from 7213 European Americans in the Atherosclerosis Risk in Communities (Zhang et al. 2022a), including 4657 plasma proteins measured by Slow Off-Rate Modified Aptamers (SOMAmers) assay on the SomaLogic version-4 platform, where plasma proteins were first adjusted for covariates in a linear regression and then associated the rank-inverse normalized residuals with genetic variants. The detailed quality control and analysis procedure were described in the original publications. In PWAS analysis, we mainly focused on the 1348 significant *cis*-heritable plasma proteins (i.e. the nonzero cis-heritability with *P* < 0.01) with available imputation weights, which were derived using the Elastic Net algorithm.

### Statistical analysis

#### Proteome-wide association studies

PWAS aims to explore the association between protein and disease by integrating the genetic imputation model of protein expression with GWASs. We conducted PWAS analysis using the FUSION pipeline with the available imputation weights of 1348 significant cis-heritable plasma proteins (Gusev et al. 2016). Once inputting the GWAS summary data and SNP-protein imputation weights, FUSION typically predicts each protein's abundance in the GWAS and then perform an association analysis between the predicted protein abundance and AIDs. We used the 1000 Genomes European panel as LD reference data. Here, we mainly focus on the association between plasma proteins and AIDs, thus the multiple tests for the protein-AID pairs are often non-independent. In such case, the traditional Bonferroni correction for these multiple correlated tests is often too stringent. We, therefore, adopted the False Discovery Rate (FDR) (Benjamini-Hochberg method) corrections for the multiple testing and declared the significant proteins with FDR < 0.05, which have been commonly used in the previous literature (Bouras et al. 2022; Gong et al. 2023; Zhang et al. 2021). Furthermore, to verify whether the identified targets are unique to PWAS, we performed Transcriptome-Wide Association Studies (TWAS) analysis in whole blood and further examined whether the PWAS signals could be explained by cis-genetic regulation of the expression of nearby (1 Mb region around) genes by performing conditional analysis(Zhang et al. 2022b). Specifically, we performed TWAS analysis using FUSION with the SNP effect size on gene expression obtained from Elastic-net models. Then, for each significant PWAS loci, we searched all TWAS genes nearby (± 500 kb around) whose transcription start site (TSS) locate within 500 kb of the TSS of its sentinel PWAS gene, and selected the one with the smallest TWAS *P* value for conditional analysis. That is, to examine whether the PWAS signals still remained conditioning on imputed expression values of the gene with the smallest TWAS *P* value. In addition, for genes encoded the PWAS significant proteins, we also searched them in TWAS and performed conditional analysis to determine whether these PWAS signals still remained given the same genes in TWAS analysis.

#### Enrichment analysis and PPI network

To explore the possible biological mechanisms involved in AID-associated proteins identified by PWAS, we performed the enrichment analysis using Metascape. Metascape computes pairwise similarities between any two enriched terms based on a Kappa test score, automatically clusters enriched terms into non-redundant groups. *P* values were produced using a hypergeometric test and corrected by the Benjamini–Hochberg FDR method (Zhou et al. 2019). The parameters of Min Overlap, *P* Value Cutoff, and Min Enrichment are set as the default values. Here, we selected Gene Ontology (GO) and Kyoto Encyclopedia of Genes and Genomes (KEGG) databases to carry out enrichment analysis, respectively. In addition, we used the STRING database to infer enriched protein clusters and generate the PPI network to explore the interrelationships among the significant proteins identified by PWAS (Szklarczyk et al. 2019).

#### Mendelian randomization analysis

For the significant protein-AID pairs from PWAS analysis, we further performed two-sample MR analysis, together with a series of sensitivity analyses, to assess the potential causal effect of protein on AIDs using the R package TwoSampleMR. The MR analysis conforms to the STROBE-MR Statement (Skrivankova et al. 2021), mainly involving instrumental variable selection, instrumental variable assessment, primary MR analysis as well as sensitivity analysis.

We first selected instrumental variables for plasma proteins from its cis-pQTLs. Taking the different number of cis-SNPs within the cis-region of different proteins into account, we adopted a protein-specific Bonferroni-corrected *P*-value threshold (0.05/the number of SNPs in the cis-region) to declare significant pQTLs and obtained the protein-specific independent cis-pQTLs. Specifically, we selected the protein-specific independent cis-pQTLs by performing linkage-disequilibrium (LD) clumping, with the threshold of *r*^2^ < 0.01 in the 1 Mb cis-region. LD calculation was based on the European LD reference panel in the 1000 Genomes. After harmonizing the effect alleles of IVs in pQTLs data and that in outcome GWAS data, the retained SNPs were used for MR analysis. It should be noted that the appropriateness of IVs is a prerequisite for MR analysis. Thus, we assessed the strength of IVs by the *F* statistic and removed the weak IVs with the F-statistic less than 10 (Bottigliengo et al. 2022; Burgess et al. 2011; Palmer et al. 2012; Wang et al. 2021). To further remove potential pleiotropic genetic variants, we used the Phenoscanner (Kamat et al. 2019), to identify SNPs associated with the AIDs and removed them from the analysis. In addition, to infer the causal effect of proteins on AIDs risk, we expected IVs to affect protein expression first and then the AIDs risk. Therefore, we further conducted the MR Steiger directionality test to assess whether the MR analysis was biased by reverse causation. The MR analysis is unlikely to be substantially influenced by the reverse causation with *P* value from the MR steiger directionality test less than 0.05.

For primary MR analysis, we used the Wald Ratio method for proteins with only one IV, the fixed-effect inverse-variance weighted (IVW) method (Bowden et al. 2017) for proteins with two or three IVs, and the random-effect IVW method for proteins with four or more IVs. Of note, the random effects model is able to account for heterogeneity across IVs by allowing for over-dispersion of the regression model. All causal estimates of plasma proteins on AIDs are reported to be odds ratio, indicating the change of AIDs risk per one SD change in protein abundance. To further strength the validity of the MR results, we performed MR-Egger and weighted Median MR analyses. Briefly, MR-Egger can allow for the detection and correction of horizontal pleiotropy, where the intercept can be used to identify the existence of horizontal pleiotropy (Burgess and Thompson 2017). While the weighted median method can provide a consistent estimator even when up to 50% of the information comes from invalid genetic instruments (GIs), and is robust to some degree of heterogeneity among GIs (Bowden et al. 2016). On top of IVW results, we further assessed the issue of heterogeneity and horizontal pleiotropy, and integrated the results of MR-Egger and Weighted median to determine the final MR results. Again, we used FDR (Benjamini-Hochberg method) to perform multiple testing corrections in MR analysis.

For sensitivity analysis, we first fitted the MR-Egger model (Bowden et al. 2015) and considered a significant intercept term (*P* < 0.05) as an indicator of horizontal pleiotropy. Then, we calculated Cochran’s *Q* statistic to assess heterogeneity for proteins with more than one IV. We finally applied the leave-one-out approach to test whether the MR estimates were dominantly driven by one IV and removed those results when removing one SNP yielded an IVW estimate that differed from the overall IVW estimate.

#### Bayesian colocalization analysis

For the potential causal protein-AID pairs identified by MR analysis, we further performed the colocalization analysis using the R package coloc with default parameter setting (Wallace 2021). Bayesian colocalization was able to assess the probability that AIDs risk loci and proteins share the same variant, rather than the variant shared coincidentally due to LD correlation (Giambartolomei et al. 2014), which would help to examine the bias in MR analysis due to LD. Typically, the colocalization provides five assumptions: H0, no association with either AID or protein (PP0); H1, association with AID, not with protein (PP1); H2, association with protein, not with AID (PP2); H3, association with AID and protein, two independent SNPs (PP3); and H4, association with AID and protein, only one shared SNP (PP4) (Giambartolomei et al. 2014). We mainly focus on H4 and considered a strong evidence of colocalization when PP.H4 is larger than 0.75.

#### Druggable targets exploration

To explore if the proteins identified above can serve as targets of the existing drugs or druggable gene targets, we explored the interactions between these proteins (or genes) and drugs using Drug-Gene Interaction Database (DGIdb) (version 4.0) (https://www.dgidb.org/), DGIdb provides search and filtering of drug-gene interactions and drug genomic information. The database integrates more than 30 trusted sources, such as DrugBank, pharmkb, Chembl, Drug Target Commons, Therapeutic Target Database (TTD), etc., containing more than 40,000 genes and 10,000 drugs, involving more than 100,000 drug-gene interactions or belongs to one of 42 potential drug-gene classes, which has been widely used to prioritize the potential drug targets for diseases (Freshour et al. 2021; Griffith et al. 2013). Using DGIdb, we can not only search the established interactions between genes and drugs but also explore whether the genes are ‘potentially’ druggable according to their membership in gene categories associated with druggability (e.g., kinases).

## Results

### PWAS identified 174 protein-AID pairs

**Fig. 2:**
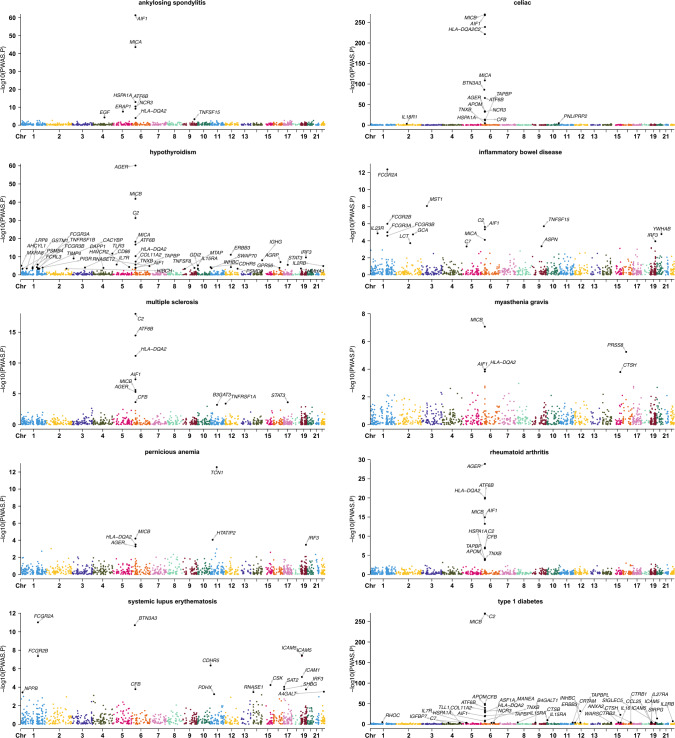
Manhattan plots of PWAS analysis for 10 autoimmune diseases. Significant proteins (FDR-corrected *P* < 0.05) are marked with coding genes. *IGHG* IGHG1|IGHG2|IGHG3|IGHG4|IGK@|IGL@

**Fig. 3:**
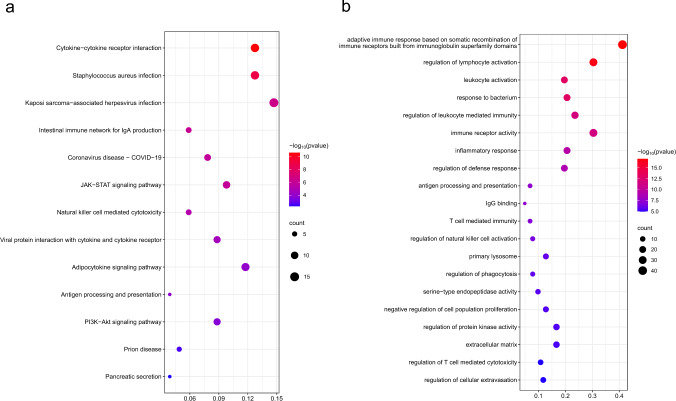
Bubble chart for enrichment analysis of PWAS results. **a** Bubble chart for KEGG enrichment analysis; **b** Bubble chart for GO enrichment analysis

By integrating GWASs of ten AIDs with the imputation models of 1348 cis-heritable proteins from the pQTL data, we identified a total of 174 significant protein-AID pairs with FDR adjusted *P*-value less than 0.05 (Fig. 2), including 9 for AS, 16 for CD, 45 for hypothyroidism, 16 for IBD, 10 for MS, 5 for MG, 6 for PA, 11 for RA, 16 for SLE, and 40 for T1D, with details provided in Table S2. Among the 174 PWAS significant signals, a total of 143 PWAS signals still remained through the conditional analysis and only 31 PWAS signals can be explained by TWAS analysis (Table S3). We also searched the genes that encoded the PWAS significant proteins in TWAS and finally found 70 TWAS genes. The results illustrated that, among the 70 TWAS genes, there are 62 PWAS signals remained through the conditional analysis and only 8 PWAS signals can be explained by TWAS genes (Table S4). All these findings indicated that the identified targets are unique to PWAS and not simply genes in close proximity or results from TWAS. We further performed enrichment analysis on these proteins and mapped the PPI network. For GO enrichment analysis, we identified 20 significant GO terms (Fig. 3, Table S5), such as adaptive immune response based on somatic recombination of immune receptors built from immunoglobulin superfamily domains (*P* = 9.24 × 10^–18^), regulation of leukocyte mediated immunity (*P* = 1.94 × 10^–12^), and inflammatory response (*P* = 3.96 × 10^–10^). For KEGG enrichment analysis, we totally found 13 significant KEGG pathways (Fig. 3, Table S6), such as JAK-STAT signaling pathway (*P* = 1.36 × 10^–6^), natural killer cell-mediated cytotoxicity (*P* = 5.72 × 10^–6^), and antigen processing and presentation (*P* = 1.45 × 10^–4^). The PPI network illustrates the detailed interaction across significant proteins identified from PWAS analysis (Fig S1).

### MR retained 97 protein-AID pairs

**Fig. 4:**
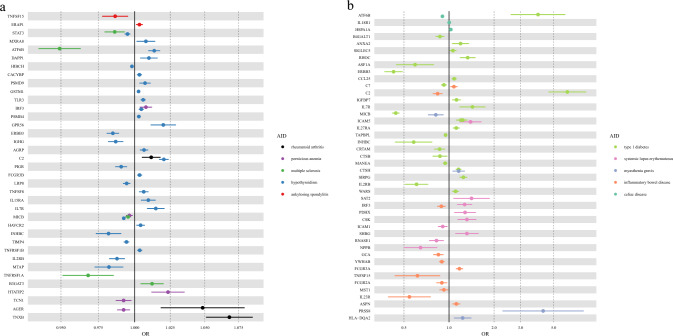
Forest plot of MR results for 10 autoimmune diseases. Only significant protein-AID pairs (FDR-corrected *P* < 0.05) are shown. **a** MR results for rheumatoid arthritis, pernicious anemia, multiple sclerosis, hypothyroidism and ankylosing spondylitis; **b** MR results for type 1 diabetes, systemic lupus erythematosus, myasthenia gravis, inflammatory bowel disease, celiac disease. IGHG, IGHG1|IGHG2|IGHG3|IGHG4|IGK@|IGL@

For 174 protein-AID pairs identified by PWAS, we further performed MR analysis to estimate the causal effects of proteins on AIDs (Tables S7–S12). 4 pairs were removed from the MR analysis due to the absence of suitable IVs. Overall, 955 valid IVs for 170 proteins were extracted. All *F*-statistics were greater than 10, indicating less weak instrument issue. For a total of 109 protein-AID pairs that are significantly reported from the IVW method, we performed MR-Egger analysis to test the pleiotropy as well as calculated Cochran’s Q statistic to test heterogeneity. For 74 protein-AID pairs without horizontal pleiotropy and heterogeneity, we retained the results from MR-IVW analysis as the final MR results. For 3 protein-AID pairs with significant pleiotropy but without heterogeneity, we retained the results from MR-Egger analysis as the final MR results. For the remaining 32 protein-AID pairs, we retained the results from a weighted median method as the final MR results. MR-steiger tests also suggested no reverse causality issue. Finally, we obtained 97 protein-AID pairs that were significant from MR analysis after FDR correction (Fig. 4), among which the *P* values from the Cochran Q statistic of 21 pairs were less than 0.05, with that of 11 pairs being in the range of 0.01–0.05. In addition, we assessed the robustness of the MR estimates by leave-one-out analyses (Fig S2). These pairs had consistent effect directions in both MR analysis and PWAS analysis, with 54 pairs showing positive associations and 43 pairs showing negative associations.

### Colocalization highly supported causal roles of 21 protein-AID pairs

Spurious MR findings might be existed when protein and diseases were driven by distinct variants with LD. Therefore, we implemented colocalization for 97 protein-disease pairs identified from MR analysis to further remove those pairs that were more likely to be biased by LD. We finally identified 21 protein-AID pairs showing strong evidence of colocalization (PP.H4 > 0.75) in six AIDs (3 for hypothyroidism, 5 for IBD, 2 for MG, 1 for PA, 2 for SLE, and 8 for T1D), among which protein encoded by CTSH is associated with both T1D and MG, with details provided in Table S13 and Fig S3. Table 1 summarized the details of the final 21 protein-AID pairs, among which 11 were confirmed in previous GWAS studies, 10 were our newly discovered potential candidate proteins. More importantly, these 20 proteins are distributed on 20 approximately independent LD blocks/regions across the whole genome partitioned by LDetect (Berisa and Pickrell 2016), indicating that these proteins are not affected by LD and may become therapeutic targets, at least among these six AIDs.

**Table 1:** Final 21 protein-AID pairs identified from the comprehensive analysis

Autoimmune diseases	Protein	Gene	Chr	P0	P1	PWAS		MR						coloc	Druggability
						Z	P	method	Num-SNP	OR	P.est	P.int	Q_pval	PP.H4.abf	
IBD	SeqId_12594_5	GCA	2	162318840	162371595	− 4.2868	1.81E-05	IVW	2	0.8493	3.78E-05	NA	0.180328	0.988491	No
IBD	SeqId_2968_61	TNFSF15	9	114784652	114806039	− 4.7537	2.00E-06	Wald Ratio	1	0.6154	6.48E-03	NA	NA	0.845131	Yes
IBD	SeqId_3309_2	FCGR2A^a^	1	161505430	161524013	− 7.2445	4.34E-13	IVW	13	0.8954	1.06E-02	0.332330	0.535824	0.995105	Yes
IBD	SeqId_4407_10	MST1	3	49683947	49689501	− 5.7579	8.52E-09	IVW	16	0.9242	2.36E-02	0.495304	0.083882	0.968925	Yes
IBD	SeqId_5088_175	IL23R^a^	1	67138907	67259979	4.3489	1.37E-05	Wald Ratio	1	0.5439	2.93E-04	NA	NA	0.951112	Yes
SLE	SeqId_17151_84	IRF3	19	49659569	49665875	3.7583	1.71E-04	IVW	5	1.2709	5.33E-05	0.654107	0.938358	0.906870	Yes
SLE	SeqId_7655_11	NPPB	1	11857464	11858945	− 3.5652	3.64E-04	IVW	2	0.6449	8.73E-04	NA	0.209240	0.837691	Yes
T1D	SeqId_13381_49	B4GALT1	9	33104082	33167356	− 3.9324	8.41E-05	IVW	5	0.8696	3.55E-05	0.692757	0.972074	0.834661	Yes
T1D	SeqId_17764_108	RHOC	1	112701127	112707434	4.3924	1.12E-05	IVW	2	1.3328	3.15E-06	NA	0.185641	0.975963	No
T1D	SeqId_18172_71	ASF1A	6	118894152	118909171	− 3.5083	4.51E-04	Wald Ratio	1	0.5933	4.51E-04	NA	NA	0.750630	Yes
T1D	SeqId_2705_5	CCL25	19	8052318	8062660	4.1236	3.73E-05	IVW	5	1.0829	7.60E-05	0.440466	0.814768	0.949666	Yes
T1D	SeqId_5132_71	IL27RA	19	14031762	14053218	3.7214	1.98E-04	EGGER	10	1.1180	3.57E-05	0.036769	0.900984	0.982888	Yes
T1D	SeqId_6408_2	INHBC	12	57434784	57452062	− 3.7108	2.07E-04	Wald Ratio	1	0.5809	2.07E-04	NA	NA	0.765056	Yes
T1D	SeqId_8465_52	CTSH	15	78921058	78949574	9.9991	1.54E-23	IVW	10	1.1577	4.19E-11	0.555035	0.112182	0.995899	Yes
T1D	SeqId_9241_40	SIRPG	20	1629152	1657779	7.7212	1.15E-14	median	5	1.2471	3.38E-13	0.719785	0.024377	0.873790	Yes
Hypothyroidism	SeqId_16918_198	TLR3^a^	4	186069155	186088073	7.0386	1.94E-12	IVW	7	1.0059	5.15E-12	0.768494	0.122773	0.999990	Yes
Hypothyroidism	SeqId_2744_57	IGHG	14	105736343	105743071	− 5.7473	9.07E-09	Wald Ratio	1	0.9869	9.26E-07	NA	NA	0.912766	No
Hypothyroidism	SeqId_5089_11	IL7R	5	35852695	35879603	4.7445	2.09E-06	Wald Ratio	1	1.0148	2.09E-06	NA	NA	0.813160	Yes
MG	SeqId_15513_108	PRSS8	16	31131433	31135727	4.5377	5.69E-06	Wald Ratio	1	4.2519	5.69E-06	NA	NA	0.981584	Yes
MG	SeqId_8465_52	CTSH	15	78921058	78949574	3.7732	1.61E-04	IVW	10	1.1601	2.22E-03	0.355898	0.403674	0.893599	Yes
PA	SeqId_19614_8	TCN1^a^	11	59852800	59866489	− 7.3087	2.70E-13	IVW	4	0.9923	6.69E-03	0.904645	0.075322	0.999981	Yes

Gene position (P0 and P1) are based on GRCh38/hg38

*Chr* chromosome, *PWAS* proteome-wide association study, *MR* Mendelian Randomization, *SNP* single nucleotide polymorphism, *OR* odd ratio, *P.est* the nominal p-value of MR, *P.int* p-value of the intercept term in the MR-egger method, *Q_pval* p-value of Cochran’s Q statistic, *IGHG* IGHG1|IGHG2|IGHG3|IGHG4|IGK@|IGL@, *IBD* inflammatory bowel disease, *SLE* systemic lupus erythematosus, *T1D* type 1 diabetes, *MG* myasthenia gravis, *PA* pernicious anemia

^a^The encoded proteins that have been found to have corresponding drugs through DGIdb

### Candidate druggable targets

As most drugs exert their therapeutic effects through targeting proteins, we finally explored whether the 20 proteins identified through the comprehensive analysis can serve as potential therapeutic targets. In DGIdb, through drug-gene interactions, we identified 13 interactions between four protein-coding genes (TLR3, FCGR2A, IL23R, TCN1) and 13 drugs (Table S14). Through druggability explorations, we identified 17 protein-coding genes, which are the potential targets for drug therapy intervention (Table S15). These findings are expected to promote and facilitate the development of specific drugs for AIDs.

## Discussion

In this study, we performed comprehensively protein-centric omics integration analysis by sequentially using PWAS, MR and colocalization analyses to identify the plasma proteins that are associated with multiple AIDs. A total of 174 protein-AID associations were identified by PWAS, which had been demonstrated to be unique to PWAS and not simply genes in close proximity or results from TWAS by performing conditional analysis. Enrichment analysis illustrated that AIDs-associated plasma proteins were significantly enriched immune-related biological process and pathways, such as regulation of lymphocyte activation (*P* = 2.63 × 10^–17^), regulation of leukocyte mediated immunity (*P* = 1.94 × 10^–12^), and inflammatory response (*P* = 3.96 × 10^–10^). Further MR and colocalization analysis screened out 21 protein-disease pairs in six AIDs, among which protein encoded by CTSH is associated with both T1D and MG. Of note, the 20 proteins are distributed on 20 approximately independent LD blocks across the whole genome and are more likely to be biologically plausible. Further explorations showed that four proteins have corresponding drugs, and 17 proteins have druggability. Our findings can advance the understanding of different genetic basis of AIDs and indicate potential specific drug targets for AIDs.

Our findings are more likely to be biologically plausible, including 11 protein-AID pairs also identified in previous GWAS studies and 10 novel protein-AID pairs identified in this study. Take T1D as an example, the protein encoded by *CTSH* is a lysosomal cysteine proteinase, which plays an important role in the overall degradation of lysosomal proteins, which in turn is closely related to the immune regulation of humans (Roberts 2005). *SIRPG* encodes members of the signal-regulatory protein (*SIRP*) family, which also belongs to the immunoglobulin superfamily, and plays a key role in the transendothelial migration of T-cells and promotes the proliferation and activation of antigen-specific T-cell (Dehmani et al. 2021; Piccio et al. 2005; Stefanidakis et al. 2008). The pancreas of people with T1D produces little or no insulin. Insulin is secreted by pancreatic β-cell, without insulin, blood sugar cannot enter cells and accumulate in the blood (Barnett 2018). Studies have shown that the products of *CCL25* bind to the chemokine receptor *CCR9* and promote cytokine-induced apoptosis by inhibiting insulin secretion, thereby impacting pancreatic β-cell function (Atanes et al. 2020), which may be a way that *CCL25* protein plays a role in the development of T1D. *RHOC* encodes a member of the Rho family of small GTPases (Bishop and Hall 2000), it was be found to be a strong inducer of *ROCK* (Okin and Medzhitov 2016; Wheeler and Ridley 2004), which is involved in the pathogenesis of diabetic complications, and its inhibitors are considered to be a promising target for the treatment of diabetic complications (Tian et al. 2021; Zhou and Li 2010). Take hypothyroidism as another example, the protein encoded by *TLR3* is a member of the Toll-like receptor (TLR) family and plays a fundamental role in pathogen recognition and activation of innate immunity (Chen et al. 2021). The protein encoded by *IL7R* is a receptor for interleukin-7 (*IL7*), which has been shown to play a key role in V (D) J recombination during lymphocyte development (Barata et al. 2019). *IL7R* deficiency may be associated with severe combined immunodeficiency (SCID) (Puel et al. 1998). Hypothyroidism occurs when the thyroid gland does not produce enough thyroid hormone. *IGHG1|IGHG2|IGHG3|IGHG4|IGK@|IGL@* is a group of genes encoding immunoglobulin G (IgG) subtypes. IgG is one of the most abundant proteins in human serum and plays a pivotal role in human immune function (Vidarsson et al. 2014). Studies have shown that the levels of IgG1 and IgG4 are higher in patients with hypothyroidism, and IgG is likely to inhibit the binding of thyroid-stimulating hormone (TSH) to its receptors by competing with TSH for receptors, resulting in decreased thyroxine secretion, thereby causing hypothyroidism (Jansson et al. 1986; Kraiem et al. 1992; Mckenzie and Zakarija 1992; Silva et al. 2003). Take MG as a final example, MG is caused by disruption of normal communication between nerves and muscles and is characterized by any muscle weakness and rapid fatigue under voluntary control (Gwathmey and Burns 2015). *PRSS8* stimulates epithelial sodium channel (ENaC) activity by activating cleavage of the gamma subunits (*SCNN1G*) (Shipway et al. 2004), and the protein it encodes may influence MG through this process. Our results suggest that reducing plasma *PRSS8* levels has a protective effect on MG, which may warrant future research.

The druggability exploration analysis showed that 4 proteins have corresponding drugs, such as *FCGR2A* and *IL23R*, which are associated with IBD. The protein encoded by *FCGR2A* is a cell surface receptor present on phagocytes such as macrophages and neutrophils, and is involved in the process of phagocytosis and clearance of immune complexes. A study has shown *FCGR2A* is one of the key driver genes of IBD (Peters et al. 2017) and that drugs related to *FCGR2A* such as adalimumab, etanercept, and infliximab are available for the treatment of IBD. These drugs can also be used to treat RA, AS, and other AIDs. Infliximab, for example, is a TNF inhibitor that is routinely used to treat patients with rheumatic diseases, psoriasis, and IBD. However, recent studies have shown that some IBD patients do not respond well to TNF inhibitors. For instance, in IBD, disturbances in the gut microbial network that produce short-chain fatty acids as carbon sources for intestinal epithelial cells and induction of regulatory T cells are associated with poor responsiveness to TNF inhibitors (Yilmaz et al. 2019). Overexpression of the IL-7 receptor (*IL-7R*) signaling pathways in the colon have also been found in a mouse models to be associated with no response to IBD anti-TNF therapy (Belarif et al. 2019). Single-cell analysis of inflammatory tissues from patients with Crohn's disease has revealed a unique cellular module associated with the ineffectiveness of TNF inhibitors (Martin et al. 2019). All of this suggested that, due to different pathogenic mechanisms, drugs used to treat a variety of diseases have some limitations in treating a specific disease. Other proteins, meanwhile, have shown advantages in treating specific diseases. Both our results and GWAS showed that multiple variants of *IL23R* were significantly associated with IBD and suggested that blocking the IL-23 signaling pathway may be a reasonable treatment strategy for IBD (Duerr et al. 2006). This has also been shown in biopharmaceuticals studies targeting the IL-23/IL-17 axis. Experimental studies related to IBD have shown that IL-23 drives local intestinal inflammation, and blockade of IL-23 or its receptor *IL23R* is associated with impaired activation of IL-23 target cells (such as TH17 cells, ILC3s, granulocytes, and natural killer cells) and reduced production of pro-inflammatory cytokines (Neurath 2019; Uhlig et al. 2006). This mechanism can be used for drug treatment of IBD. Clinical trials of monoclonal antibodies against interleukin-23, such as Ustekinumab and risankizumab, have shown efficacy and safety in IBD patients (Feagan et al. 2017, 2016; Sandborn et al. 2012; Sands et al. 2017). Disease mechanism studies have also shown that IL-23 is a key cytokine for effective drug treatment of IBD compared with IL-12 and IL-17 (Cua et al. 2003; Yen et al. 2006). All the results suggest that the druggable proteins identified in this study have the potential to be effective specific target proteins for AID, which can benefit to drug development of AIDs.

Our study is not without limitations. First, it is inadequate to only consider protein levels in peripheral blood without involving protein levels in other tissues and organs. We have searched the publicly available pQTL datasets in other tissues or organs, and only 2 pQTL datasets in brain tissue can be found to be available (Beach et al. 2015; Wingo et al. 2021b). However, the maximum sample size for these brain pQTL data is 376 (Wingo et al. 2021b), which could restrict the power for the omics integration analysis. Second, the limitations regarding the MR sensitivity analysis should be well documented. Although MR-Egger allows for the detection and correction of directional pleiotropy, it requires the strict Egger assumption that all SNPs have the same horizontal pleiotropy effects. Besides, MR results with only one IV should be interpreted with caution due to lack of sensitivity analysis. Cochran's Q statistic tends to be sensitive and often requires a large sample size. In addition, the investigation of heterogeneity of causal estimates as an assessment of the instrumental variable assumptions relies on the assumption that all valid instrumental variables identify the same causal parameter. If not, then the heterogeneity test may over-reject the null (Burgess et al. 2017). Third, we only focused on European ancestry due to the large-scale pQTL data and GWASs of AIDs were only available for the European population and the findings cannot be directly extended to other populations.

In summary, we identified several plasma proteins that are associated with AIDs from comprehensive omics integration analysis and highlighted the potential of these proteins to develop as therapeutic targets for AIDs, indicating the drug development for AIDs could be developed in a disease-specific manner. Further experimental studies should be conducted to validate these findings.

## Supplementary Information

Below is the link to the electronic supplementary material.

Supplementary file1 (XLSX 173 KB)—Table S1. GWAS summary statistics with European ancestry for AIDs. Table S2. 174 protein-AID pairs identified from PWAS. Table S3. Bivariate conditional analysis of significant PWAS genes and all TWAS genes in whole blood. Table S4. Bivariate conditional analysis of PWAS and TWAS with same genes in whole blood. Table S5. Significant GO terms through GO enrichment analysis for PWAS-significant proteins. Table S6. Significant KEGG pathways through KEGG enrichment analysis for PWAS-significant proteins. Table S7. Results of MR-Steiger directionality test. Table S8. Results of MR-IVW. Table S9. Results of MR-egger. Table S10. Results of Weighted Median MR. Table S11. Results of heterogeneity assessment through Cochran's Q statistic. Table S12. 95 protein-AID pairs identified from MR analysis. Table S13. 21 protein-AID pairs identified from colocalization analysis. Table S14. Drug-gene interactions between 4 protein-coding genes and 13 drugs. Table S15. Druggability of 20 significant proteins identified from the comprehensive analysis.

Supplementary file2 (PDF 8691 KB)—Fig S1. The PPI network for PWAS significant proteins

Supplementary file3 (PDF 902 KB)—Fig S2. Forest plots of leave-one-out results.

Supplementary file4 (PDF 1170 KB)—Fig S3. LocusZoom plots of 21 protein-AID pairs identified from colocalization.

## Data Availability

The datasets generated and/or analysed during the current study are publicly available. GWAS summary data for AIDs are available from the GWAS Catalog at https://www.ebi.ac.uk/gwas/ and from the Program in Complex Trait Genomics at https://cnsgenomics.com/content/data. The plasma pQTL data are available at http://nilanjanchatterjeelab.org/pwas/. The enrichment analysis was done in the Metascape website at https://metascape.org/gp/index.html. The PPI network exploration is implemented based on the STRING database at https://cn.string-db.org/. The Phenoscanner database is available at http://www.phenoscanner.medschl.cam.ac.uk/. Druggable targets exploration was done through the Drug-Gene Interaction Database (DGIdb) at https://www.dgidb.org/.
