# Supplementary material for: Protein-centric omics integration analysis identifies candidate plasma proteins for multiple autoimmune diseases
Source: Hum Genet. 2023 Dec 24;143(9-10):1035–48. doi: 10.1007/s00439-023-02627-0 (PMC11485194; doi:10.1007/s00439-023-02627-0)
Supplement: Supplementary file 2 — Supplementary file2 (PDF 8691 KB)—Fig S1. The PPI network for PWAS significant proteins [file 439_2023_2627_MOESM2_ESM.pdf]

Supplementary File 2:

Protein-centric omics integration analysis identifies candidate plasma proteins for multiple autoimmune diseases

Yingxuan Chen[1,2†], Shuai Liu[1,2†], Weiming Gong[1,2], Ping Guo[1,2], Fuzhong Xue[1,2], Xiang Zhou[3,4], Shukang Wang[1,2*], Zhongshang Yuan[1,2*]

[1] Department of Biostatistics, School of Public Health, Cheeloo College of Medicine, Shandong University, 44, Wenhua West Road, Jinan, Shandong, 250012, China.
[2] Institute for Medical Dataology, Shandong University, 12550, Erhuan East Road, Jinan, Shandong, 250003, China.
[3] Department of Biostatistics, University of Michigan, Ann Arbor, MI 48109, USA.
[4] Center for Statistical Genetics, University of Michigan, Ann Arbor, MI 48109, USA.

[†] Y.C. and S.L. contributed equally to this work.

* Joint correspondence authors to:
Prof Shukang Wang
Department of Biostatistics, School of Public Health, Cheeloo College of Medicine, Shandong University, 44, Wenhua West Road, Jinan, Shandong, 250012, China.
Email: wsk2001@sdu.edu.cn
Prof Zhongshang Yuan
Department of Biostatistics, School of Public Health, Cheeloo College of Medicine, Shandong University, 44, Wenhua West Road, Jinan, Shandong, 250012, China.
Email: yuanzhongshang@sdu.edu.cn

Figure S1: The PPI network for PWAS significant proteins

The Network type is full STRING network (the edges indicate both functional and physical protein associations). The meaning of network edges is evidence (line color indicates the type of interaction evidence). The minimum required interaction score is medium confidence (0.400).

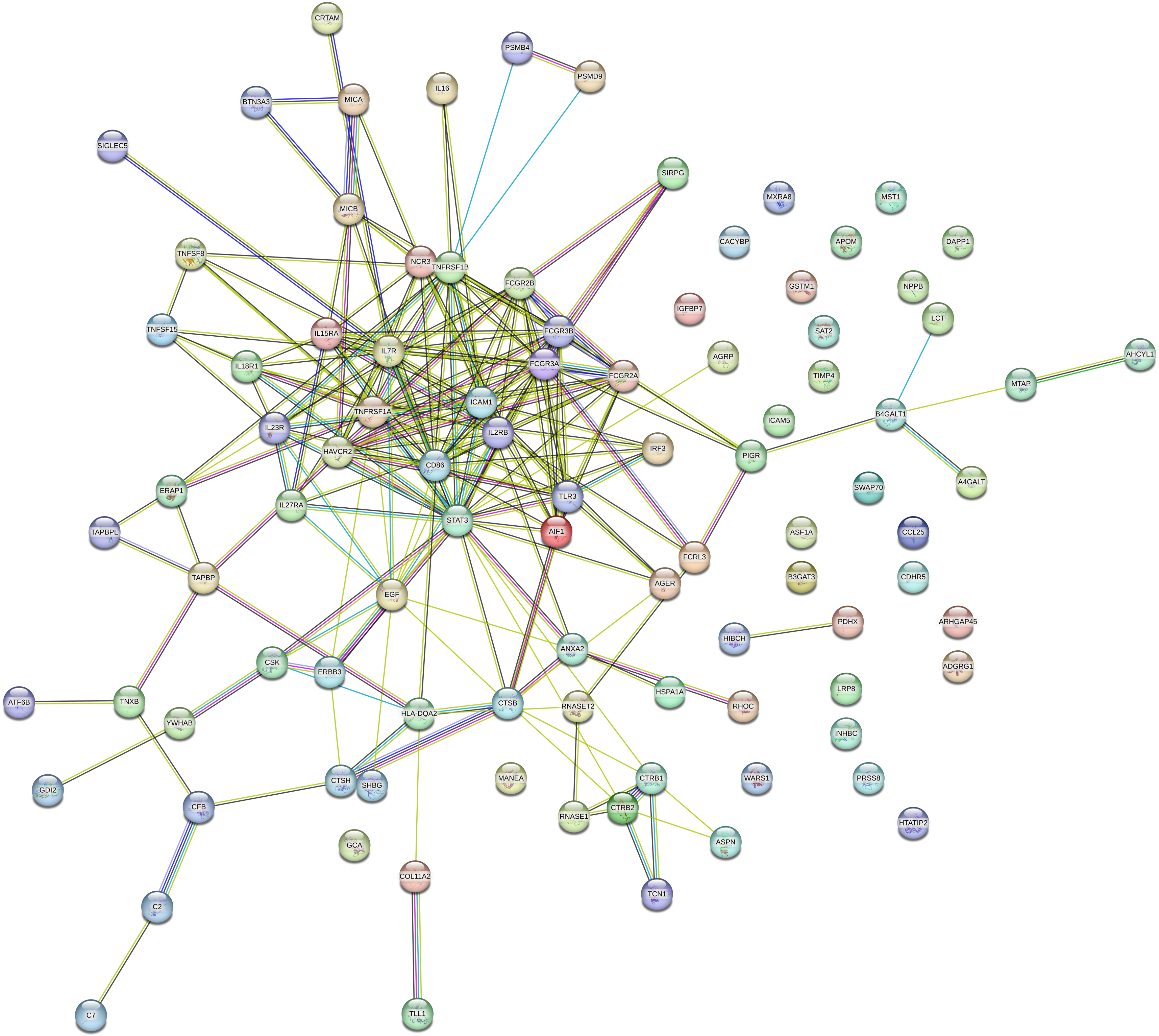
CRTAM
PSMB4
PSMD9
IL16
BTN3A3
MICA
SIGLEC5
SIRPG
MXRA8
MST1
MICB
CACYBP
APOM
DAPP1
TNFSF8
NCR3
TNFRSF1B
FCGR2B
GSTM1
NPPB
IGFBP7
LCT
TNFSF15
IL15RA
FCGR3B
SAT2
AHCYL1
IL7R
AGRP
IL18R1
FCGR3A
FCGR2A
TIMP4
MTAP
ICAM1
ICAM5
B4GALT1
TNFRSF1A
IL23R
IL2RB
IRF3
HAVCR2
PIGR
CD86
SWAP70
A4GALT
ERAP1
TLR3
IL27RA
STAT3
TAPBPL
AIF1
ASF1A
CCL25
FCRL3
TAPBP
AGER
B3GAT3
CDHR5
EGF
PDHX
ARHGAP45
HIBCH
ANXA2
CSK
ERBB3
ADGRG1
LRP8
HSPA1A
ATF6B
TNXB
CTSB
RHOC
RNASET2
YWHAB
HLA-DQA2
INHBC
WARS1
PRSS8
MANEA
CTRB1
CTSH
SHBG
GDI2
CFB
RNASE1
CTRB2
HTATIP2
GCA
ASPN
COL11A2
TCN1
C2
C7
TLL1
