# Supplementary material for: Protein-centric omics integration analysis identifies candidate plasma proteins for multiple autoimmune diseases
Source: Hum Genet. 2023 Dec 24;143(9-10):1035–48. doi: 10.1007/s00439-023-02627-0 (PMC11485194; doi:10.1007/s00439-023-02627-0)
Supplement: Supplementary file 3 — Supplementary file3 (PDF 902 KB)—Fig S2. Forest plots of leave-one-out results. [file 439_2023_2627_MOESM3_ESM.pdf]

# Supplementary File 3 :

## Protein-centric omics integration analysis identifies candidate plasma proteins for multiple autoimmune diseases

Yingxuan Chen[1,2†], Shuai Liu[1,2†], Weiming Gong[1,2], Ping Guo[1,2], Fuzhong Xue[1,2], Xiang Zhou[3,4], Shukang Wang[1,2*], Zhongshang Yuan[1,2*]

[1] Department of Biostatistics, School of Public Health, Cheeloo College of Medicine, Shandong University, 44, Wenhuaxi Road, Jinan, Shandong, 250012, China.

[2] Institute for Medical Dataology, Shandong University, 12550, Erhuan East Road, Jinan, Shandong, 250003, China.

[3] Department of Biostatistics, University of Michigan, Ann Arbor, MI 48109, USA.

[4] Center for Statistical Genetics, University of Michigan, Ann Arbor, MI 48109, USA.

[†] Y.C. and S.L. contributed equally to this work.

* Joint correspondence authors to:

Prof Shukang Wang

Department of Biostatistics, School of Public Health, Cheeloo College of Medicine, Shandong University, 44, Wenhua West Road, Jinan, Shandong, 250012, China.

Email: wsk2001@sdu.edu.cn

Prof Zhongshang Yuan

Department of Biostatistics, School of Public Health, Cheeloo College of Medicine, Shandong University, 44, Wenhua West Road, Jinan, Shandong, 250012, China.

Email: yuanzhongshang@sdu.edu.cn

**Figure S2: Forest plots of leave-one-out results.**

The x-axis represents the effect size β, and the y-axis represents the SNP. The meaning of each row is the total effect size of the remaining SNP after removing the current SNP. All represents the total effect of all SNPs.

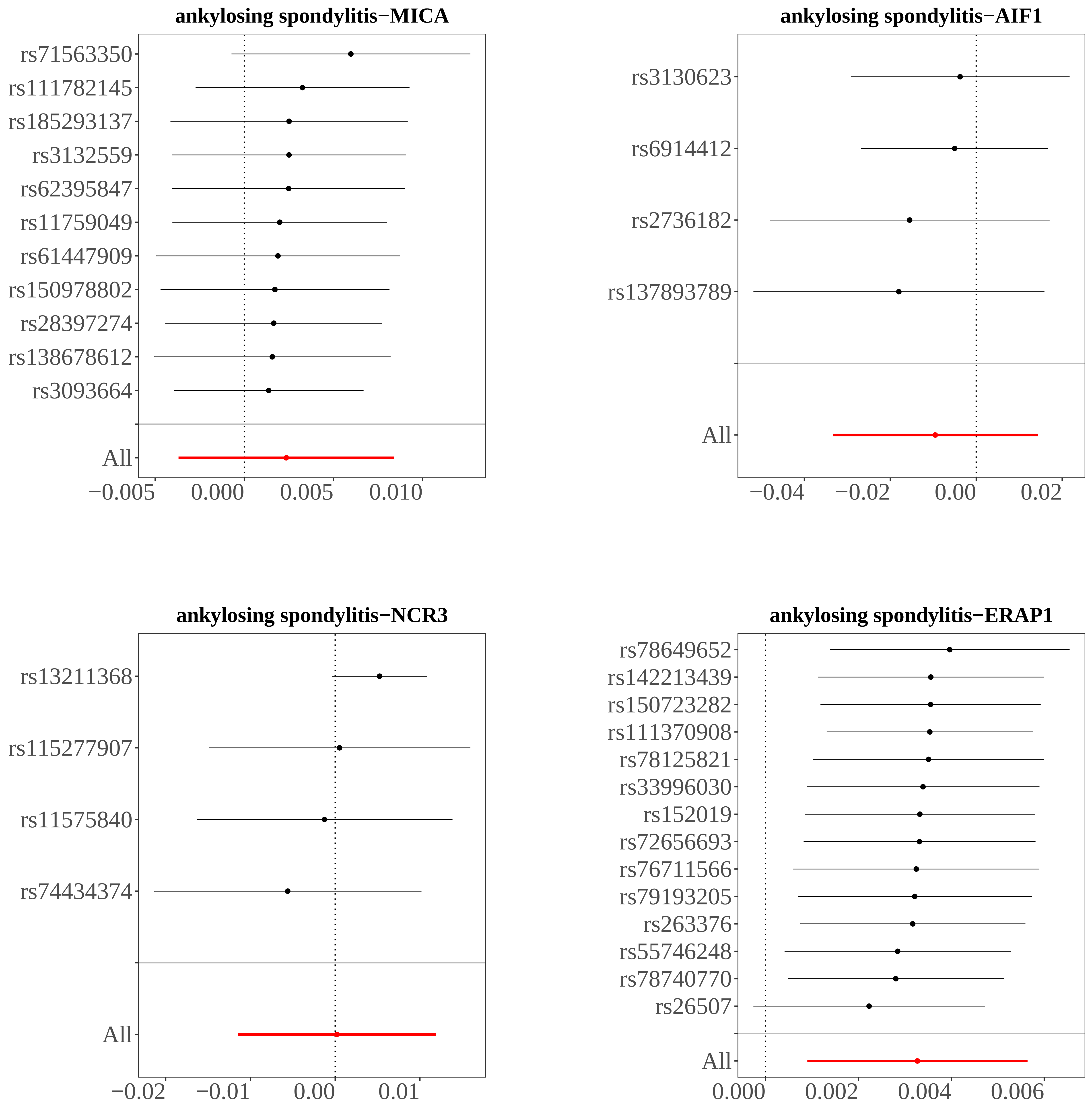
ankylosing spondylitis−MICA
rs71563350
rs111782145
rs185293137
rs3132559
rs62395847
rs11759049
rs61447909
rs150978802
rs28397274
rs138678612
rs3093664
All
−0.005
0.000
0.005
0.010
ankylosing spondylitis−AIF1
rs3130623
rs6914412
rs2736182
rs137893789
All
−0.04
−0.02
0.00
0.02
ankylosing spondylitis−NCR3
rs13211368
rs115277907
rs11575840
rs74434374
All
−0.02
−0.01
0.00
0.01
ankylosing spondylitis−ERAP1
rs78649652
rs142213439
rs150723282
rs111370908
rs78125821
rs33996030
rs152019
rs72656693
rs76711566
rs79193205
rs263376
rs55746248
rs78740770
rs26507
All
0.000
0.002
0.004
0.006

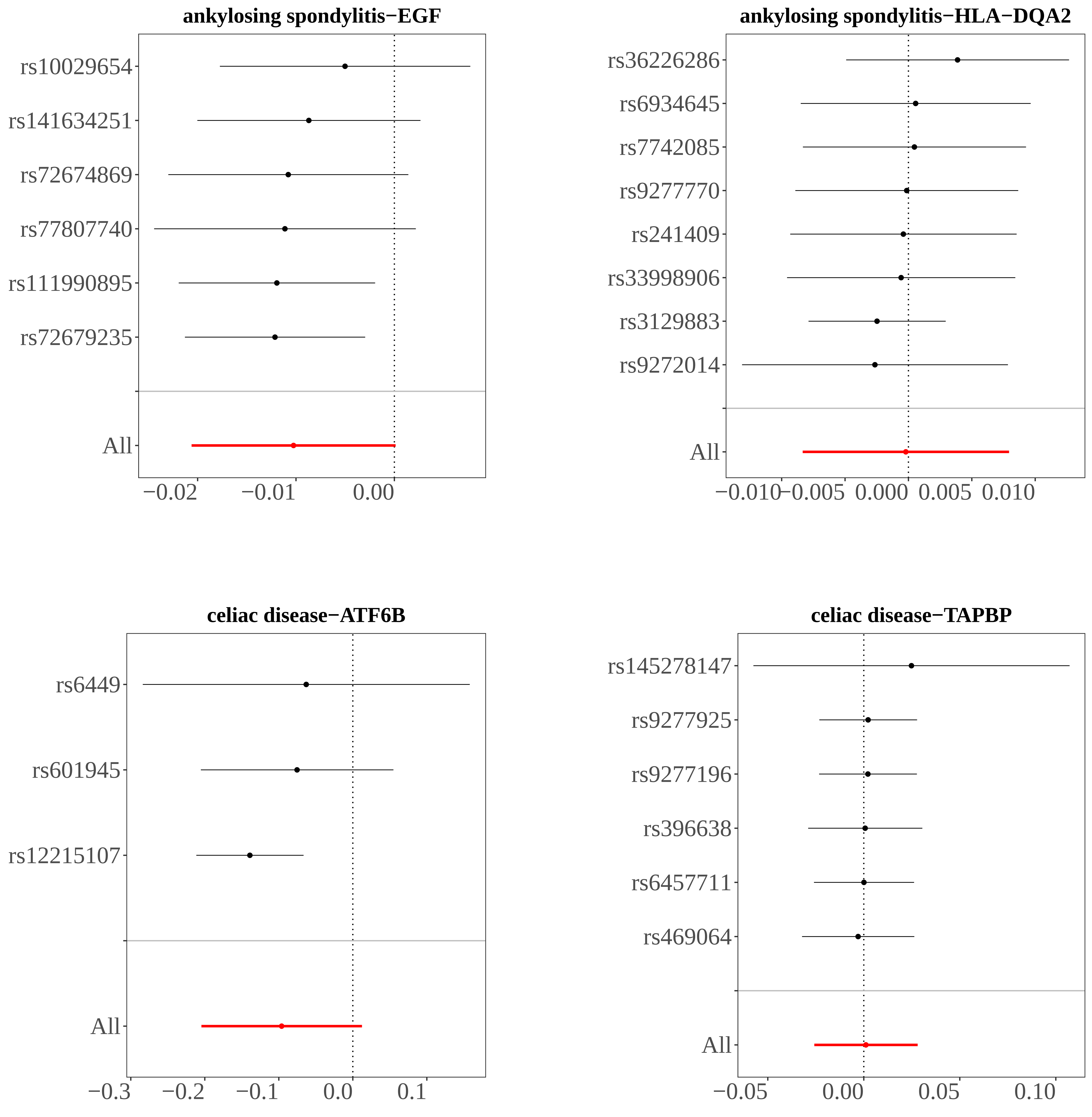
ankylosing spondylitis−EGF
rs10029654
rs141634251
rs72674869
rs77807740
rs111990895
rs72679235
All
−0.02
−0.01
0.00
ankylosing spondylitis−HLA−DQA2
rs36226286
rs6934645
rs7742085
rs9277770
rs241409
rs33998906
rs3129883
rs9272014
All
−0.010
−0.005
0.000
0.005
0.010
celiac disease−ATF6B
rs6449
rs601945
rs12215107
All
−0.3
−0.2
−0.1
0.0
0.1
celiac disease−TAPBP
rs145278147
rs9277925
rs9277196
rs396638
rs6457711
rs469064
All
−0.05
0.00
0.05
0.10

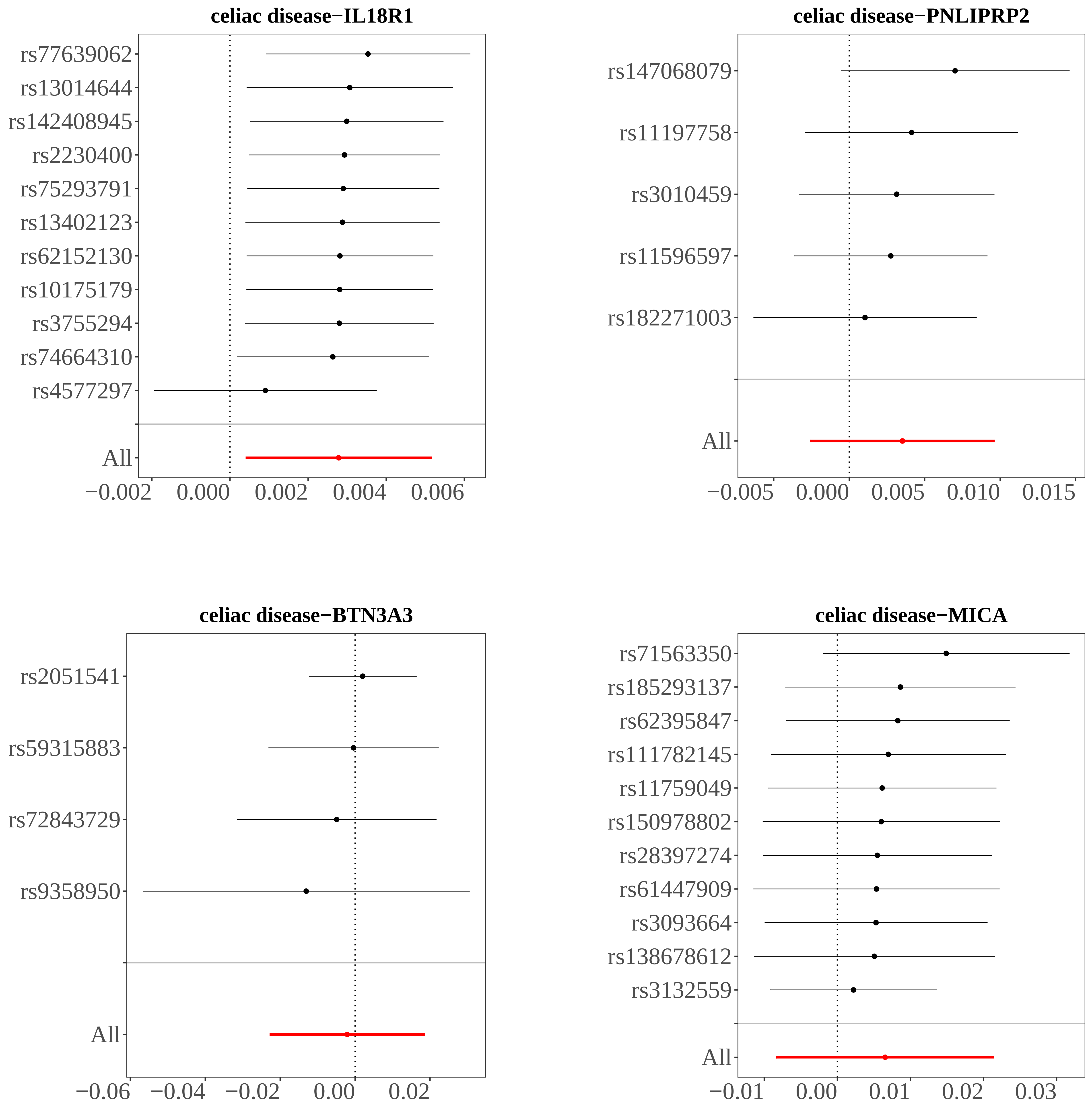
celiac disease−IL18R1
rs77639062
rs13014644
rs142408945
rs2230400
rs75293791
rs13402123
rs62152130
rs10175179
rs3755294
rs74664310
rs4577297
All
−0.002 0.000 0.002 0.004 0.006
celiac disease−PNLIPRP2
rs147068079
rs11197758
rs3010459
rs11596597
rs182271003
All
−0.005 0.000 0.005 0.010 0.015
celiac disease−BTN3A3
rs2051541
rs59315883
rs72843729
rs9358950
All
−0.06 −0.04 −0.02 0.00 0.02
celiac disease−MICA
rs71563350
rs185293137
rs62395847
rs111782145
rs11759049
rs150978802
rs28397274
rs61447909
rs3093664
rs138678612
rs3132559
All
−0.01 0.00 0.01 0.02 0.03

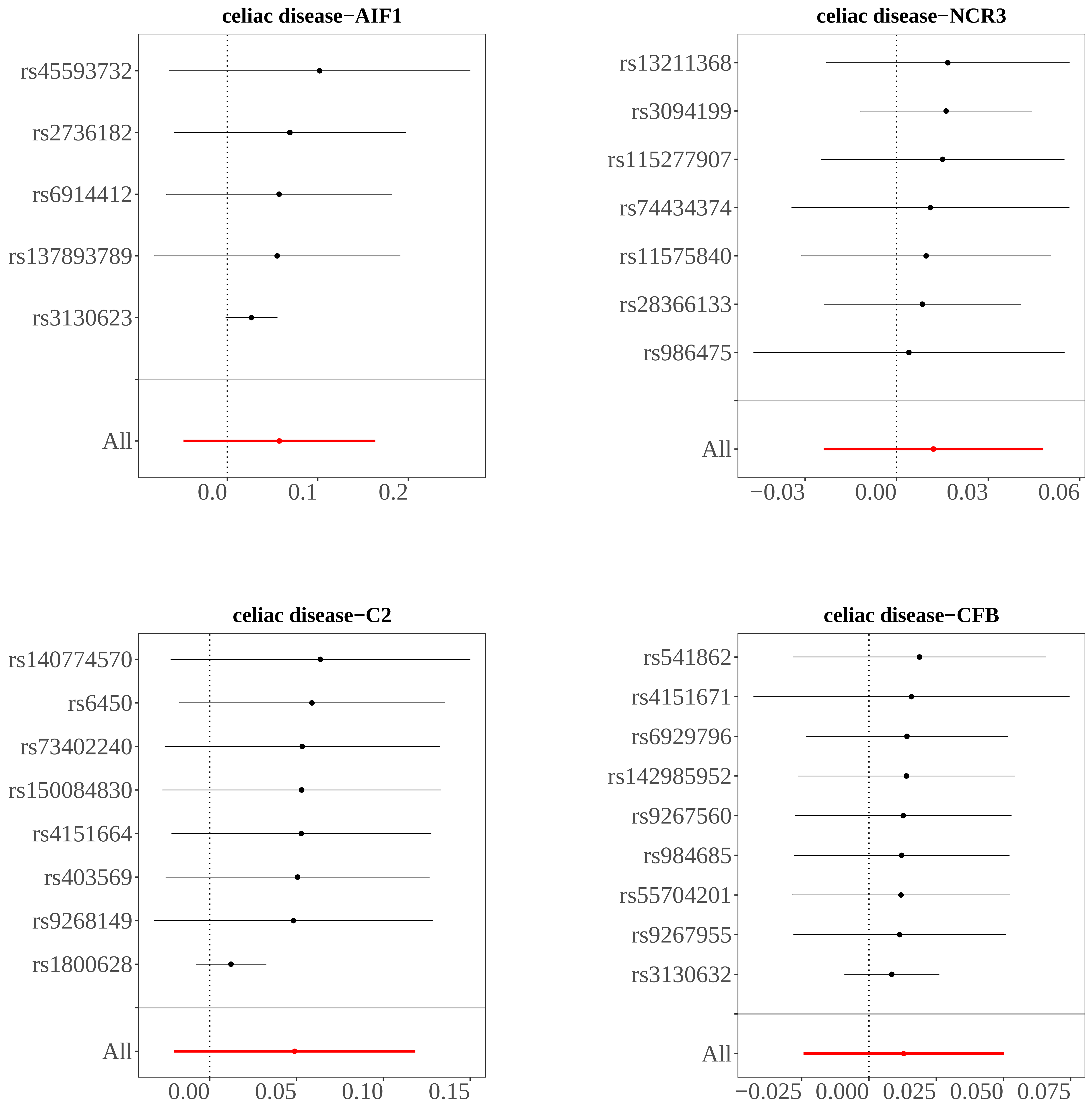
celiac disease−AIF1
rs45593732
rs2736182
rs6914412
rs137893789
rs3130623
All
0.0
0.1
0.2
celiac disease−NCR3
rs13211368
rs3094199
rs115277907
rs74434374
rs11575840
rs28366133
rs986475
All
−0.03
0.00
0.03
0.06
celiac disease−C2
rs140774570
rs6450
rs73402240
rs150084830
rs4151664
rs403569
rs9268149
rs1800628
All
0.00
0.05
0.10
0.15
celiac disease−CFB
rs541862
rs4151671
rs6929796
rs142985952
rs9267560
rs984685
rs55704201
rs9267955
rs3130632
All
−0.025
0.000
0.025
0.050
0.075

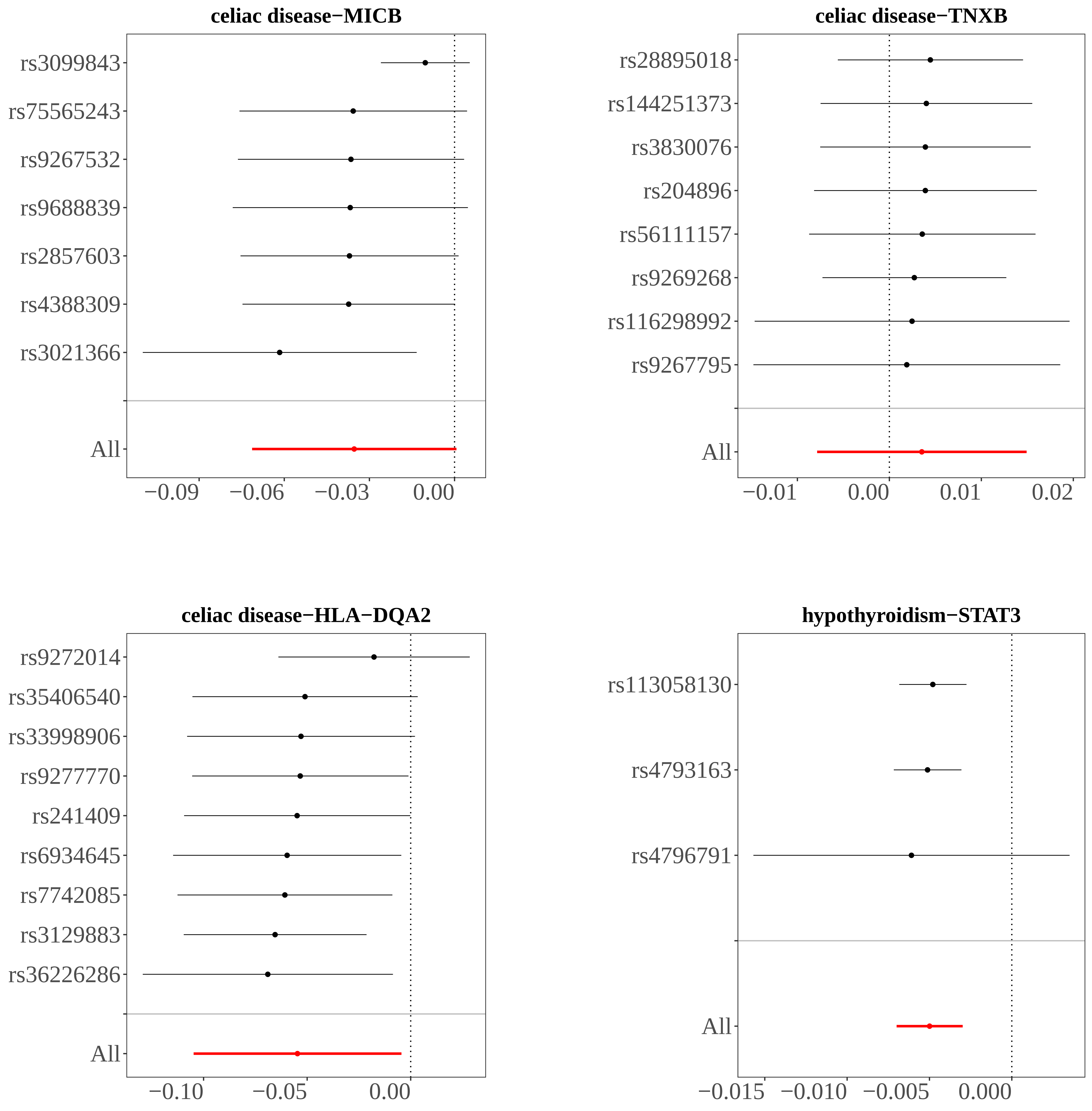
celiac disease−MICB
rs3099843
rs75565243
rs9267532
rs9688839
rs2857603
rs4388309
rs3021366
All
−0.09
−0.06
−0.03
0.00
celiac disease−TNXB
rs28895018
rs144251373
rs3830076
rs204896
rs56111157
rs9269268
rs116298992
rs9267795
All
−0.01
0.00
0.01
0.02
celiac disease−HLA−DQA2
rs9272014
rs35406540
rs33998906
rs9277770
rs241409
rs6934645
rs7742085
rs3129883
rs36226286
All
−0.10
−0.05
0.00
hypothyroidism−STAT3
rs113058130
rs4793163
rs4796791
All
−0.015
−0.010
−0.005
0.000

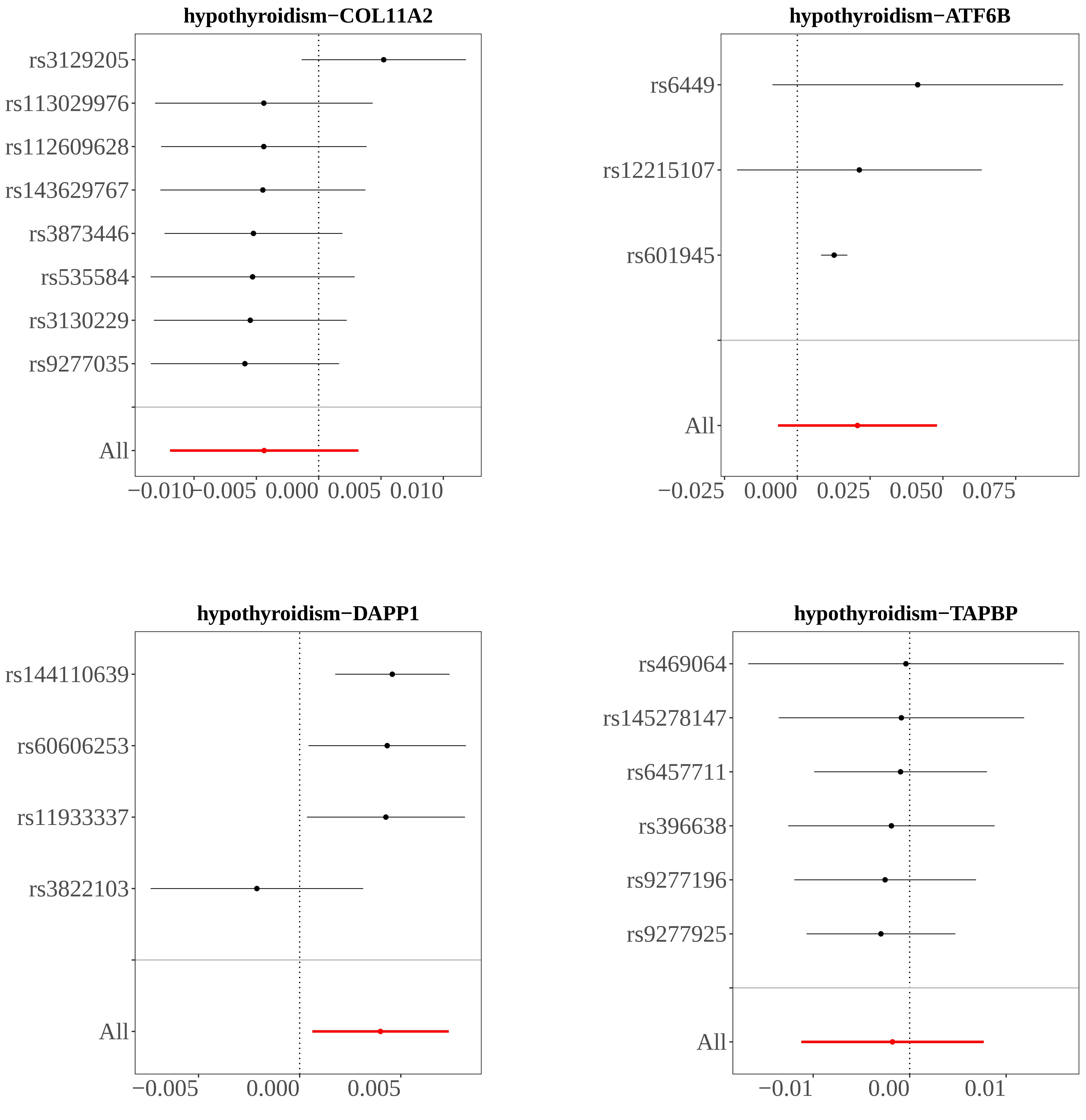
hypothyroidism−COL11A2
rs3129205
rs113029976
rs112609628
rs143629767
rs3873446
rs535584
rs3130229
rs9277035
All
−0.010
−0.005
0.000
0.005
0.010
hypothyroidism−ATF6B
rs6449
rs12215107
rs601945
All
−0.025
0.000
0.025
0.050
0.075
hypothyroidism−DAPP1
rs144110639
rs60606253
rs11933337
rs3822103
All
−0.005
0.000
0.005
hypothyroidism−TAPBP
rs469064
rs145278147
rs6457711
rs396638
rs9277196
rs9277925
All
−0.01
0.00
0.01

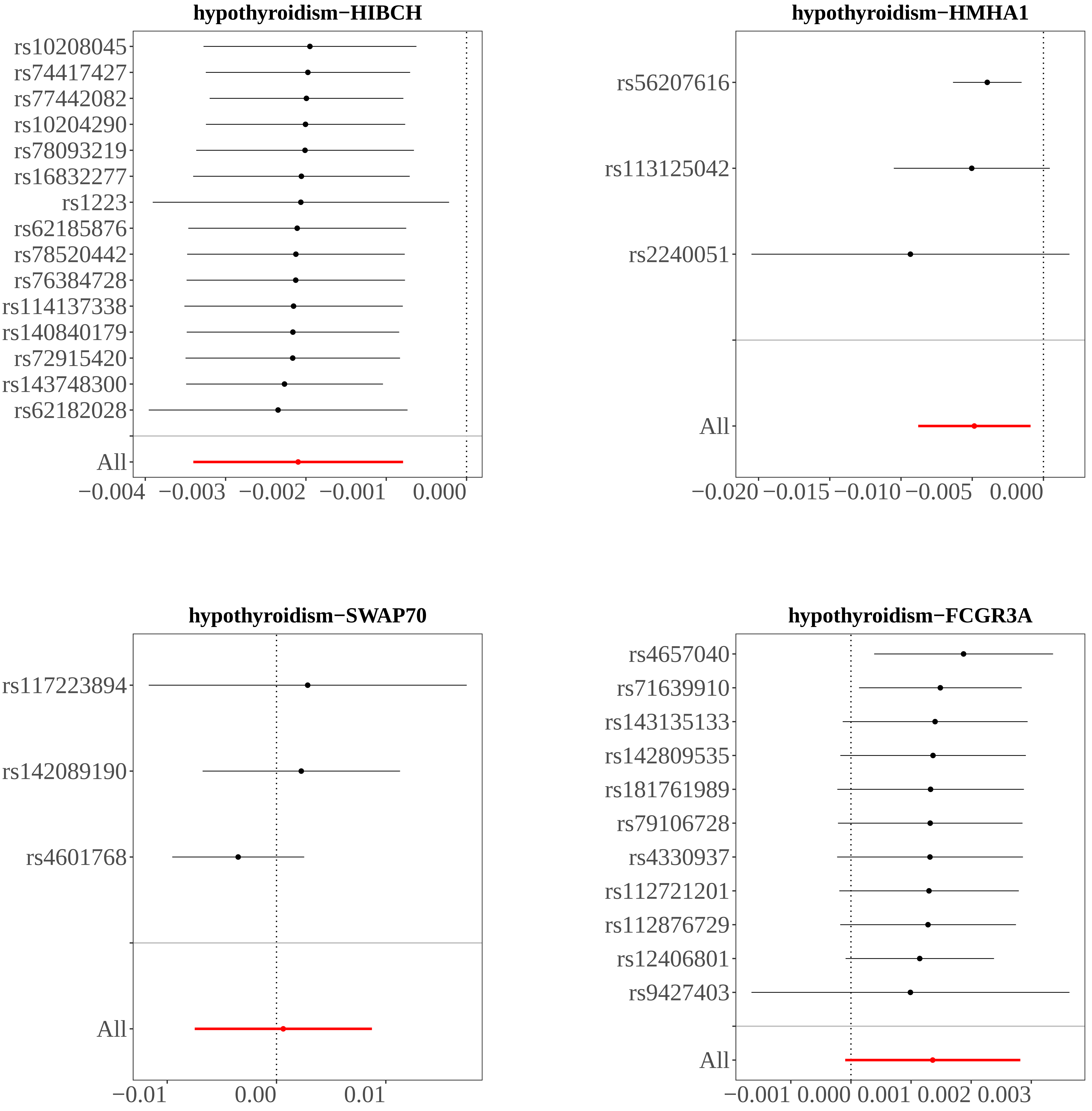

hypothyroidism−HIBCH
rs10208045
rs74417427
rs77442082
rs10204290
rs78093219
rs16832277
rs1223
rs62185876
rs78520442
rs76384728
rs114137338
rs140840179
rs72915420
rs143748300
rs62182028
All
−0.004 −0.003 −0.002 −0.001 0.000
hypothyroidism−HMHA1
rs56207616
rs113125042
rs2240051
All
−0.020 −0.015 −0.010 −0.005 0.000
hypothyroidism−SWAP70
rs117223894
rs142089190
rs4601768
All
−0.01 0.00 0.01
hypothyroidism−FCGR3A
rs4657040
rs71639910
rs143135133
rs142809535
rs181761989
rs79106728
rs4330937
rs112721201
rs112876729
rs12406801
rs9427403
All
−0.001 0.000 0.001 0.002 0.003

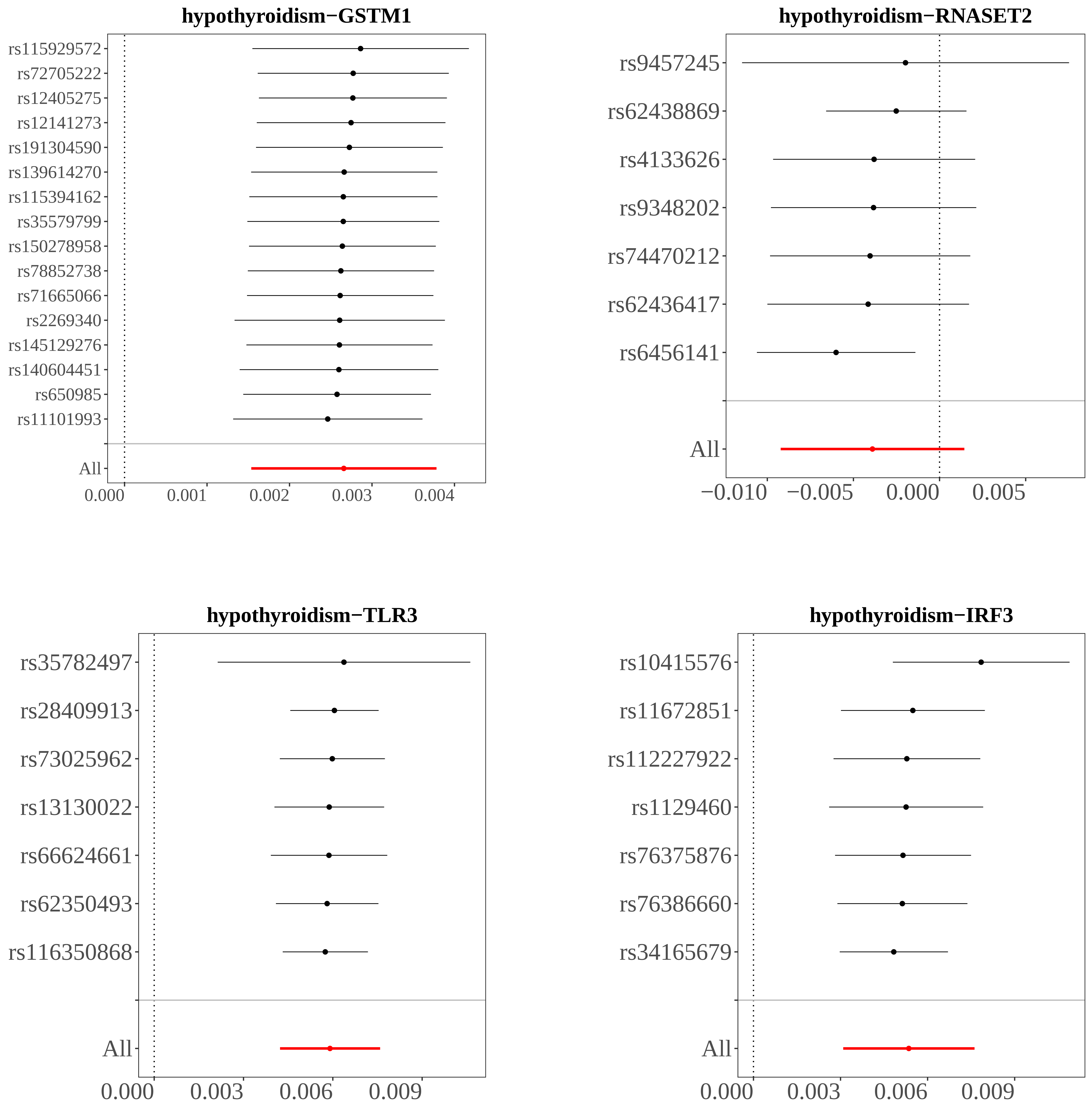
hypothyroidism−GSTM1
rs115929572
rs72705222
rs12405275
rs12141273
rs191304590
rs139614270
rs115394162
rs35579799
rs150278958
rs78852738
rs71665066
rs2269340
rs145129276
rs140604451
rs650985
rs11101993
All
0.000
0.001
0.002
0.003
0.004
hypothyroidism−RNASET2
rs9457245
rs62438869
rs4133626
rs9348202
rs74470212
rs62436417
rs6456141
All
−0.010
−0.005
0.000
0.005
hypothyroidism−TLR3
rs35782497
rs28409913
rs73025962
rs13130022
rs66624661
rs62350493
rs116350868
All
0.000
0.003
0.006
0.009
hypothyroidism−IRF3
rs10415576
rs11672851
rs112227922
rs1129460
rs76375876
rs76386660
rs34165679
All
0.000
0.003
0.006
0.009

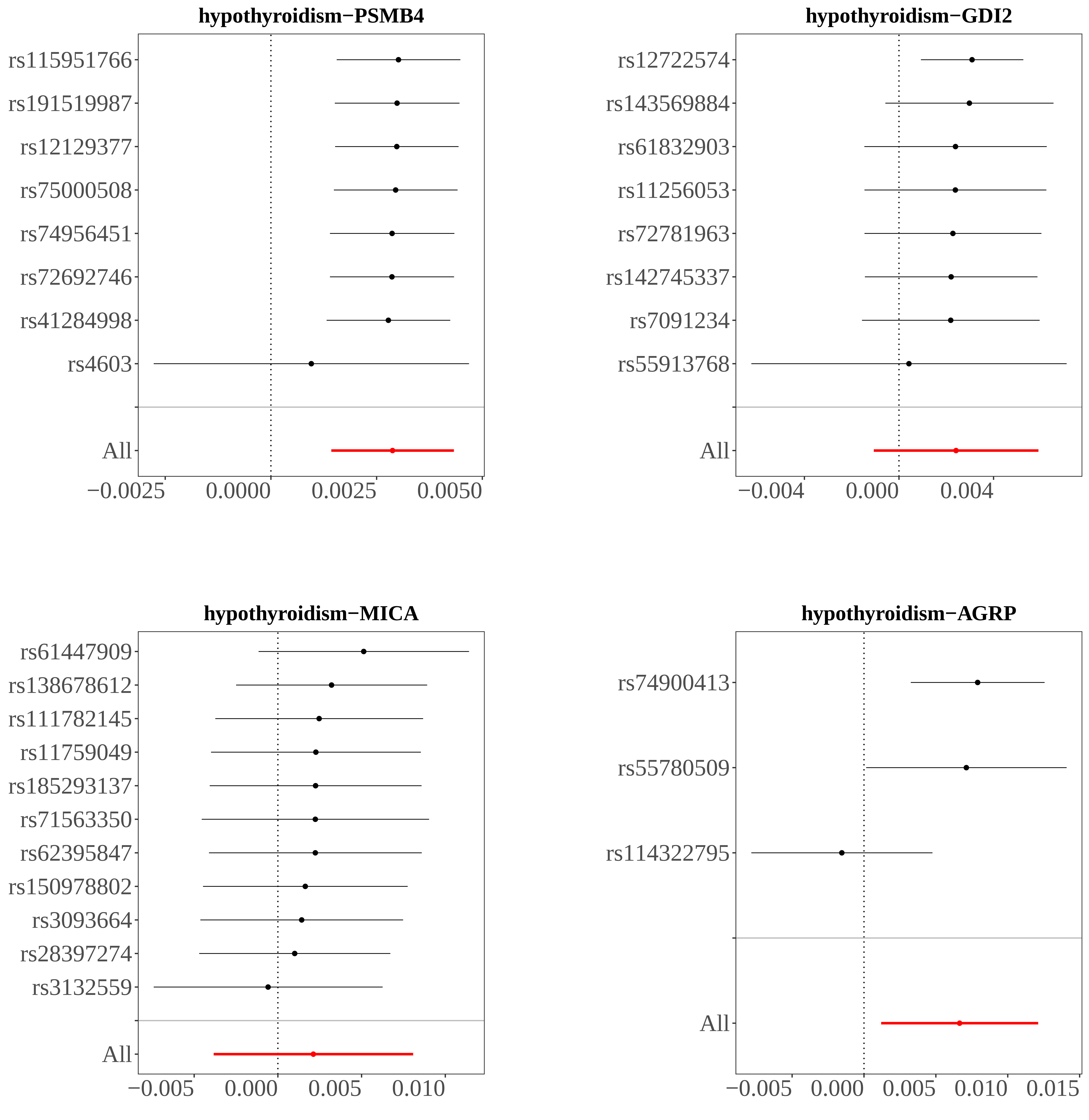
hypothyroidism−PSMB4
rs115951766
rs191519987
rs12129377
rs75000508
rs74956451
rs72692746
rs41284998
rs4603
All
−0.0025
0.0000
0.0025
0.0050
hypothyroidism−GDI2
rs12722574
rs143569884
rs61832903
rs11256053
rs72781963
rs142745337
rs7091234
rs55913768
All
−0.004
0.000
0.004
hypothyroidism−MICA
rs61447909
rs138678612
rs111782145
rs11759049
rs185293137
rs71563350
rs62395847
rs150978802
rs3093664
rs28397274
rs3132559
All
−0.005
0.000
0.005
0.010
hypothyroidism−AGRP
rs74900413
rs55780509
rs114322795
All
−0.005
0.000
0.005
0.010
0.015

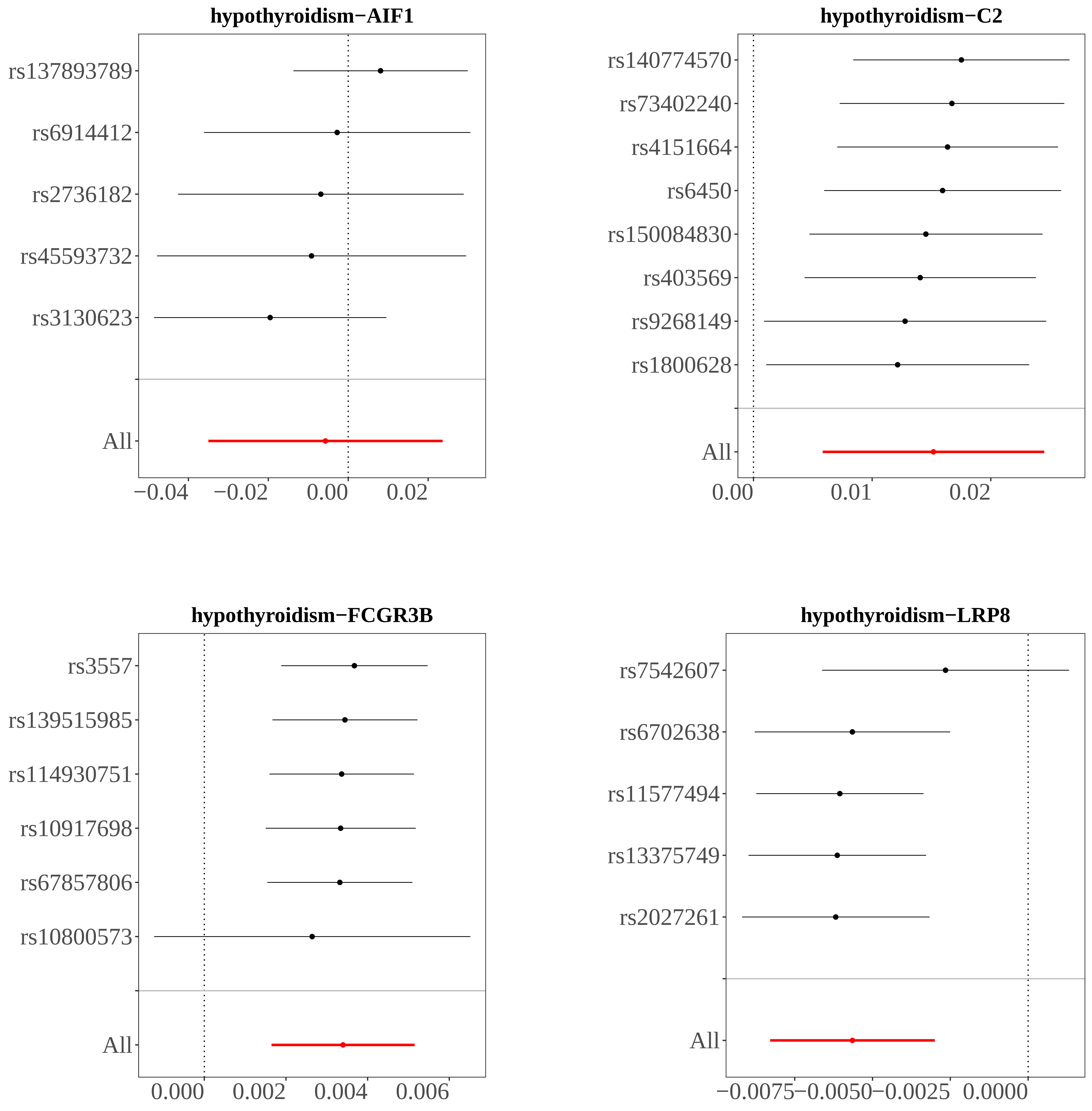

hypothyroidism−AIF1
rs137893789
rs6914412
rs2736182
rs45593732
rs3130623
All
−0.04
−0.02
0.00
0.02
hypothyroidism−C2
rs140774570
rs73402240
rs4151664
rs6450
rs150084830
rs403569
rs9268149
rs1800628
All
0.00
0.01
0.02
hypothyroidism−FCGR3B
rs3557
rs139515985
rs114930751
rs10917698
rs67857806
rs10800573
All
0.000
0.002
0.004
0.006
hypothyroidism−LRP8
rs7542607
rs6702638
rs11577494
rs13375749
rs2027261
All
−0.0075
−0.0050
−0.0025
0.0000

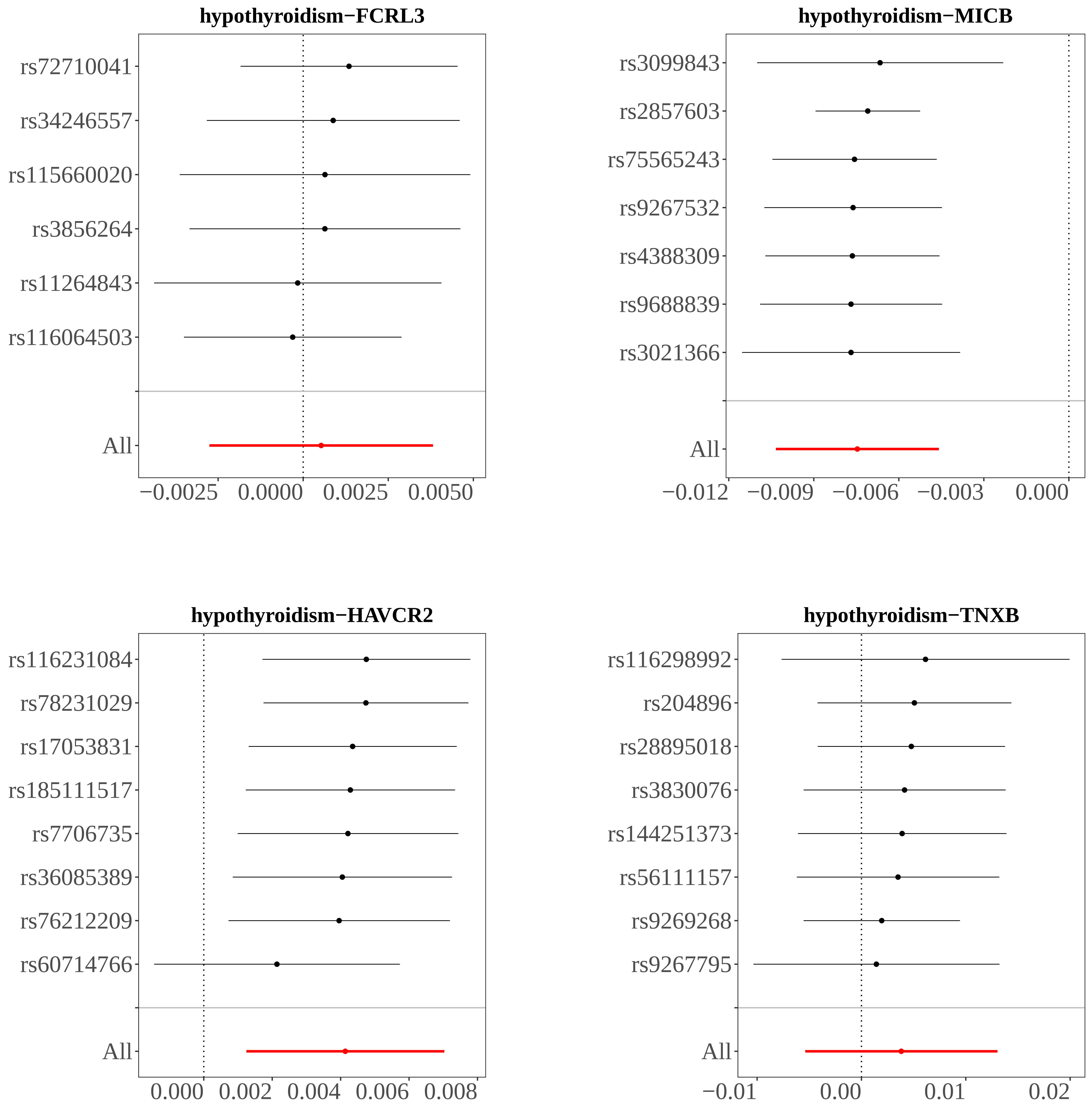
hypothyroidism−FCRL3
rs72710041
rs34246557
rs115660020
rs3856264
rs11264843
rs116064503
All
−0.0025
0.0000
0.0025
0.0050
hypothyroidism−MICB
rs3099843
rs2857603
rs75565243
rs9267532
rs4388309
rs9688839
rs3021366
All
−0.012
−0.009
−0.006
−0.003
0.000
hypothyroidism−HAVCR2
rs116231084
rs78231029
rs17053831
rs185111517
rs7706735
rs36085389
rs76212209
rs60714766
All
0.000
0.002
0.004
0.006
0.008
hypothyroidism−TNXB
rs116298992
rs204896
rs28895018
rs3830076
rs144251373
rs56111157
rs9269268
rs9267795
All
−0.01
0.00
0.01
0.02

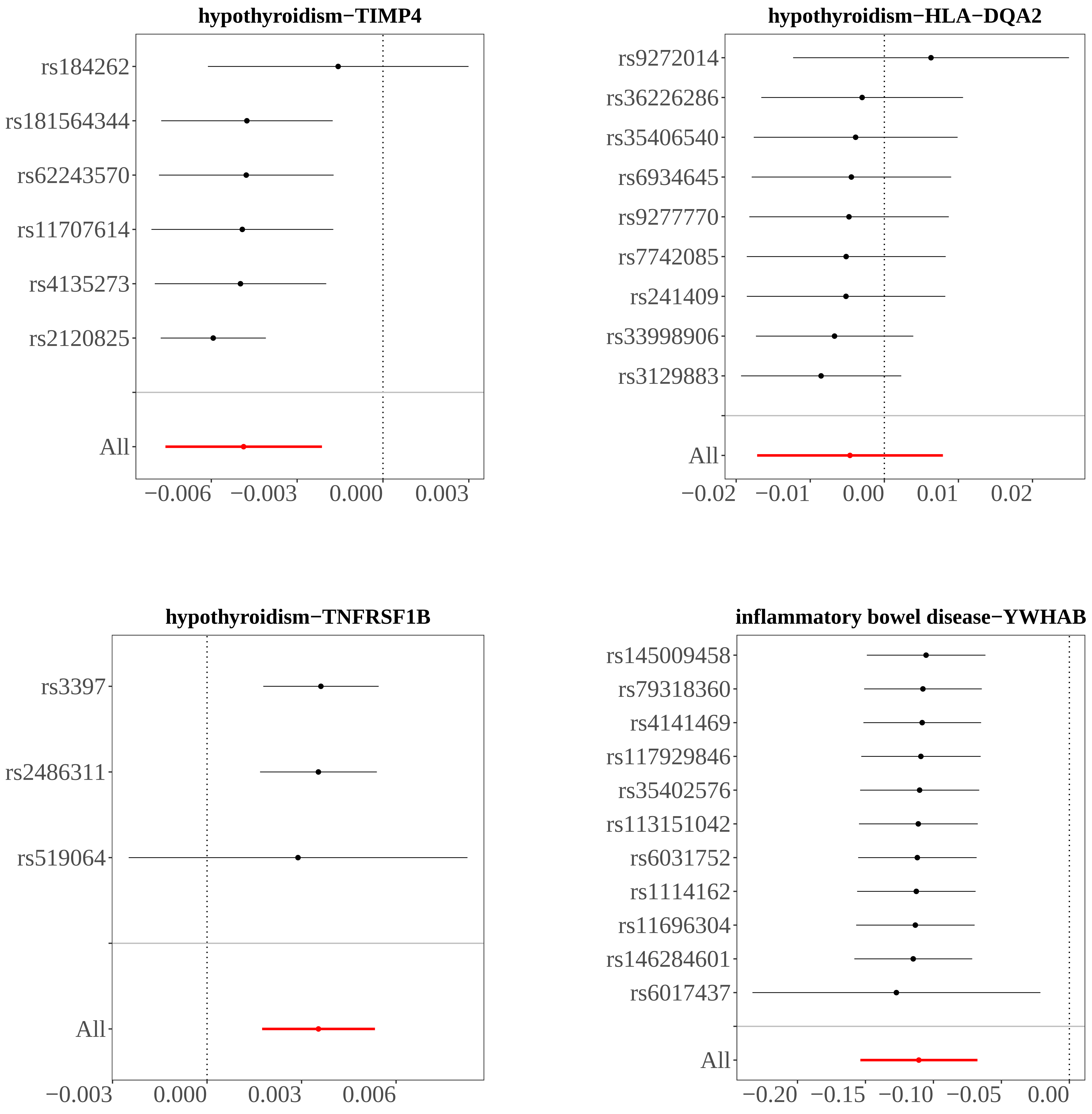
hypothyroidism−TIMP4
rs184262
rs181564344
rs62243570
rs11707614
rs4135273
rs2120825
All
−0.006
−0.003
0.000
0.003
hypothyroidism−HLA−DQA2
rs9272014
rs36226286
rs35406540
rs6934645
rs9277770
rs7742085
rs241409
rs33998906
rs3129883
All
−0.02
−0.01
0.00
0.01
0.02
hypothyroidism−TNFRSF1B
rs3397
rs2486311
rs519064
All
−0.003
0.000
0.003
0.006
inflammatory bowel disease−YWHAB
rs145009458
rs79318360
rs4141469
rs117929846
rs35402576
rs113151042
rs6031752
rs1114162
rs11696304
rs146284601
rs6017437
All
−0.20
−0.15
−0.10
−0.05
0.00

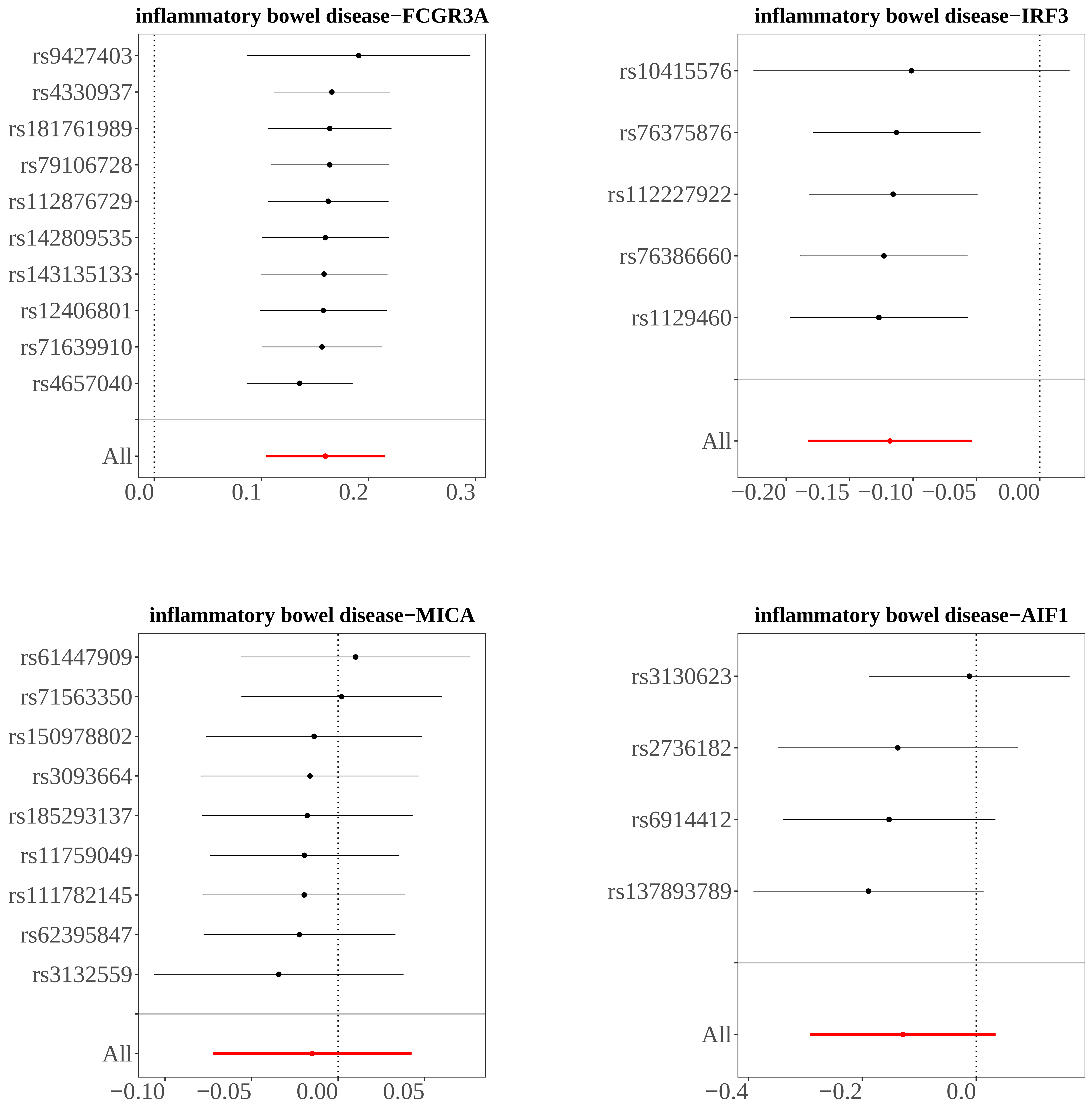

inflammatory bowel disease−FCGR3A
rs9427403
rs4330937
rs181761989
rs79106728
rs112876729
rs142809535
rs143135133
rs12406801
rs71639910
rs4657040
All
0.0
0.1
0.2
0.3
inflammatory bowel disease−IRF3
rs10415576
rs76375876
rs112227922
rs76386660
rs1129460
All
−0.20
−0.15
−0.10
−0.05
0.00
inflammatory bowel disease−MICA
rs61447909
rs71563350
rs150978802
rs3093664
rs185293137
rs11759049
rs111782145
rs62395847
rs3132559
All
−0.10
−0.05
0.00
0.05
inflammatory bowel disease−AIF1
rs3130623
rs2736182
rs6914412
rs137893789
All
−0.4
−0.2
0.0

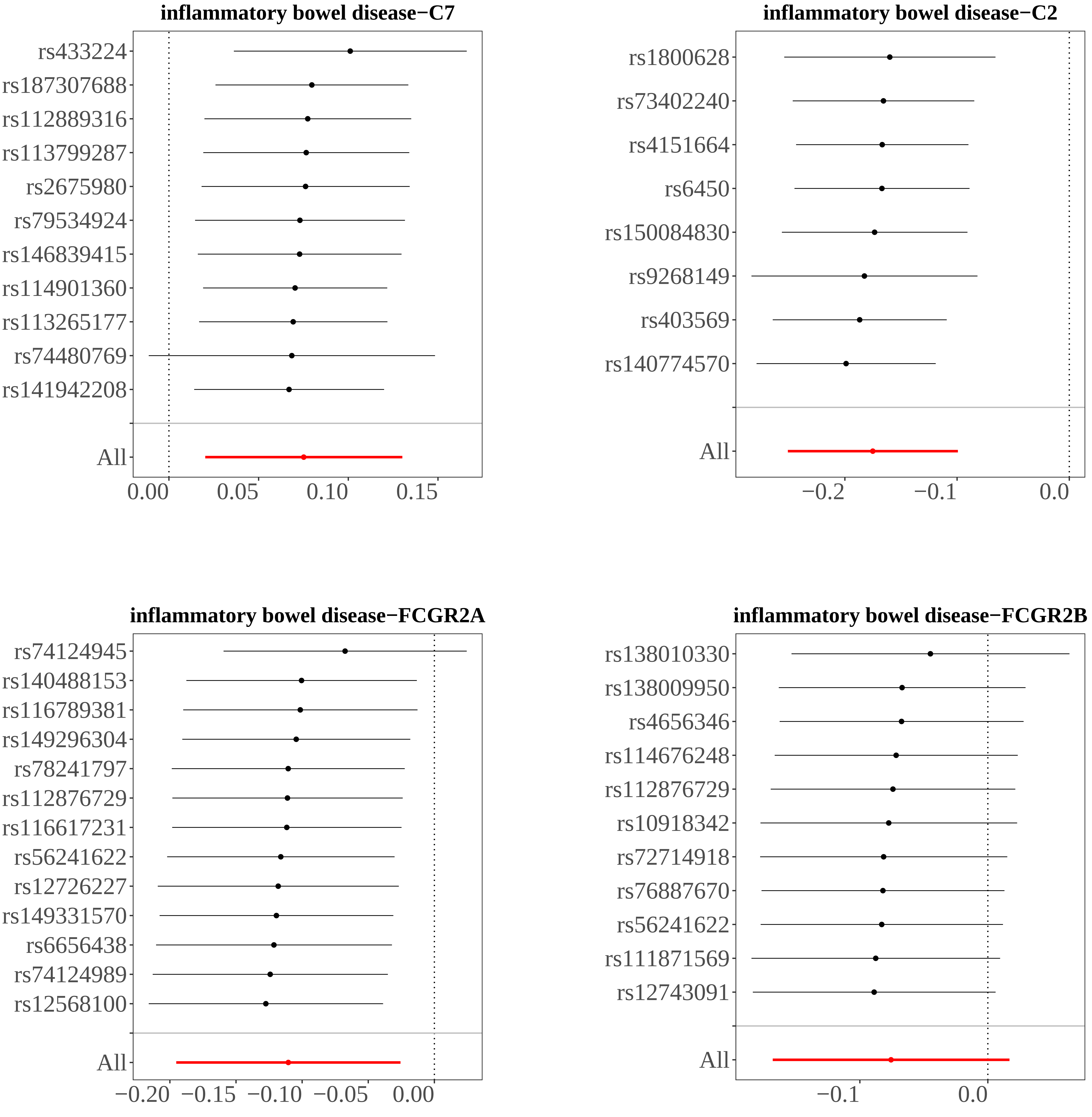
inflammatory bowel disease−C7
rs433224
rs187307688
rs112889316
rs113799287
rs2675980
rs79534924
rs146839415
rs114901360
rs113265177
rs74480769
rs141942208
All
0.00
0.05
0.10
0.15
inflammatory bowel disease−C2
rs1800628
rs73402240
rs4151664
rs6450
rs150084830
rs9268149
rs403569
rs140774570
All
−0.2
−0.1
0.0
inflammatory bowel disease−FCGR2A
rs74124945
rs140488153
rs116789381
rs149296304
rs78241797
rs112876729
rs116617231
rs56241622
rs12726227
rs149331570
rs6656438
rs74124989
rs12568100
All
−0.20
−0.15
−0.10
−0.05
0.00
inflammatory bowel disease−FCGR2B
rs138010330
rs138009950
rs4656346
rs114676248
rs112876729
rs10918342
rs72714918
rs76887670
rs56241622
rs111871569
rs12743091
All
−0.1
0.0

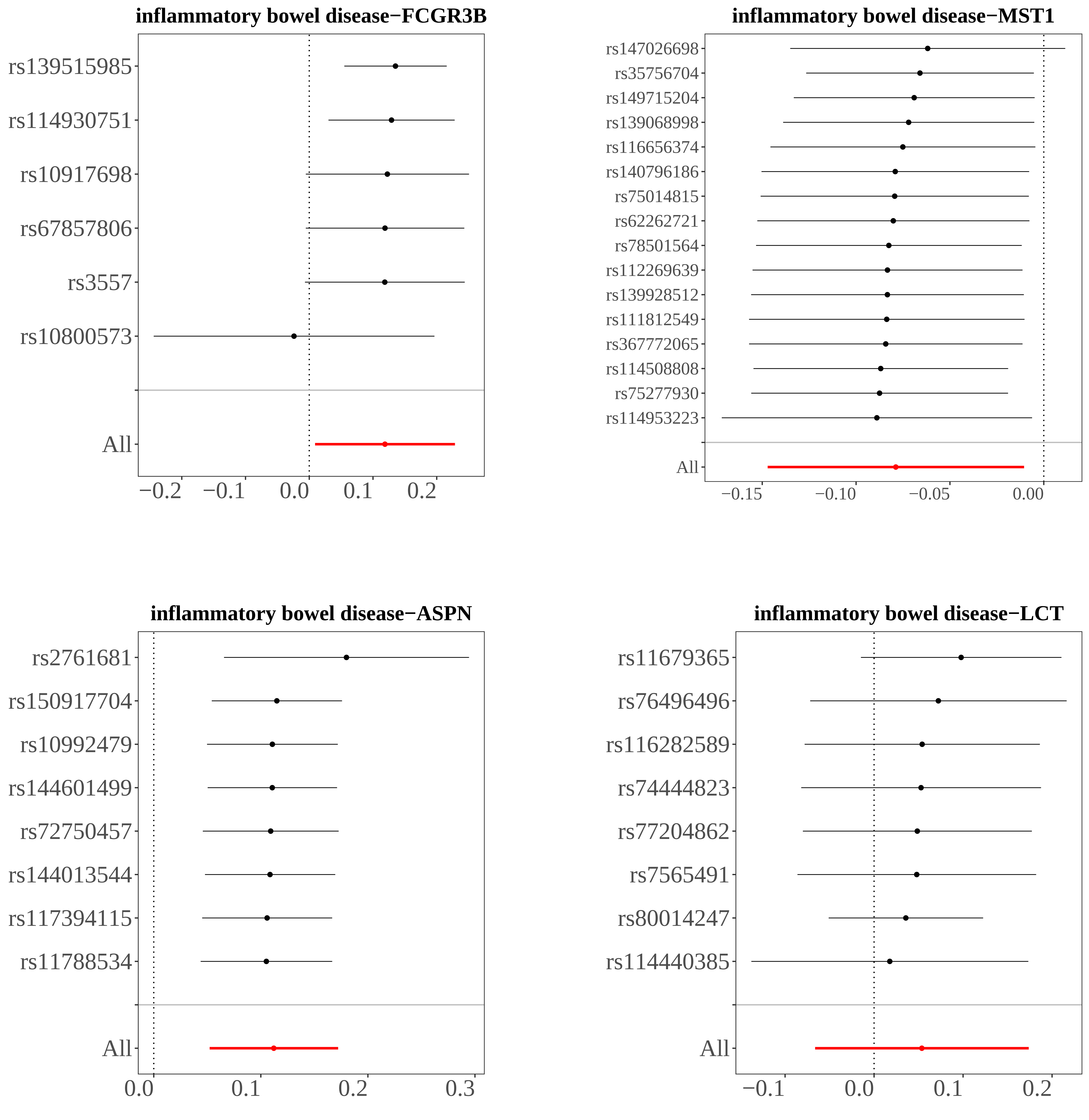
inflammatory bowel disease−FCGR3B
rs139515985
rs114930751
rs10917698
rs67857806
rs3557
rs10800573
All
−0.2
−0.1
0.0
0.1
0.2
inflammatory bowel disease−MST1
rs147026698
rs35756704
rs149715204
rs139068998
rs116656374
rs140796186
rs75014815
rs62262721
rs78501564
rs112269639
rs139928512
rs111812549
rs367772065
rs114508808
rs75277930
rs114953223
All
−0.15
−0.10
−0.05
0.00
inflammatory bowel disease−ASPN
rs2761681
rs150917704
rs10992479
rs144601499
rs72750457
rs144013544
rs117394115
rs11788534
All
0.0
0.1
0.2
0.3
inflammatory bowel disease−LCT
rs11679365
rs76496496
rs116282589
rs74444823
rs77204862
rs7565491
rs80014247
rs114440385
All
−0.1
0.0
0.1
0.2

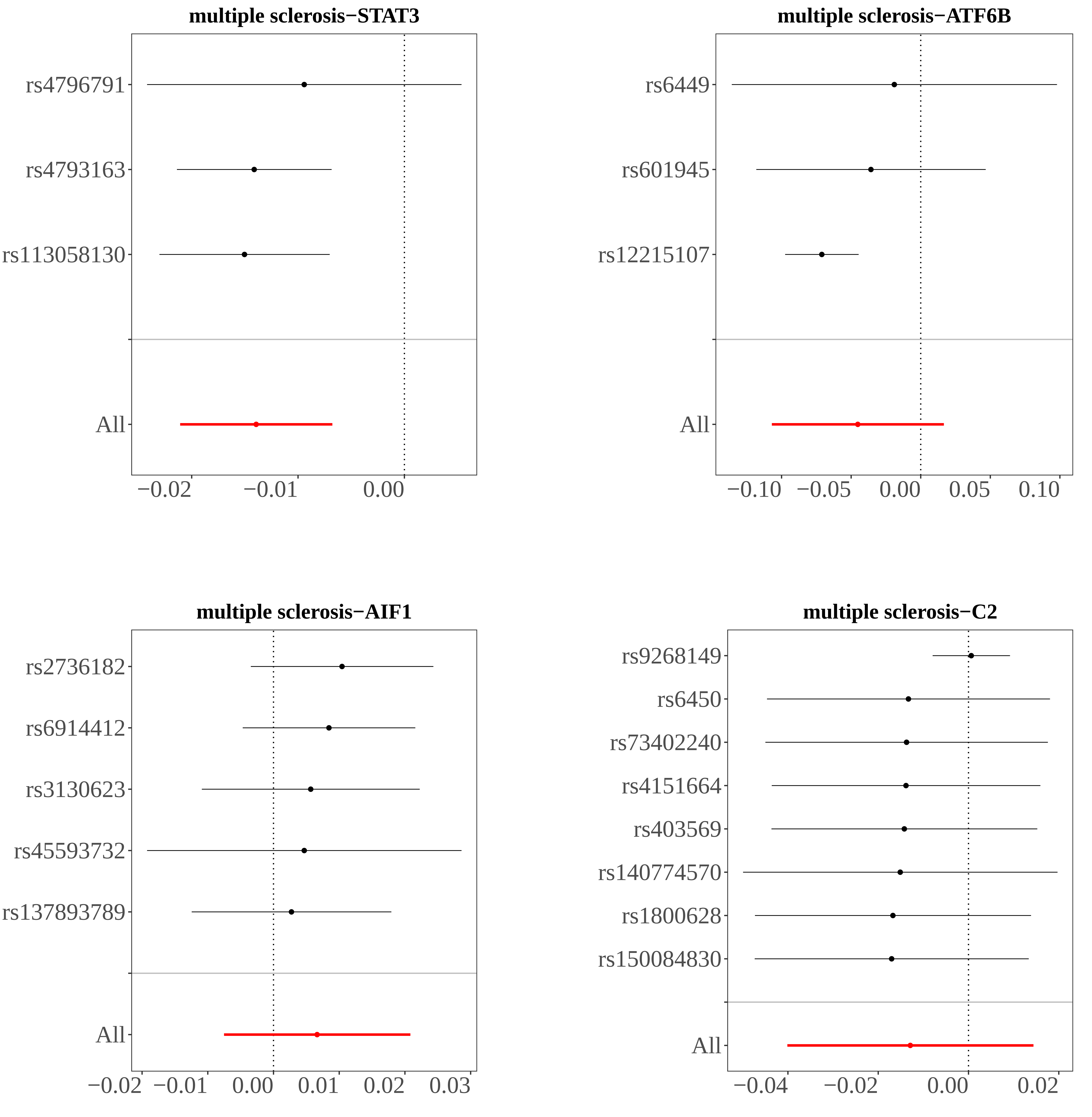
multiple sclerosis−STAT3
rs4796791
rs4793163
rs113058130
All
−0.02
−0.01
0.00
multiple sclerosis−ATF6B
rs6449
rs601945
rs12215107
All
−0.10
−0.05
0.00
0.05
0.10
multiple sclerosis−AIF1
rs2736182
rs6914412
rs3130623
rs45593732
rs137893789
All
−0.02
−0.01
0.00
0.01
0.02
0.03
multiple sclerosis−C2
rs9268149
rs6450
rs73402240
rs4151664
rs403569
rs140774570
rs1800628
rs150084830
All
−0.04
−0.02
0.00
0.02

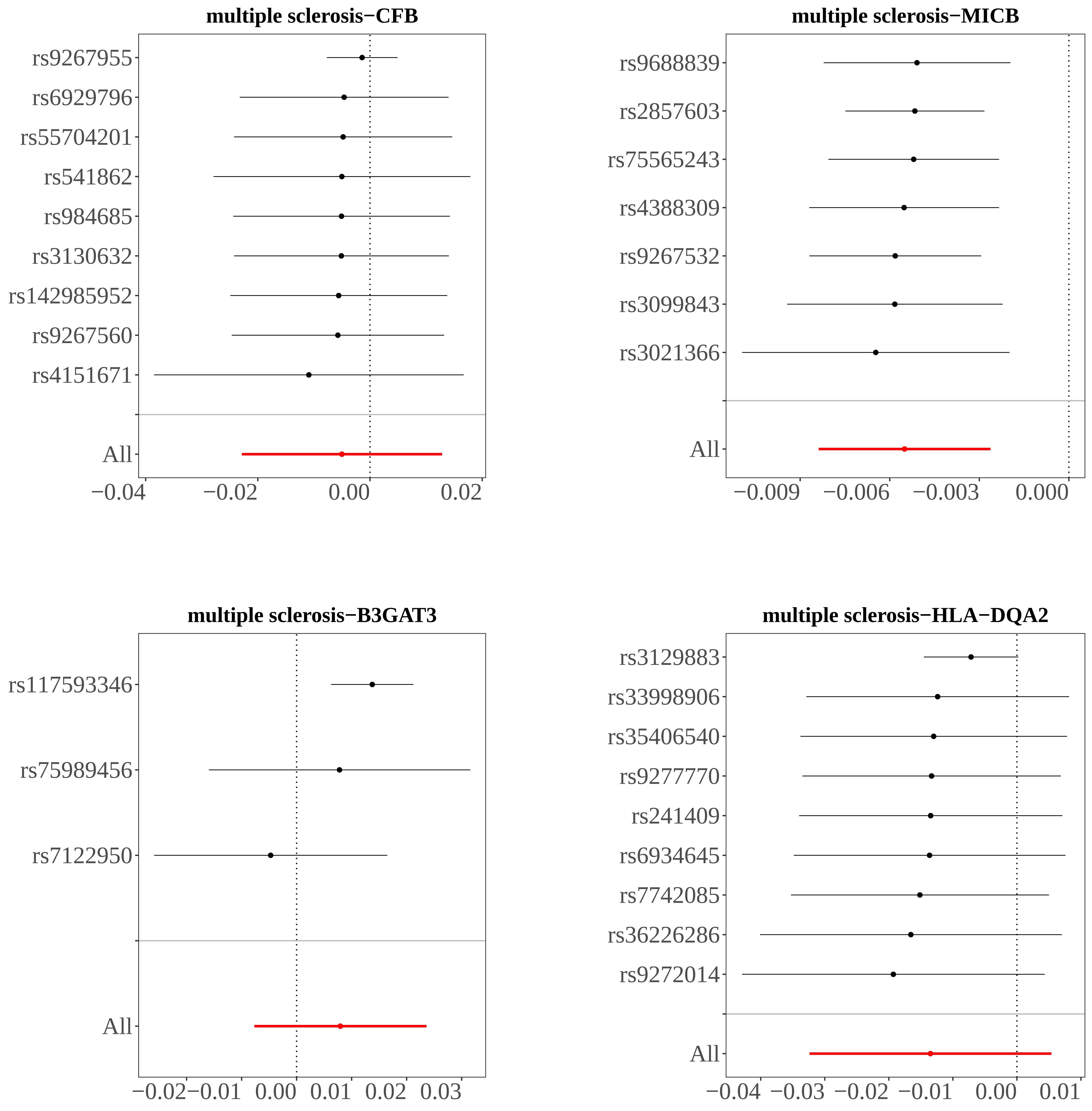

multiple sclerosis−CFB
rs9267955
rs6929796
rs55704201
rs541862
rs984685
rs3130632
rs142985952
rs9267560
rs4151671
All
−0.04
−0.02
0.00
0.02
multiple sclerosis−MICB
rs9688839
rs2857603
rs75565243
rs4388309
rs9267532
rs3099843
rs3021366
All
−0.009
−0.006
−0.003
0.000
multiple sclerosis−B3GAT3
rs117593346
rs75989456
rs7122950
All
−0.02
−0.01
0.00
0.01
0.02
0.03
multiple sclerosis−HLA−DQA2
rs3129883
rs33998906
rs35406540
rs9277770
rs241409
rs6934645
rs7742085
rs36226286
rs9272014
All
−0.04
−0.03
−0.02
−0.01
0.00
0.01

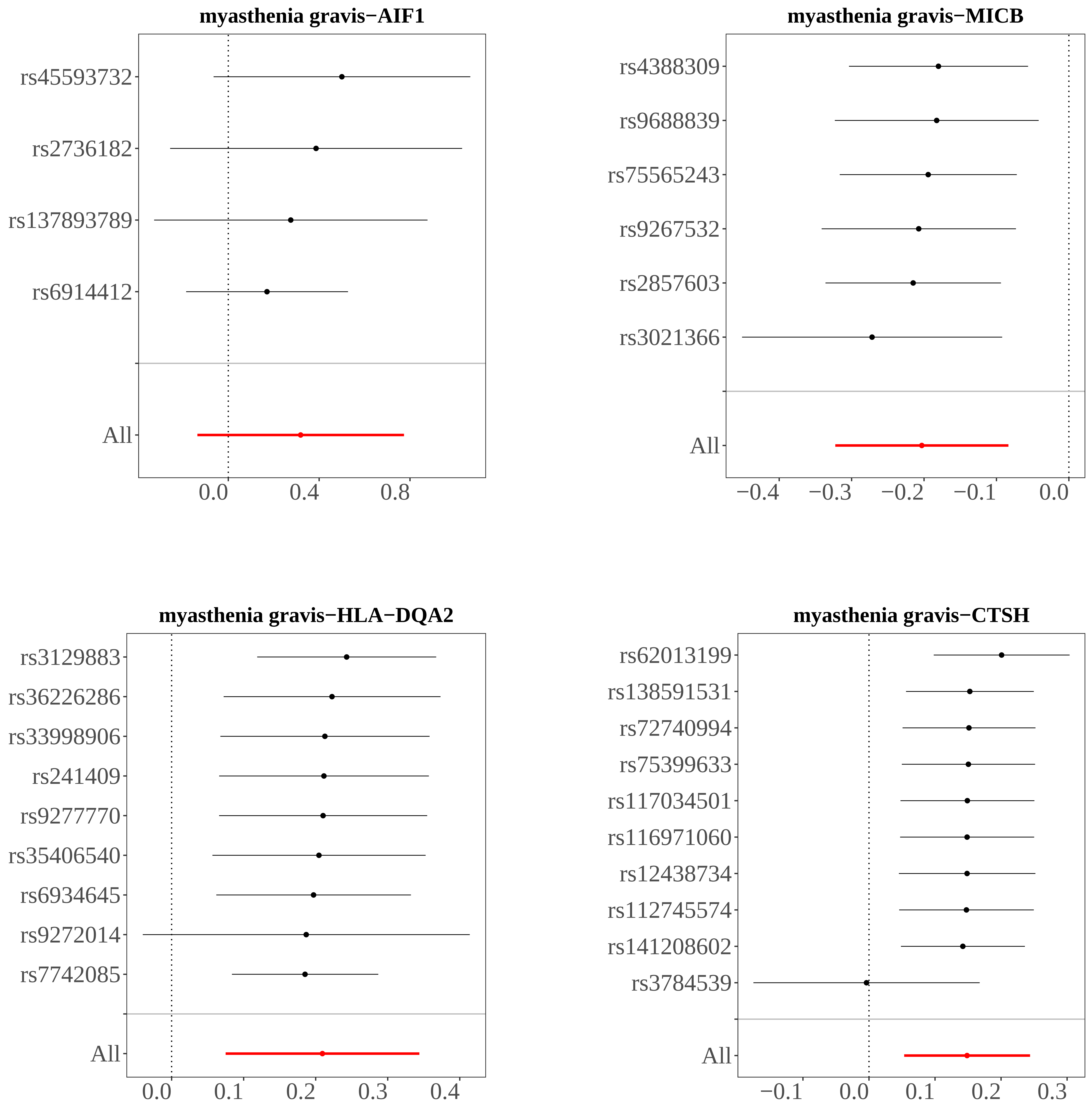
myasthenia gravis−AIF1
rs45593732
rs2736182
rs137893789
rs6914412
All
0.0
0.4
0.8
myasthenia gravis−MICB
rs4388309
rs9688839
rs75565243
rs9267532
rs2857603
rs3021366
All
−0.4
−0.3
−0.2
−0.1
0.0
myasthenia gravis−HLA−DQA2
rs3129883
rs36226286
rs33998906
rs241409
rs9277770
rs35406540
rs6934645
rs9272014
rs7742085
All
0.0
0.1
0.2
0.3
0.4
myasthenia gravis−CTSH
rs62013199
rs138591531
rs72740994
rs75399633
rs117034501
rs116971060
rs12438734
rs112745574
rs141208602
rs3784539
All
−0.1
0.0
0.1
0.2
0.3

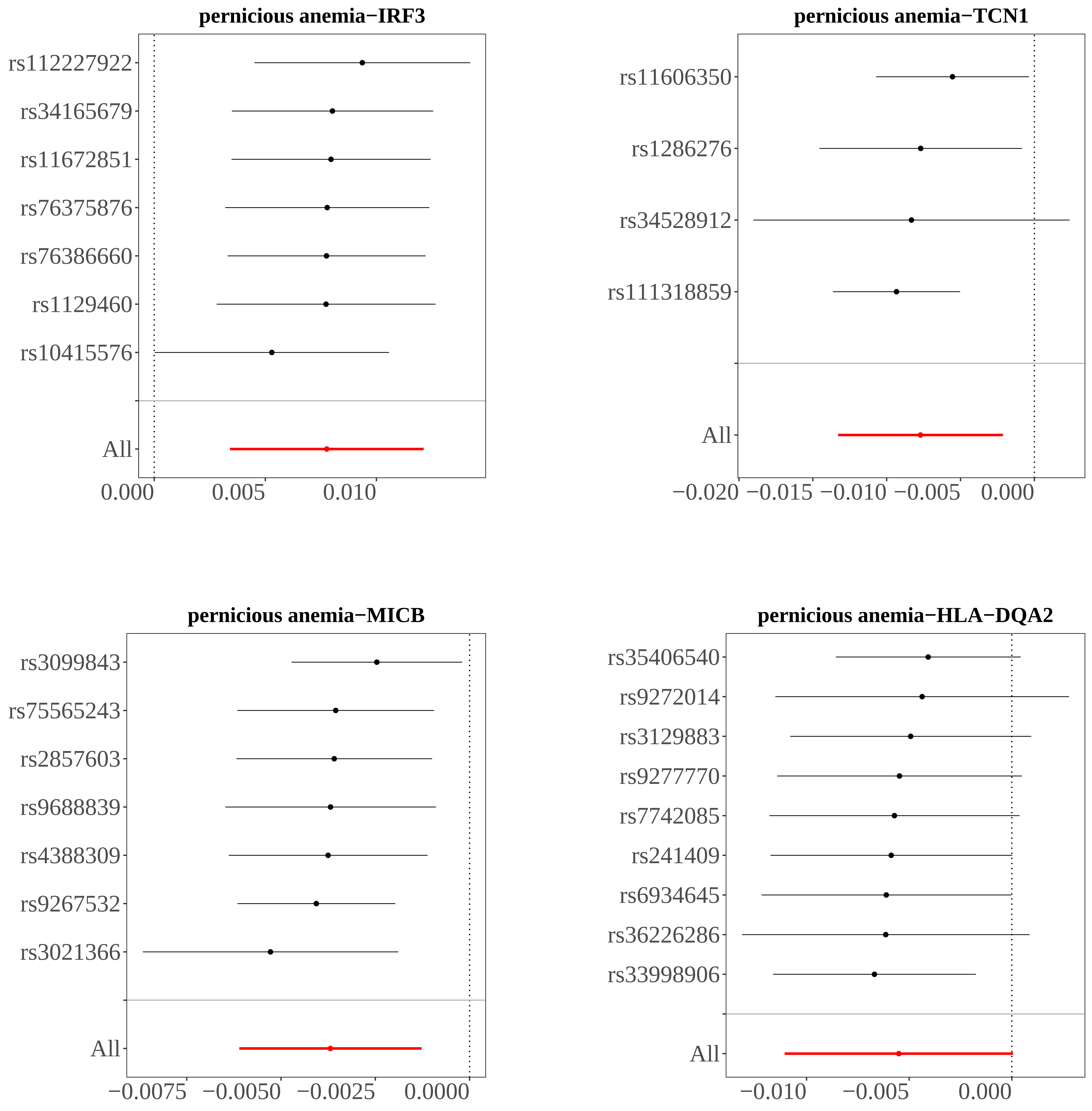
pernicious anemia−IRF3
rs112227922
rs34165679
rs11672851
rs76375876
rs76386660
rs1129460
rs10415576
All
0.000
0.005
0.010
pernicious anemia−TCN1
rs11606350
rs1286276
rs34528912
rs111318859
All
−0.020
−0.015
−0.010
−0.005
0.000
pernicious anemia−MICB
rs3099843
rs75565243
rs2857603
rs9688839
rs4388309
rs9267532
rs3021366
All
−0.0075
−0.0050
−0.0025
0.0000
pernicious anemia−HLA−DQA2
rs35406540
rs9272014
rs3129883
rs9277770
rs7742085
rs241409
rs6934645
rs36226286
rs33998906
All
−0.010
−0.005
0.000

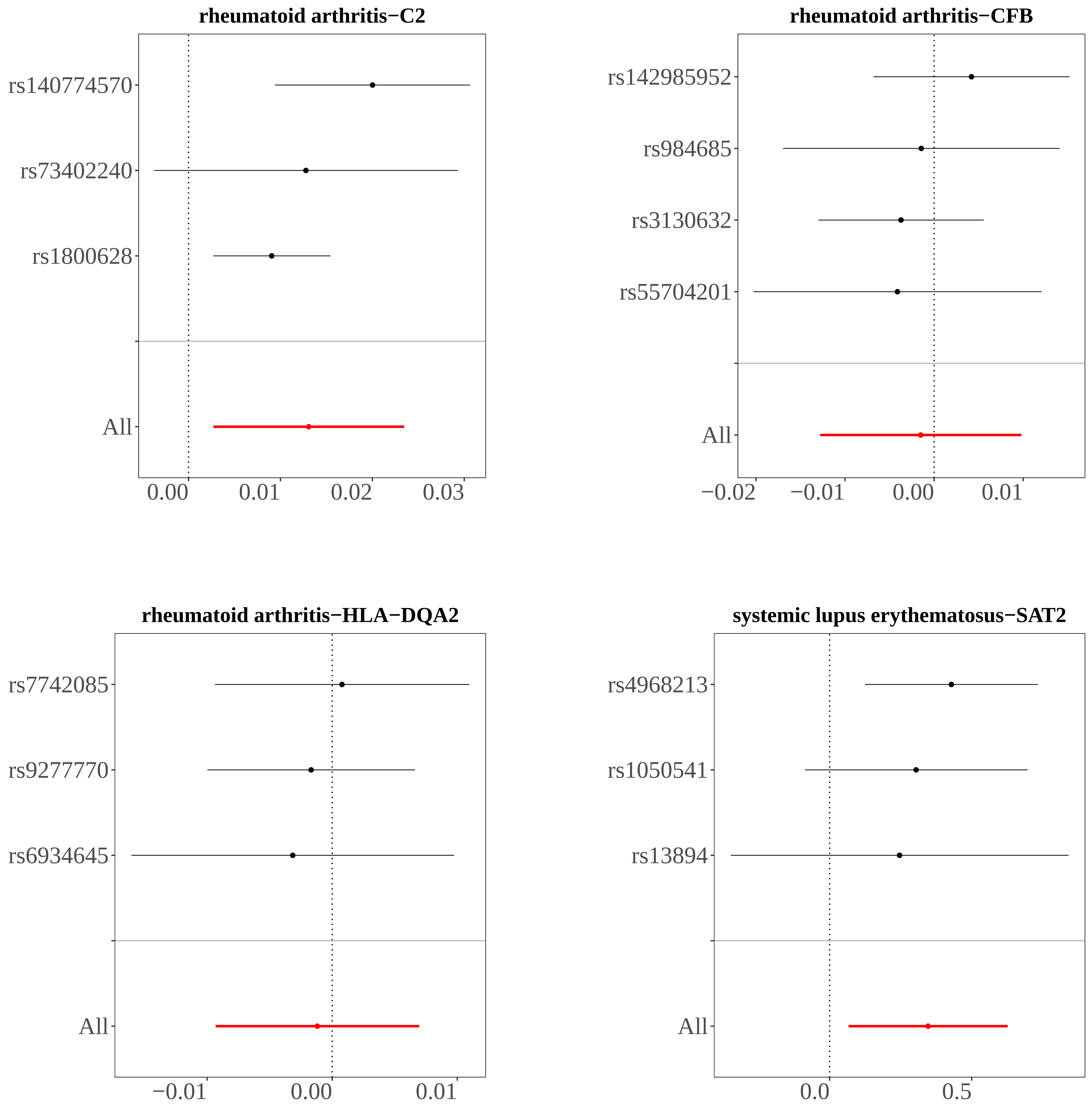

rheumatoid arthritis−C2
rs140774570
rs73402240
rs1800628
All
0.00
0.01
0.02
0.03
rheumatoid arthritis−CFB
rs142985952
rs984685
rs3130632
rs55704201
All
−0.02
−0.01
0.00
0.01
rheumatoid arthritis−HLA−DQA2
rs7742085
rs9277770
rs6934645
All
−0.01
0.00
0.01
systemic lupus erythematosus−SAT2
rs4968213
rs1050541
rs13894
All
0.0
0.5

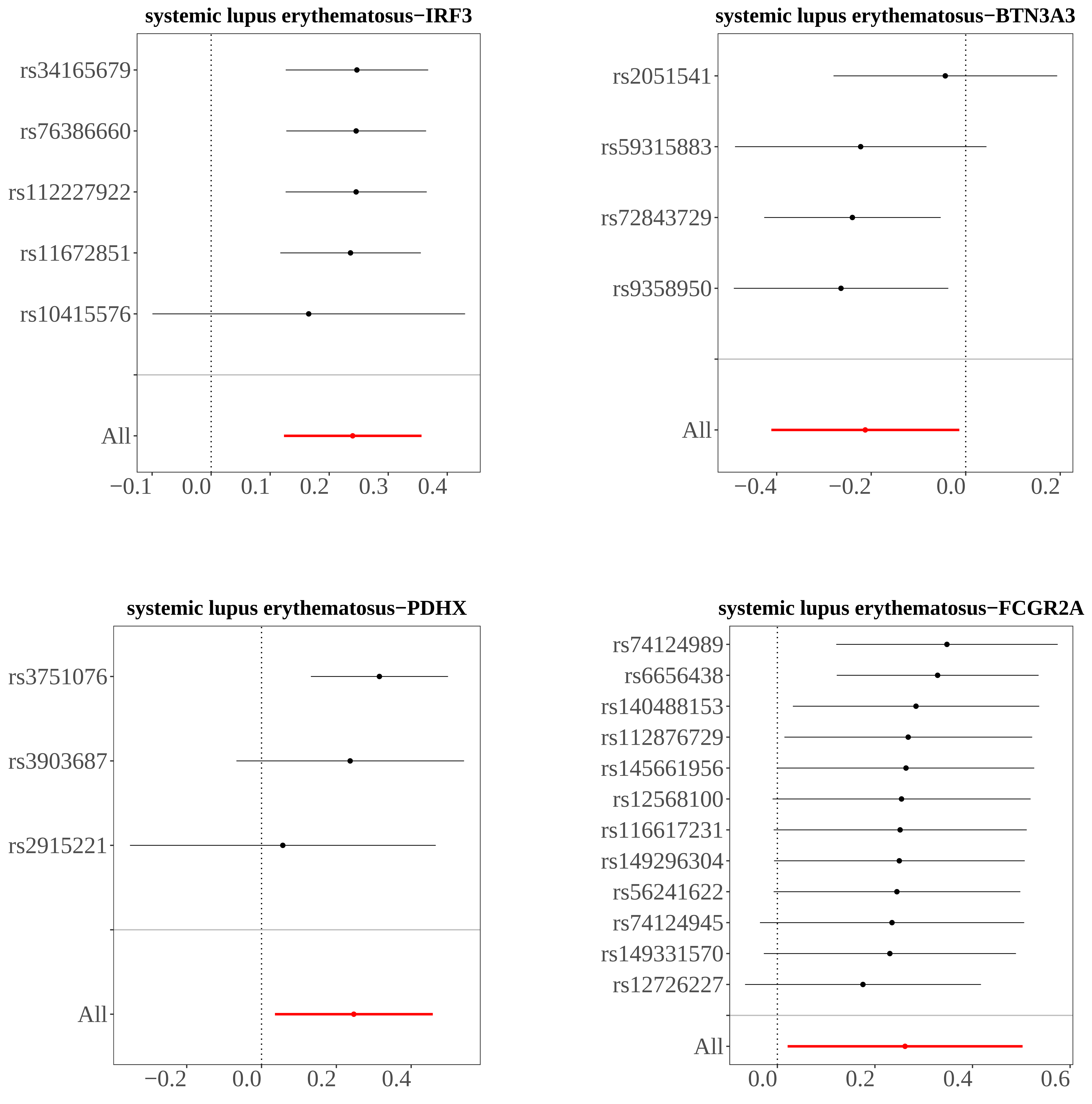

systemic lupus erythematosus−IRF3
rs34165679
rs76386660
rs112227922
rs11672851
rs10415576
All
−0.1
0.0
0.1
0.2
0.3
0.4
systemic lupus erythematosus−BTN3A3
rs2051541
rs59315883
rs72843729
rs9358950
All
−0.4
−0.2
0.0
0.2
systemic lupus erythematosus−PDHX
rs3751076
rs3903687
rs2915221
All
−0.2
0.0
0.2
0.4
systemic lupus erythematosus−FCGR2A
rs74124989
rs6656438
rs140488153
rs112876729
rs145661956
rs12568100
rs116617231
rs149296304
rs56241622
rs74124945
rs149331570
rs12726227
All
0.0
0.2
0.4
0.6

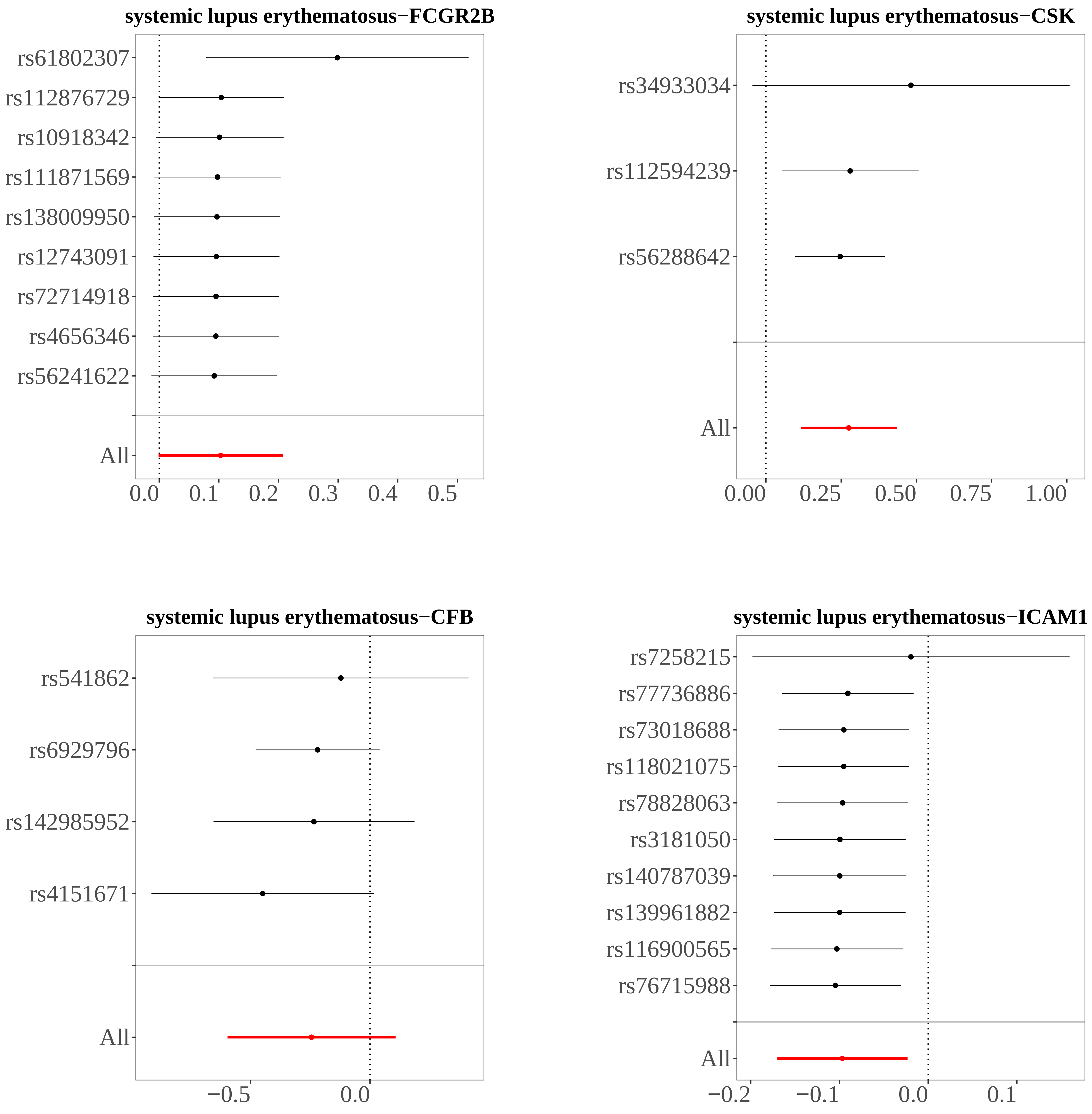
systemic lupus erythematosus−FCGR2B
rs61802307
rs112876729
rs10918342
rs111871569
rs138009950
rs12743091
rs72714918
rs4656346
rs56241622
All
0.0
0.1
0.2
0.3
0.4
0.5
systemic lupus erythematosus−CSK
rs34933034
rs112594239
rs56288642
All
0.00
0.25
0.50
0.75
1.00
systemic lupus erythematosus−CFB
rs541862
rs6929796
rs142985952
rs4151671
All
−0.5
0.0
systemic lupus erythematosus−ICAM1
rs7258215
rs77736886
rs73018688
rs118021075
rs78828063
rs3181050
rs140787039
rs139961882
rs116900565
rs76715988
All
−0.2
−0.1
0.0
0.1

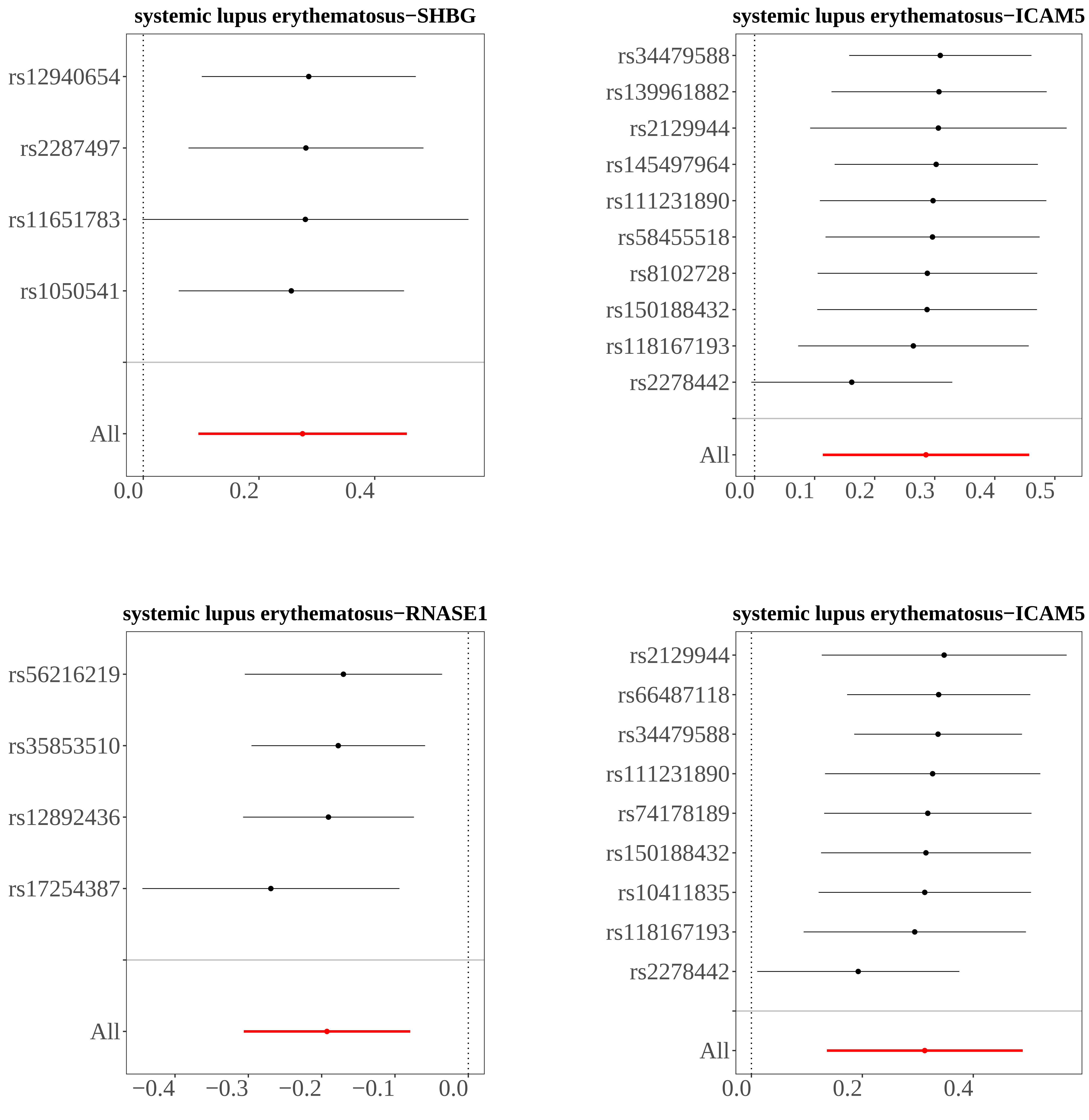
systemic lupus erythematosus−SHBG
rs12940654
rs2287497
rs11651783
rs1050541
All
0.0
0.2
0.4
systemic lupus erythematosus−ICAM5
rs34479588
rs139961882
rs2129944
rs145497964
rs111231890
rs58455518
rs8102728
rs150188432
rs118167193
rs2278442
All
0.0
0.1
0.2
0.3
0.4
0.5
systemic lupus erythematosus−RNASE1
rs56216219
rs35853510
rs12892436
rs17254387
All
−0.4
−0.3
−0.2
−0.1
0.0
systemic lupus erythematosus−ICAM5
rs2129944
rs66487118
rs34479588
rs111231890
rs74178189
rs150188432
rs10411835
rs118167193
rs2278442
All
0.0
0.2
0.4

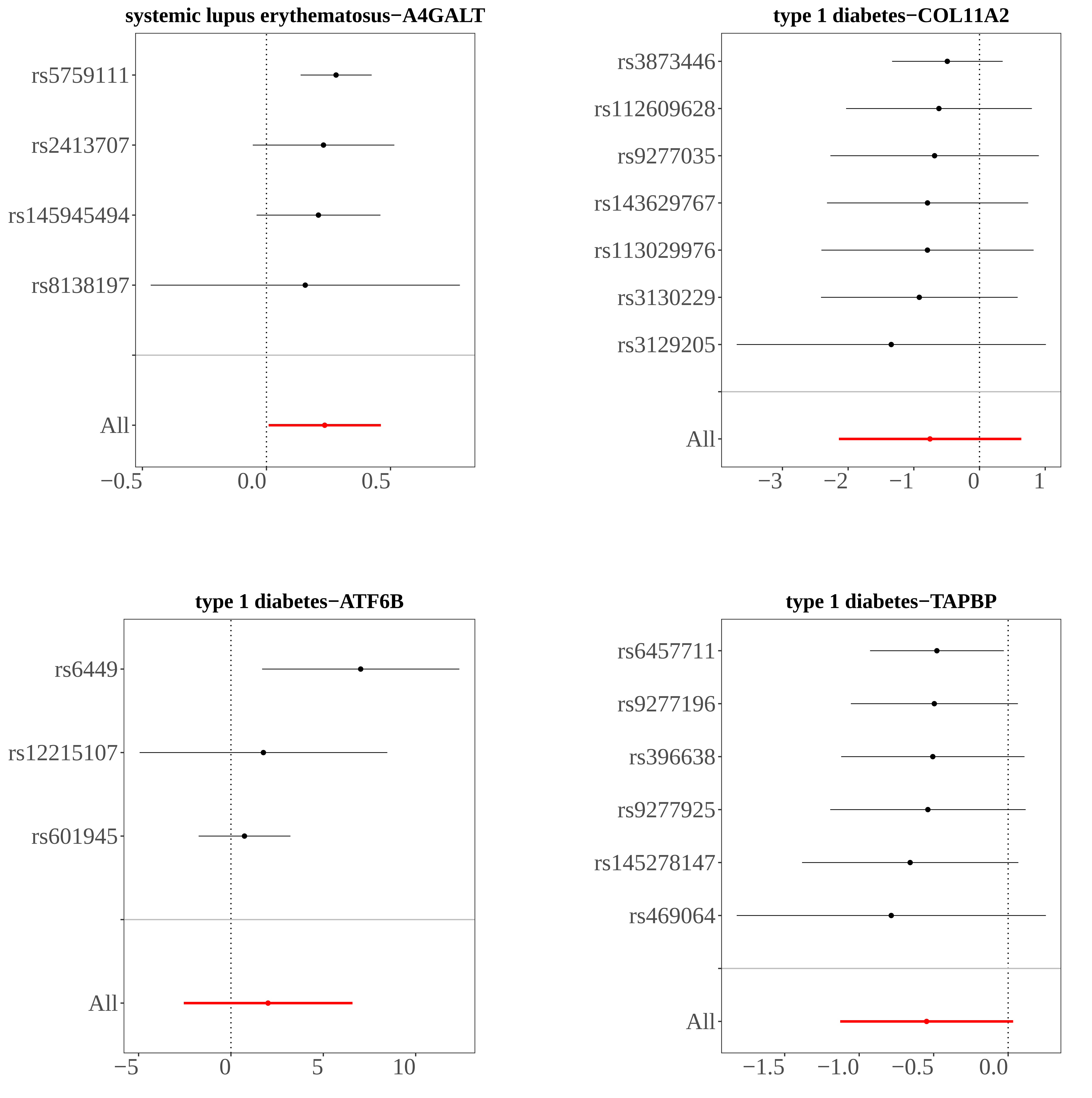

systemic lupus erythematosus−A4GALT
rs5759111
rs2413707
rs145945494
rs8138197
All
−0.5
0.0
0.5
type 1 diabetes−COL11A2
rs3873446
rs112609628
rs9277035
rs143629767
rs113029976
rs3130229
rs3129205
All
−3
−2
−1
0
1
type 1 diabetes−ATF6B
rs6449
rs12215107
rs601945
All
−5
0
5
10
type 1 diabetes−TAPBP
rs6457711
rs9277196
rs396638
rs9277925
rs145278147
rs469064
All
−1.5
−1.0
−0.5
0.0

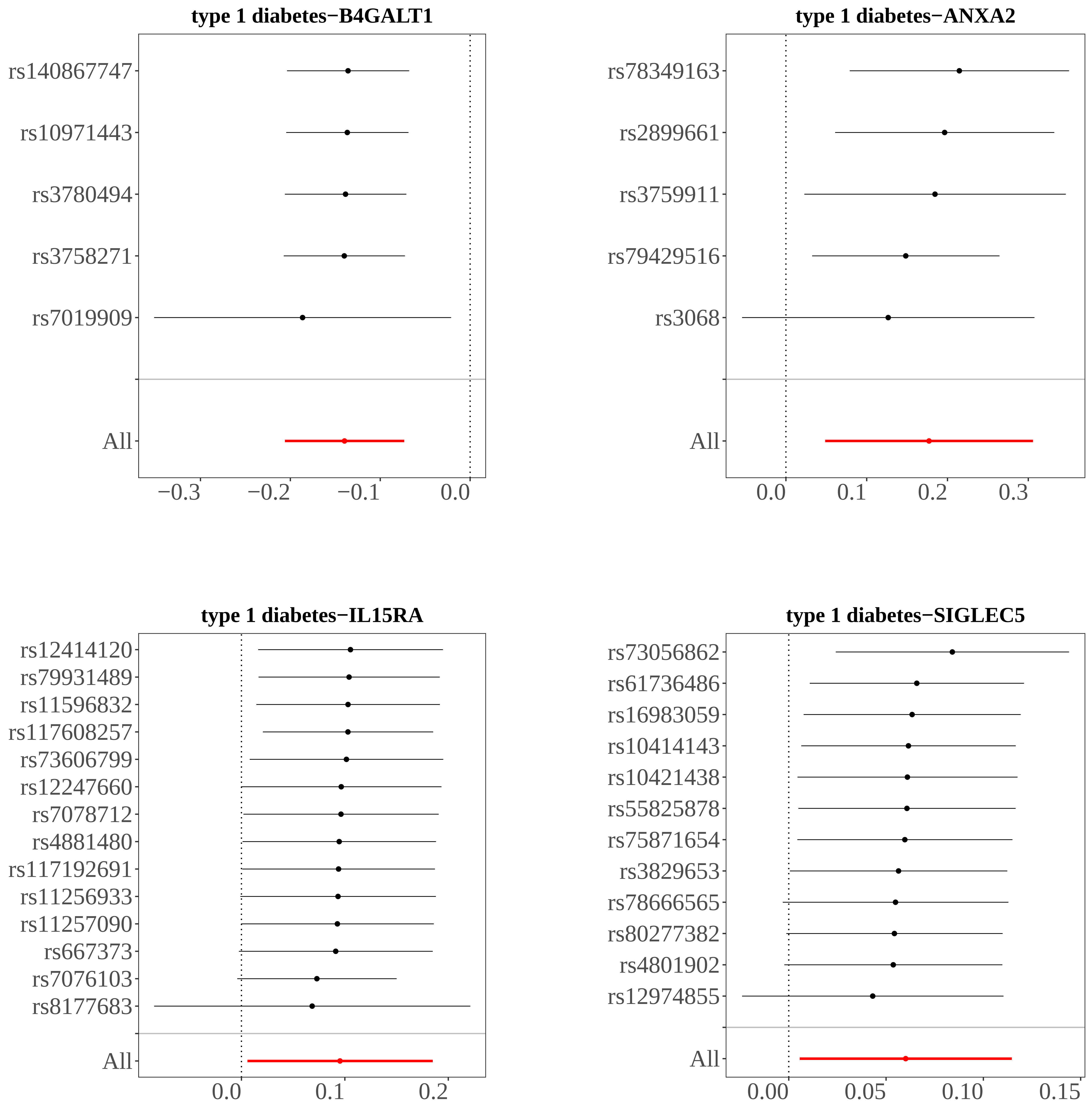
type 1 diabetes−B4GALT1
rs140867747
rs10971443
rs3780494
rs3758271
rs7019909
All
−0.3
−0.2
−0.1
0.0
type 1 diabetes−ANXA2
rs78349163
rs2899661
rs3759911
rs79429516
rs3068
All
0.0
0.1
0.2
0.3
type 1 diabetes−IL15RA
rs12414120
rs79931489
rs11596832
rs117608257
rs73606799
rs12247660
rs7078712
rs4881480
rs117192691
rs11256933
rs11257090
rs667373
rs7076103
rs8177683
All
0.0
0.1
0.2
type 1 diabetes−SIGLEC5
rs73056862
rs61736486
rs16983059
rs10414143
rs10421438
rs55825878
rs75871654
rs3829653
rs78666565
rs80277382
rs4801902
rs12974855
All
0.00
0.05
0.10
0.15

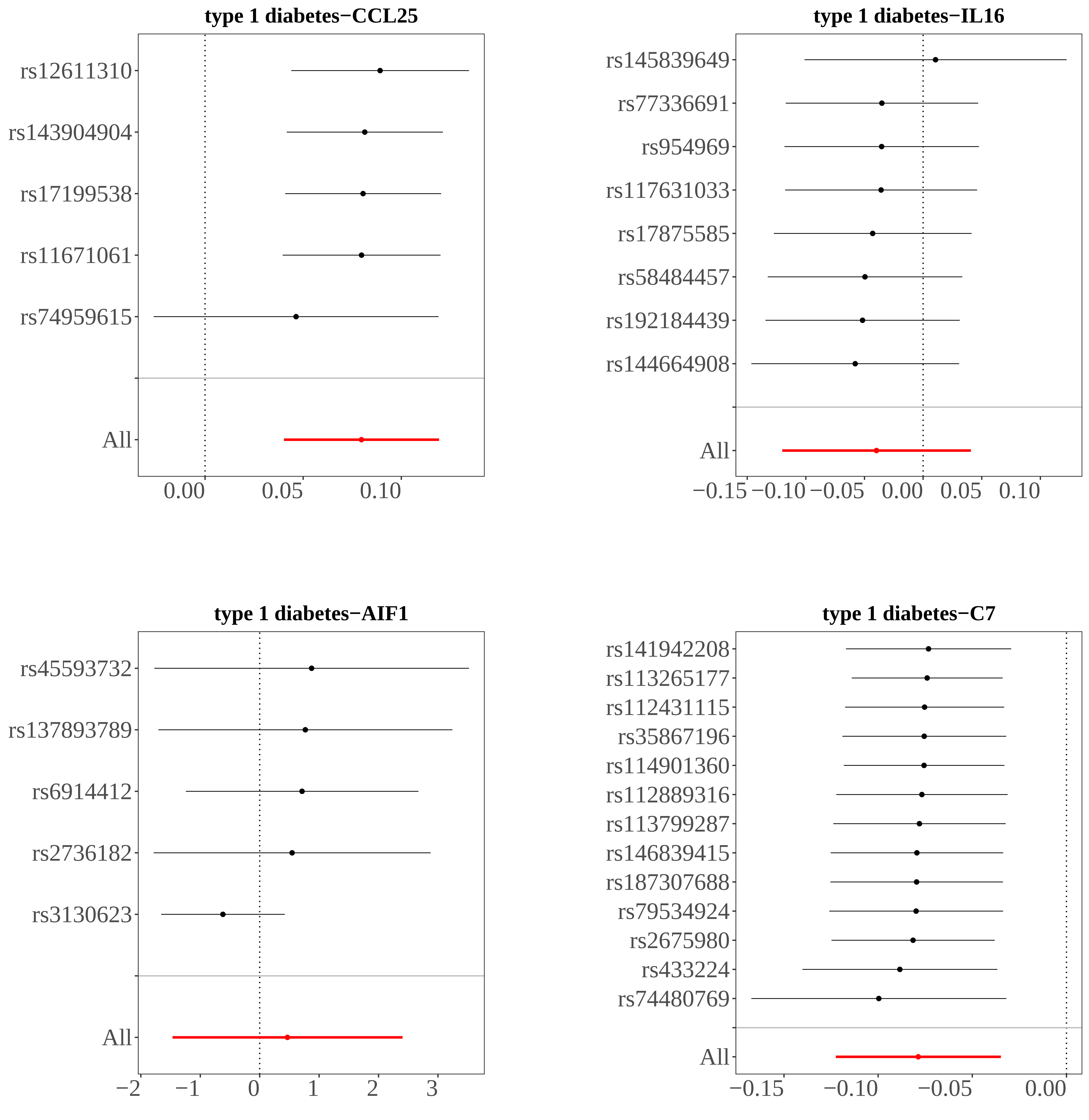
type 1 diabetes−CCL25
rs12611310
rs143904904
rs17199538
rs11671061
rs74959615
All
0.00
0.05
0.10
type 1 diabetes−IL16
rs145839649
rs77336691
rs954969
rs117631033
rs17875585
rs58484457
rs192184439
rs144664908
All
−0.15
−0.10
−0.05
0.00
0.05
0.10
type 1 diabetes−AIF1
rs45593732
rs137893789
rs6914412
rs2736182
rs3130623
All
−2
−1
0
1
2
3
type 1 diabetes−C7
rs141942208
rs113265177
rs112431115
rs35867196
rs114901360
rs112889316
rs113799287
rs146839415
rs187307688
rs79534924
rs2675980
rs433224
rs74480769
All
−0.15
−0.10
−0.05
0.00

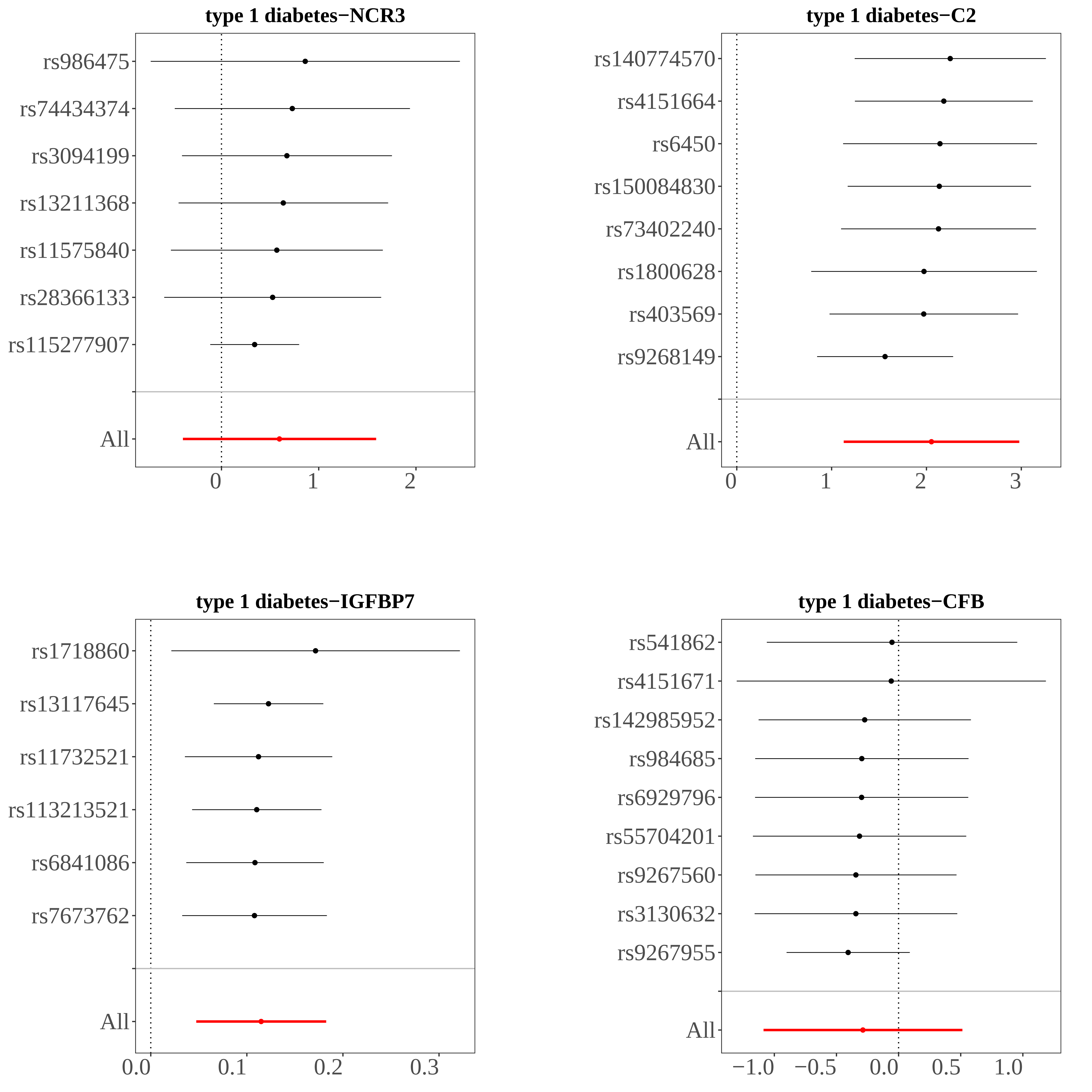

type 1 diabetes−NCR3
rs986475
rs74434374
rs3094199
rs13211368
rs11575840
rs28366133
rs115277907
All
0
1
2
type 1 diabetes−C2
rs140774570
rs4151664
rs6450
rs150084830
rs73402240
rs1800628
rs403569
rs9268149
All
0
1
2
3
type 1 diabetes−IGFBP7
rs1718860
rs13117645
rs11732521
rs113213521
rs6841086
rs7673762
All
0.0
0.1
0.2
0.3
type 1 diabetes−CFB
rs541862
rs4151671
rs142985952
rs984685
rs6929796
rs55704201
rs9267560
rs3130632
rs9267955
All
−1.0
−0.5
0.0
0.5
1.0

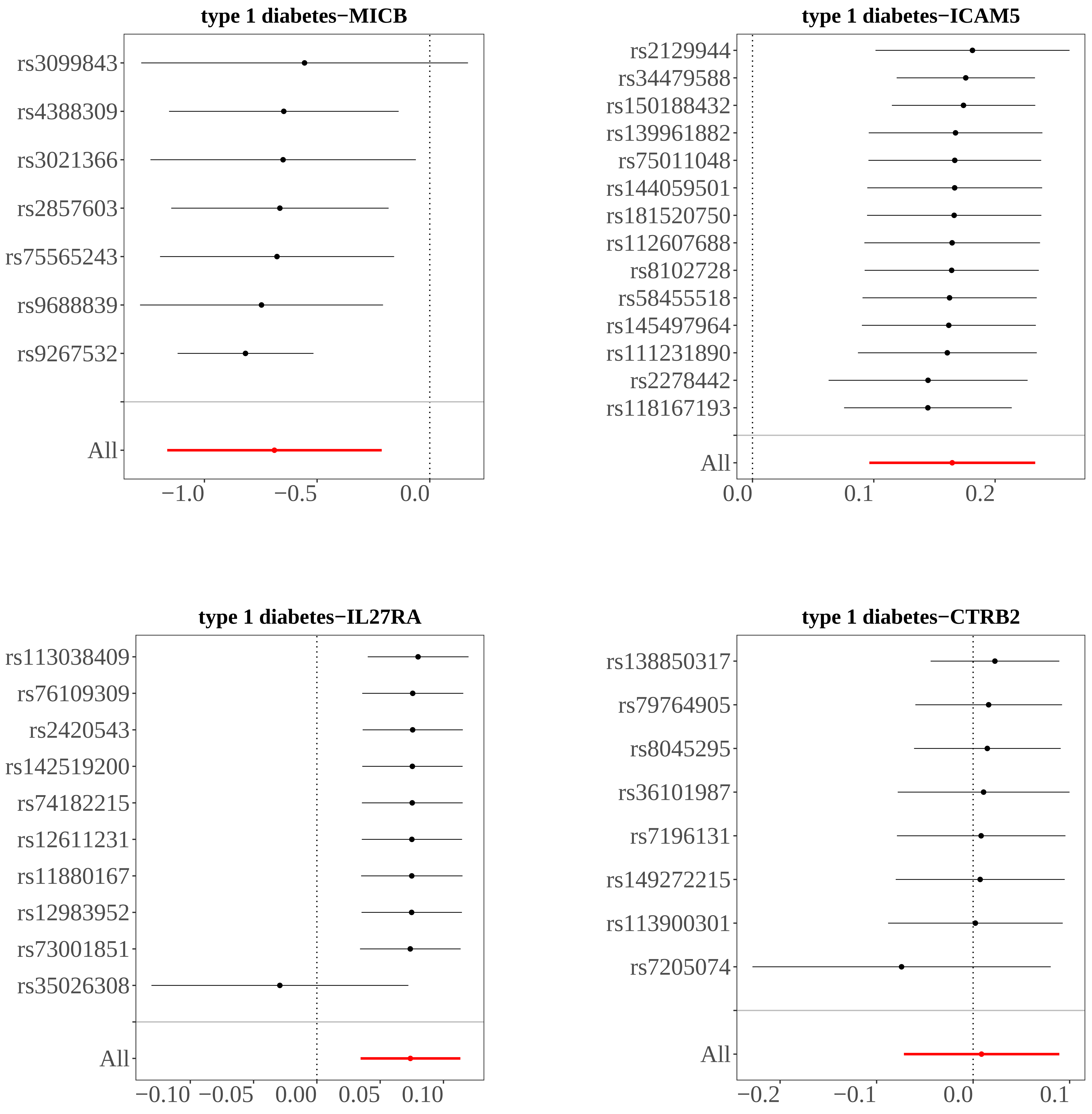
type 1 diabetes−MICB
rs3099843
rs4388309
rs3021366
rs2857603
rs75565243
rs9688839
rs9267532
All
−1.0
−0.5
0.0
type 1 diabetes−ICAM5
rs2129944
rs34479588
rs150188432
rs139961882
rs75011048
rs144059501
rs181520750
rs112607688
rs8102728
rs58455518
rs145497964
rs111231890
rs2278442
rs118167193
All
0.0
0.1
0.2
type 1 diabetes−IL27RA
rs113038409
rs76109309
rs2420543
rs142519200
rs74182215
rs12611231
rs11880167
rs12983952
rs73001851
rs35026308
All
−0.10
−0.05
0.00
0.05
0.10
type 1 diabetes−CTRB2
rs138850317
rs79764905
rs8045295
rs36101987
rs7196131
rs149272215
rs113900301
rs7205074
All
−0.2
−0.1
0.0
0.1

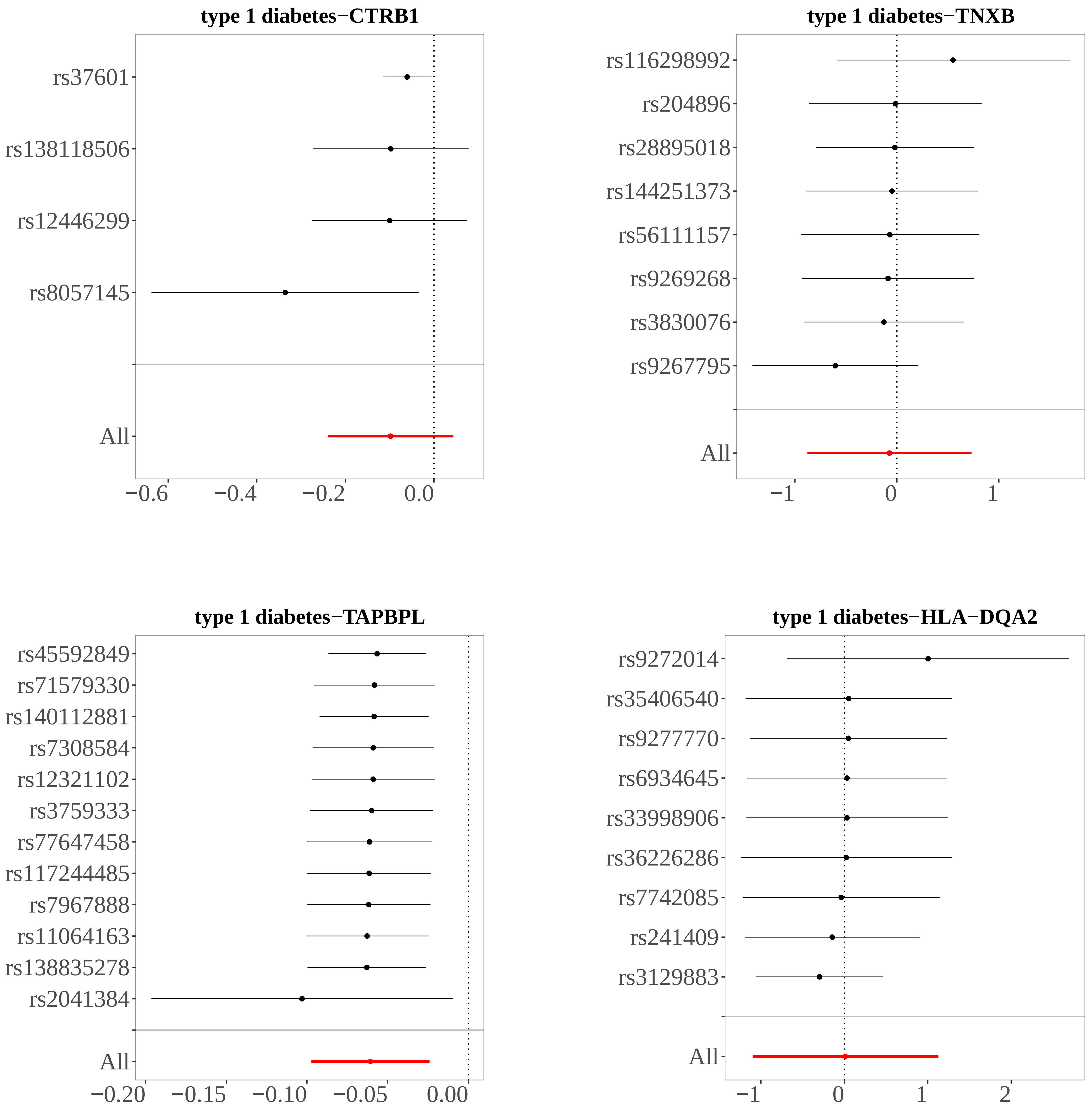
type 1 diabetes−CTRB1
rs37601
rs138118506
rs12446299
rs8057145
All
−0.6
−0.4
−0.2
0.0
type 1 diabetes−TNXB
rs116298992
rs204896
rs28895018
rs144251373
rs56111157
rs9269268
rs3830076
rs9267795
All
−1
0
1
type 1 diabetes−TAPBPL
rs45592849
rs71579330
rs140112881
rs7308584
rs12321102
rs3759333
rs77647458
rs117244485
rs7967888
rs11064163
rs138835278
rs2041384
All
−0.20
−0.15
−0.10
−0.05
0.00
type 1 diabetes−HLA−DQA2
rs9272014
rs35406540
rs9277770
rs6934645
rs33998906
rs36226286
rs7742085
rs241409
rs3129883
All
−1
0
1
2

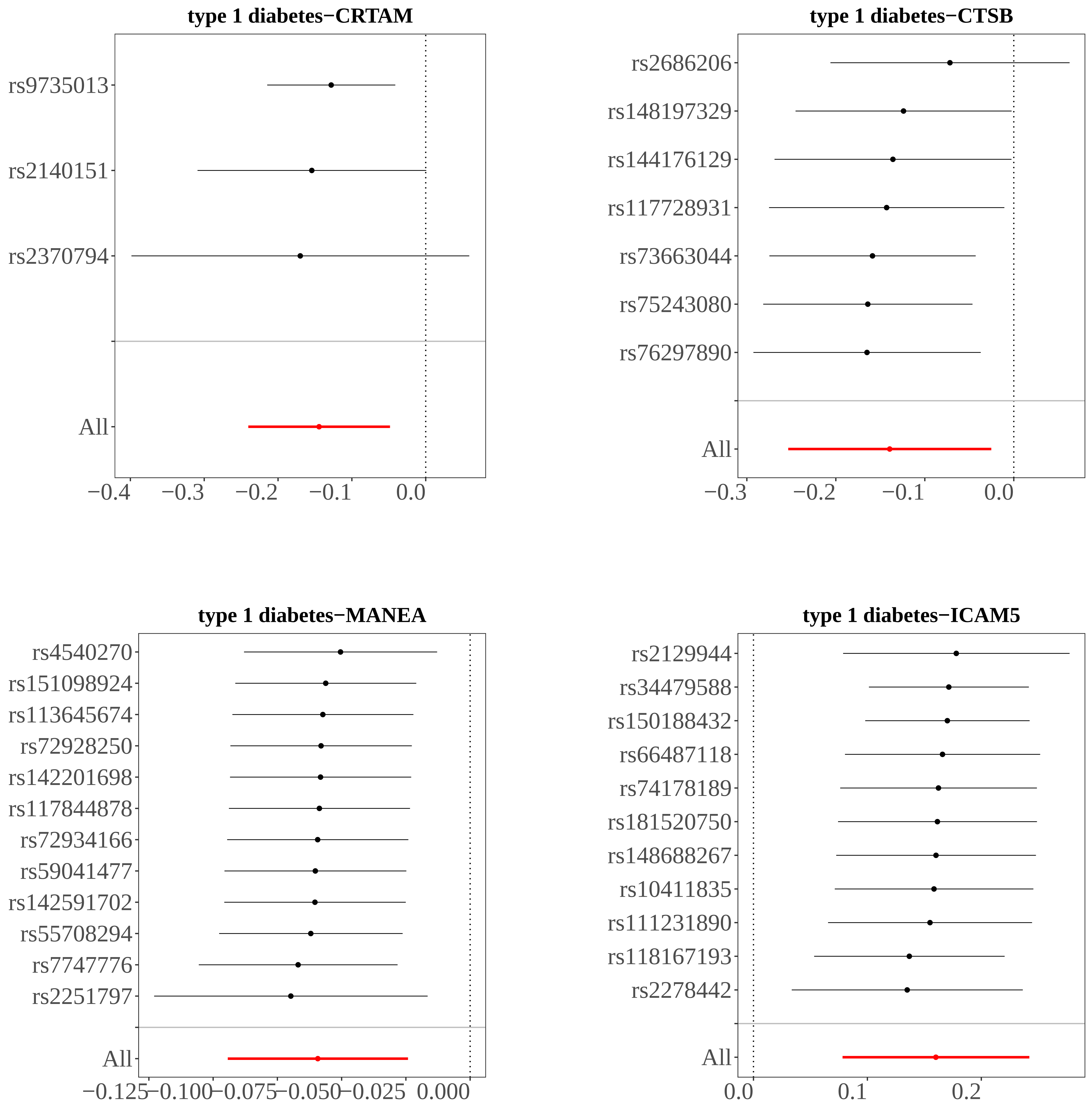

type 1 diabetes−CRTAM
rs9735013
rs2140151
rs2370794
All
−0.4 −0.3 −0.2 −0.1 0.0
type 1 diabetes−CTSB
rs2686206
rs148197329
rs144176129
rs117728931
rs73663044
rs75243080
rs76297890
All
−0.3 −0.2 −0.1 0.0
type 1 diabetes−MANEA
rs4540270
rs151098924
rs113645674
rs72928250
rs142201698
rs117844878
rs72934166
rs59041477
rs142591702
rs55708294
rs7747776
rs2251797
All
−0.125 −0.100 −0.075 −0.050 −0.025 0.000
type 1 diabetes−ICAM5
rs2129944
rs34479588
rs150188432
rs66487118
rs74178189
rs181520750
rs148688267
rs10411835
rs111231890
rs118167193
rs2278442
All
0.0 0.1 0.2

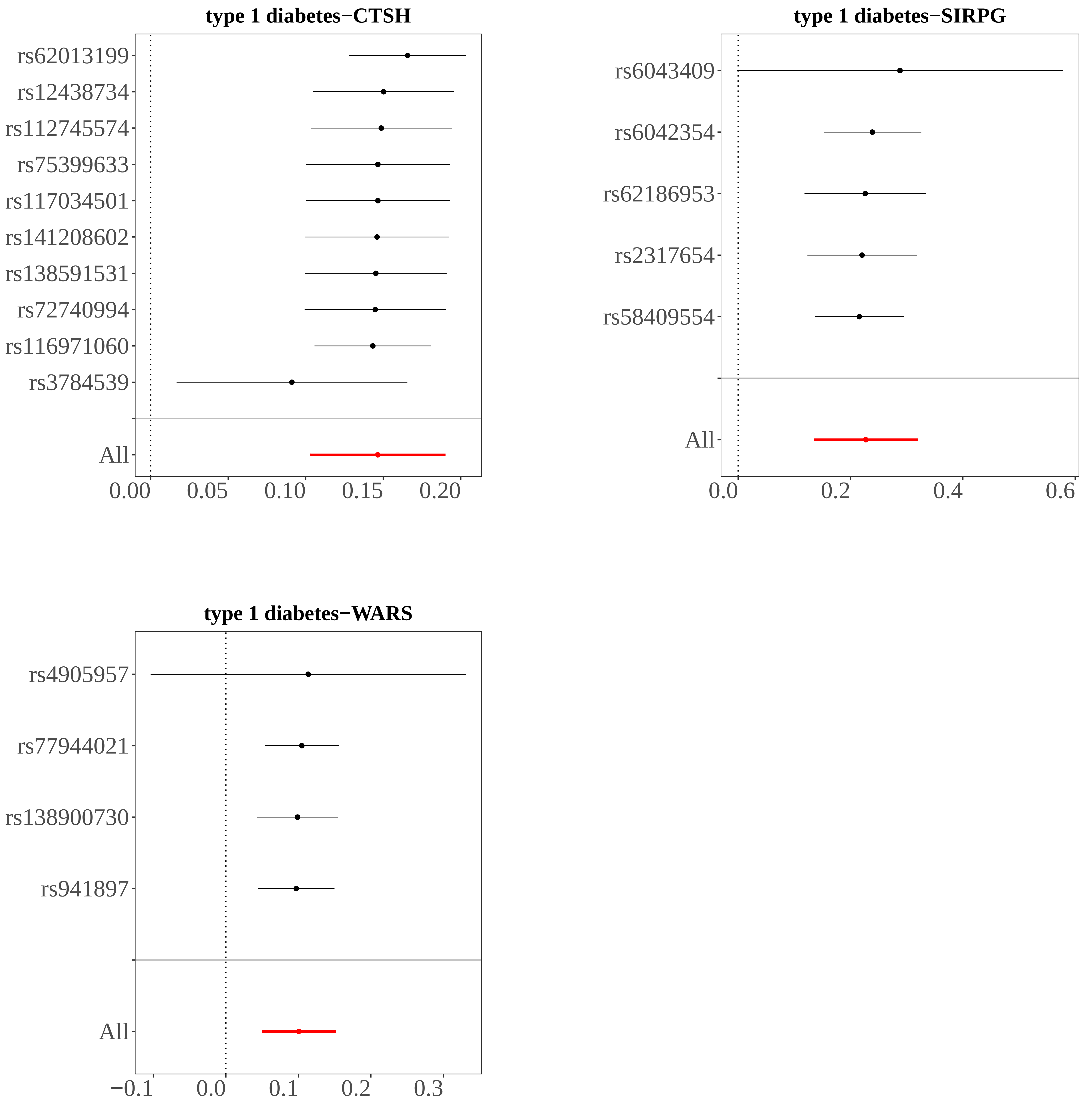
type 1 diabetes−CTSH
rs62013199
rs12438734
rs112745574
rs75399633
rs117034501
rs141208602
rs138591531
rs72740994
rs116971060
rs3784539
All
0.00
0.05
0.10
0.15
0.20
type 1 diabetes−SIRPG
rs6043409
rs6042354
rs62186953
rs2317654
rs58409554
All
0.0
0.2
0.4
0.6
type 1 diabetes−WARS
rs4905957
rs77944021
rs138900730
rs941897
All
−0.1
0.0
0.1
0.2
0.3
