# Supplementary material for: Protein-centric omics integration analysis identifies candidate plasma proteins for multiple autoimmune diseases
Source: Hum Genet. 2023 Dec 24;143(9-10):1035–48. doi: 10.1007/s00439-023-02627-0 (PMC11485194; doi:10.1007/s00439-023-02627-0)

Supplementary File 4 :

Protein-centric omics integration analysis identifies candidate plasma proteins for multiple autoimmune diseases

Yingxuan Chen[1,2†], Shuai Liu[1,2†], Weiming Gong[1,2], Ping Guo[1,2], Fuzhong Xue[1,2], Xiang Zhou[3,4], Shukang Wang[1,2*], Zhongshang Yuan[1,2*]

[1] Department of Biostatistics, School of Public Health, Cheeloo College of Medicine, Shandong University, 44, Wenhuaxi Road, Jinan, Shandong, 250012, China.

[2] Institute for Medical Dataology, Shandong University, 12550, Erhuan East Road, Jinan, Shandong, 250003, China.

[3] Department of Biostatistics, University of Michigan, Ann Arbor, MI 48109, USA.

[4] Center for Statistical Genetics, University of Michigan, Ann Arbor, MI 48109, USA.

[†] Y.C. and S.L. contributed equally to this work.

* Joint correspondence authors to:

Prof Shukang Wang

Department of Biostatistics, School of Public Health, Cheeloo College of Medicine, Shandong University, 44, Wenhua West Road, Jinan, Shandong, 250012, China.

Email: wsk2001@sdu.edu.cn

Prof Zhongshang Yuan

Department of Biostatistics, School of Public Health, Cheeloo College of Medicine, Shandong University, 44, Wenhua West Road, Jinan, Shandong, 250012, China.

Email: yuanzhongshang@sdu.edu.cn

Figure S3: LocusZoom plots of 21 protein-AID pairs identified from colocalization.

The index SNP, which is the SNP with the smallest p-value in the genomic risk loci, was indicated by a purple circle. The association of an individual variant is plotted as -log10 (P value) against chromosomal position. For each locus, from top to the bottom, the LocusZoom plot was derived from summary statistics of protein and AID, respectively, with the genes located in this region presented.

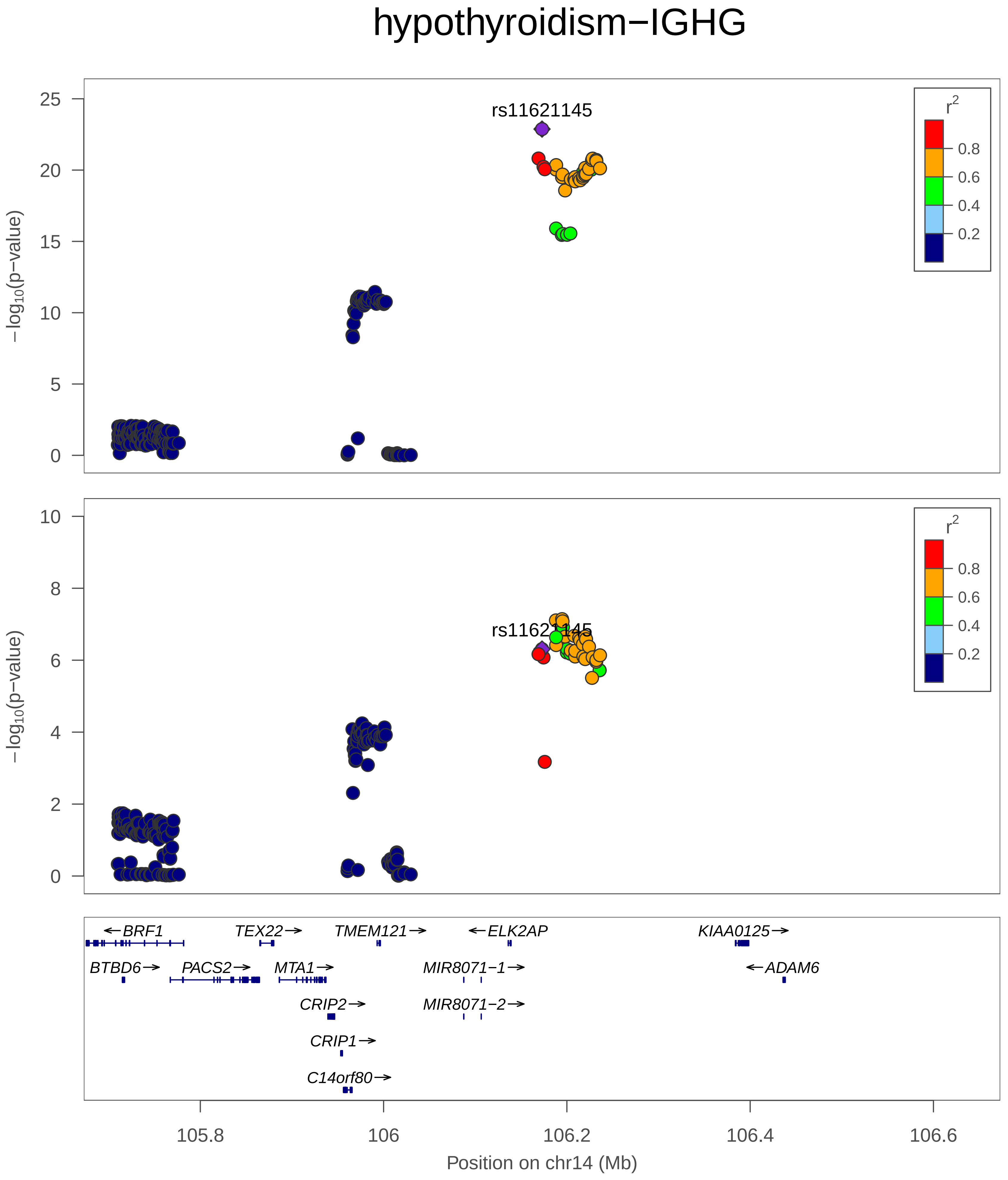
hypothyroidism−IGHG
rs11621145
r2
0.8
0.6
0.4
0.2
−log10(p−value)
25
20
15
10
5
0
rs11621145
10
8
6
4
2
0
BRF1
TEX22
TMEM121
ELK2AP
KIAA0125
BTBD6
PACS2
MTA1
MIR8071−1
ADAM6
CRIP2
MIR8071−2
CRIP1
C14orf80
105.8
106
106.2
106.4
106.6
Position on chr14 (Mb)

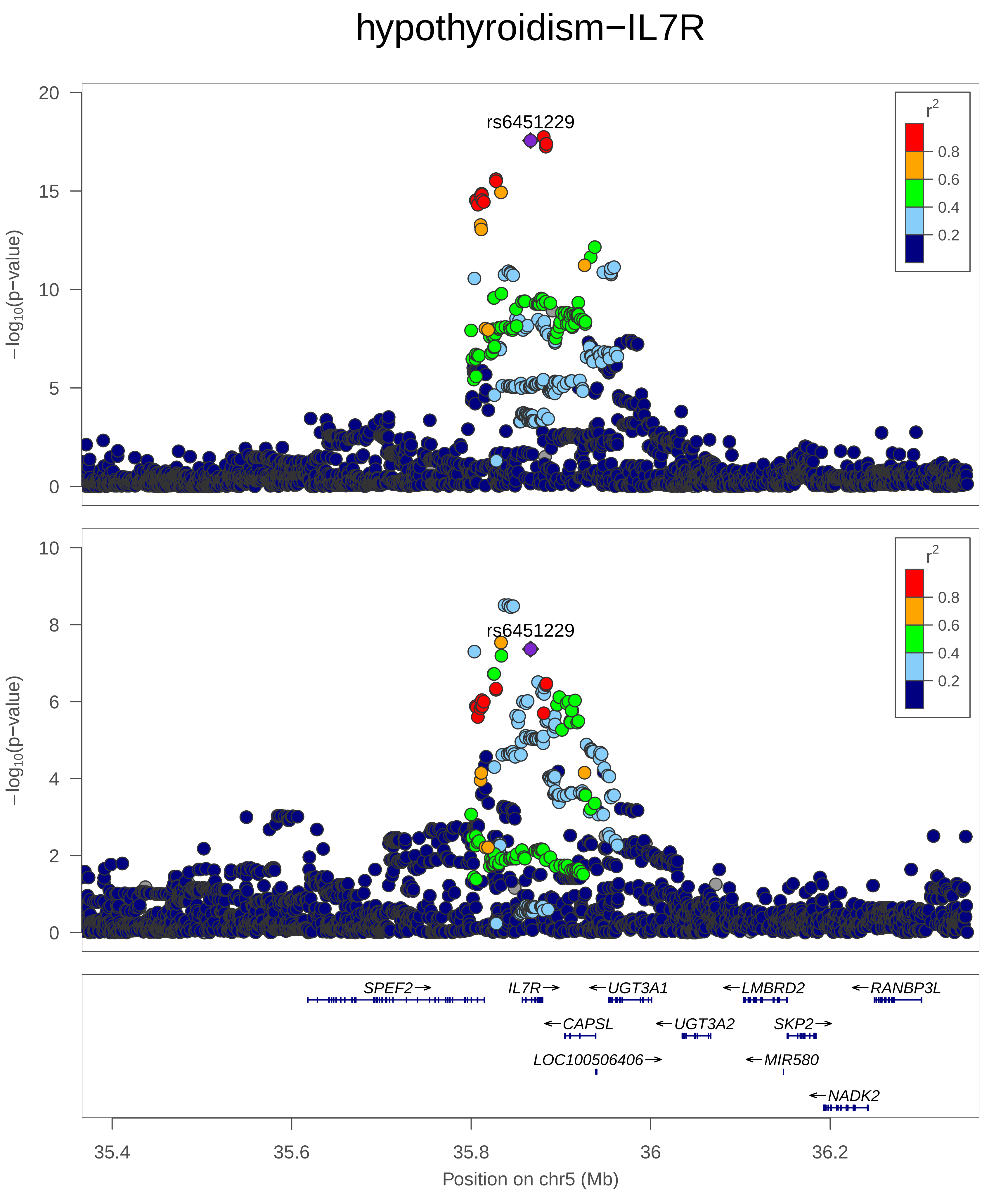
hypothyroidism−IL7R
rs6451229
r2
0.8
0.6
0.4
0.2
−log10(p−value)
20
15
10
5
0
rs6451229
r2
0.8
0.6
0.4
0.2
−log10(p−value)
10
8
6
4
2
0
SPEF2
IL7R
UGT3A1
LMBRD2
RANBP3L
CAPSL
UGT3A2
SKP2
LOC100506406
MIR580
NADK2
35.4
35.6
35.8
36
36.2
Position on chr5 (Mb)

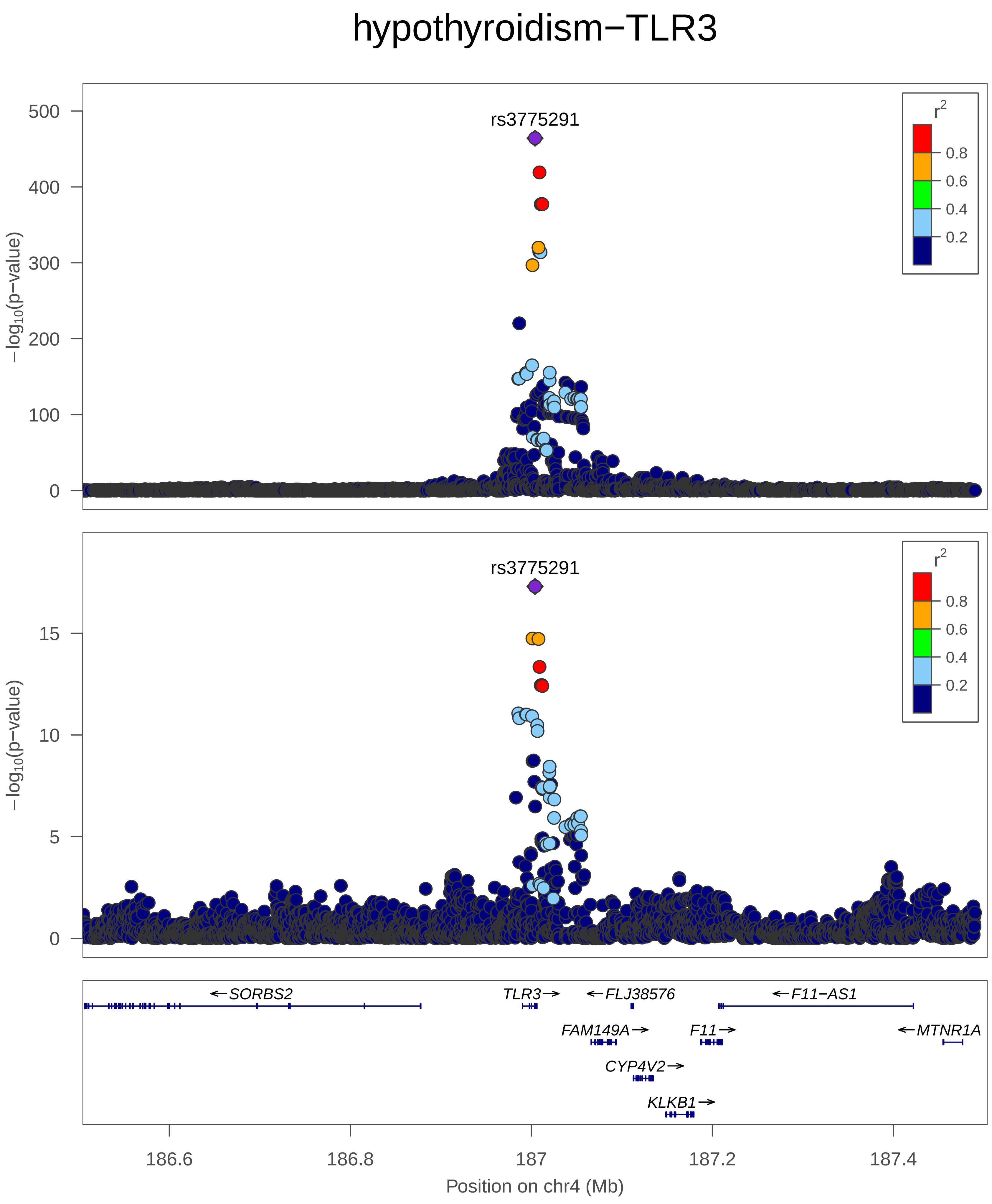

hypothyroidism−TLR3
rs3775291
−log10(p−value)
r2
0.8
0.6
0.4
0.2
SORBS2
TLR3
FLJ38576
F11−AS1
FAM149A
F11
MTNR1A
CYP4V2
KLKB1
186.6
186.8
187
187.2
187.4
Position on chr4 (Mb)

inflammatory bowel disease−FCGR2A

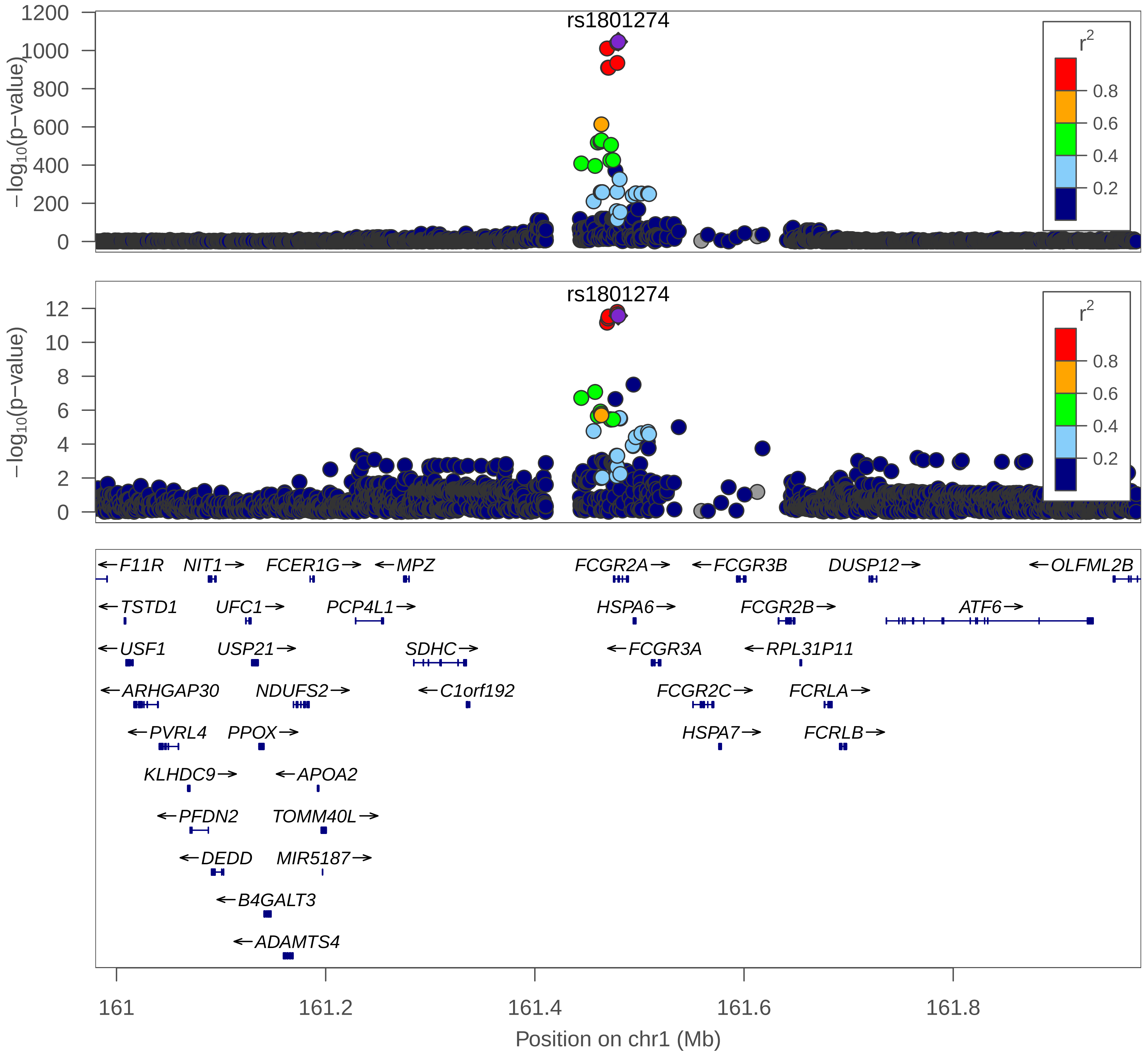

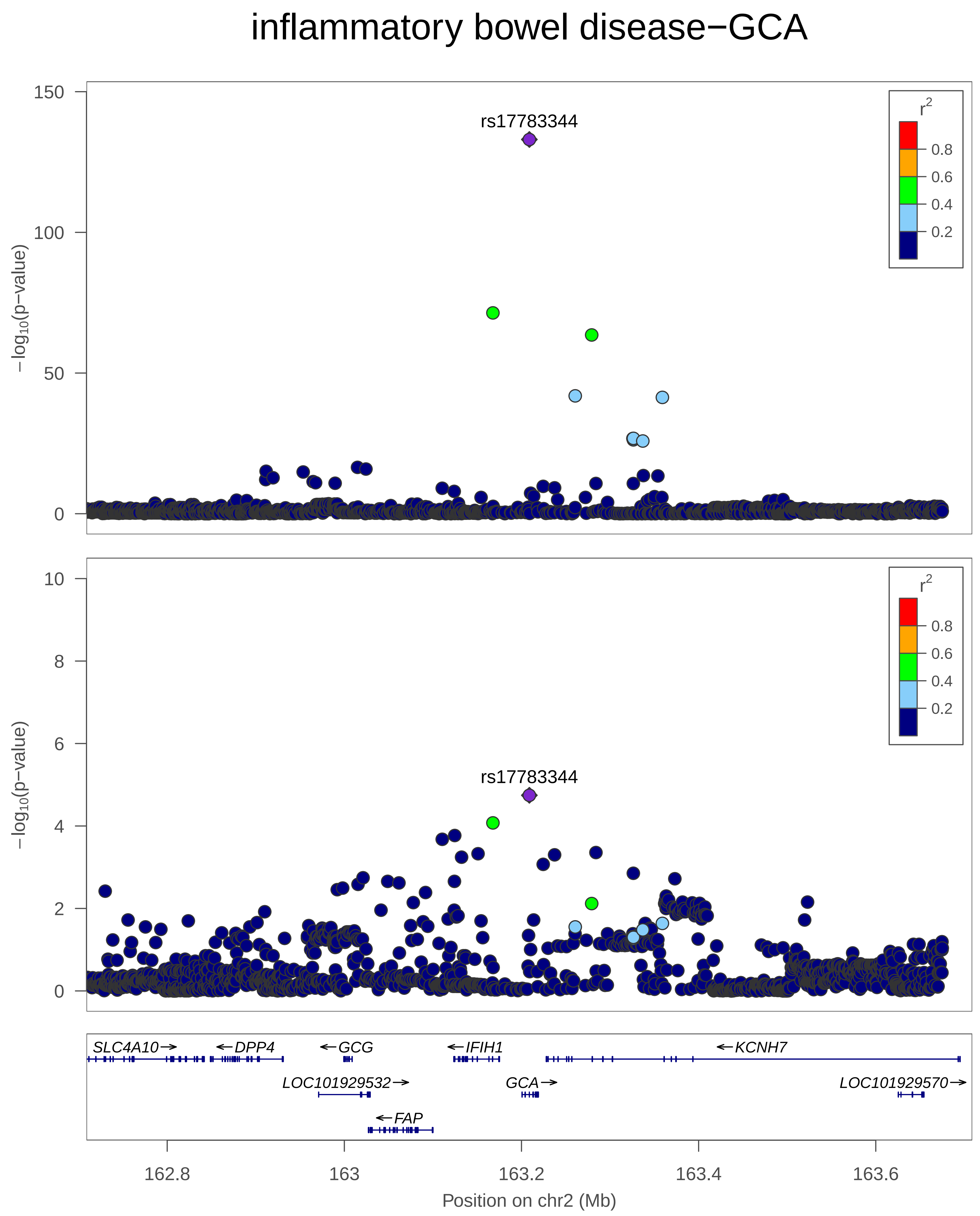

inflammatory bowel disease−GCA
150
100
50
0
−log10(p−value)
rs17783344
r2
0.8
0.6
0.4
0.2
10
8
6
4
2
0
−log10(p−value)
rs17783344
r2
0.8
0.6
0.4
0.2
SLC4A10→
←DPP4
←GCG
←IFIH1
←KCNH7
LOC101929532→
GCA→
LOC101929570→
←FAP
162.8
163
163.2
163.4
163.6
Position on chr2 (Mb)

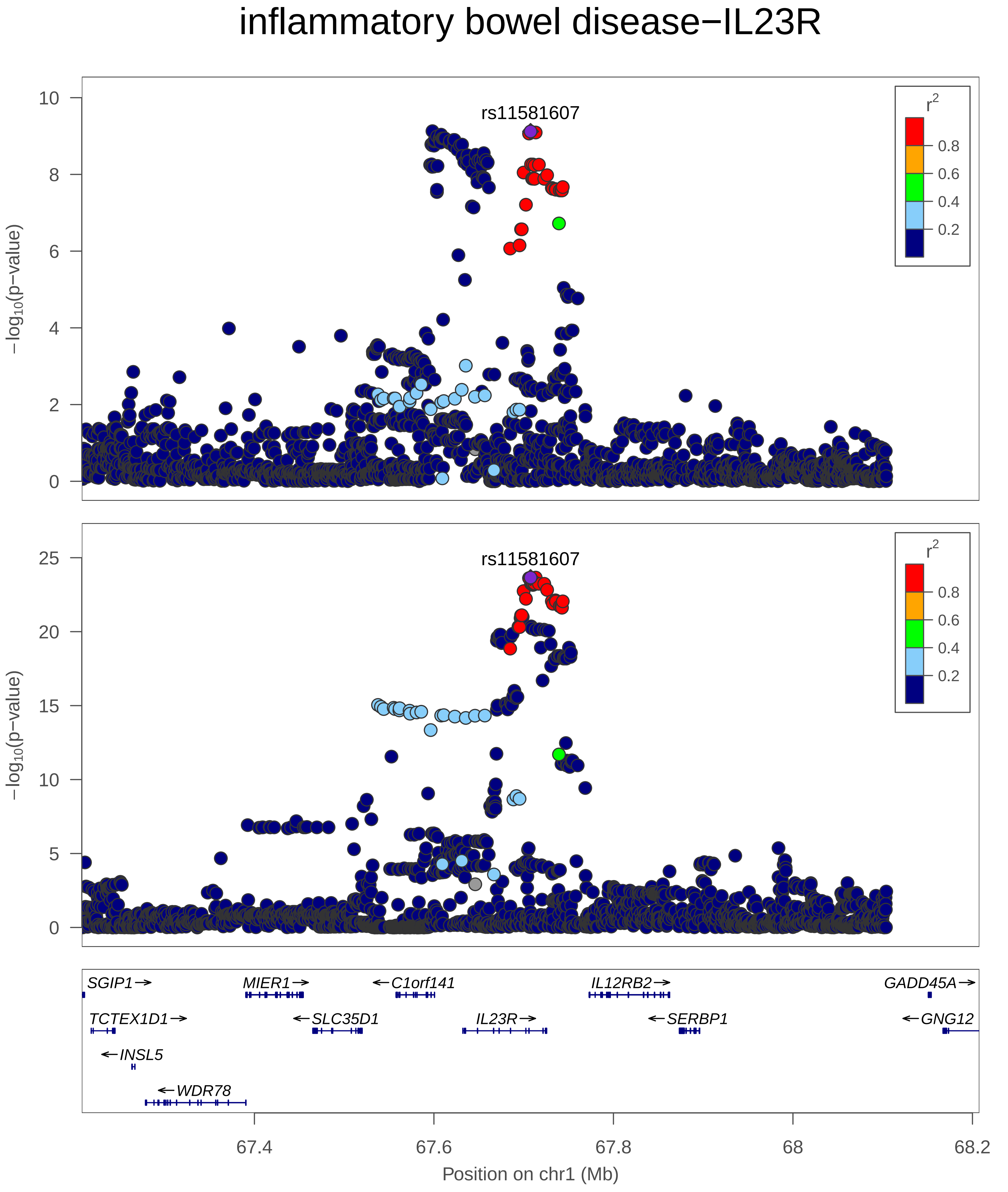

inflammatory bowel disease−IL23R
rs11581607
rs11581607
−log10(p−value)
−log10(p−value)
r2
0.8
0.6
0.4
0.2
10
8
6
4
2
0
25
20
15
10
5
0
SGIP1→
MIER1→
←C1orf141
IL12RB2→
GADD45A→
TCTEX1D1→
←SLC35D1
IL23R→
←SERBP1
←GNG12
←INSL5
←WDR78
67.4
67.6
67.8
68
68.2
Position on chr1 (Mb)

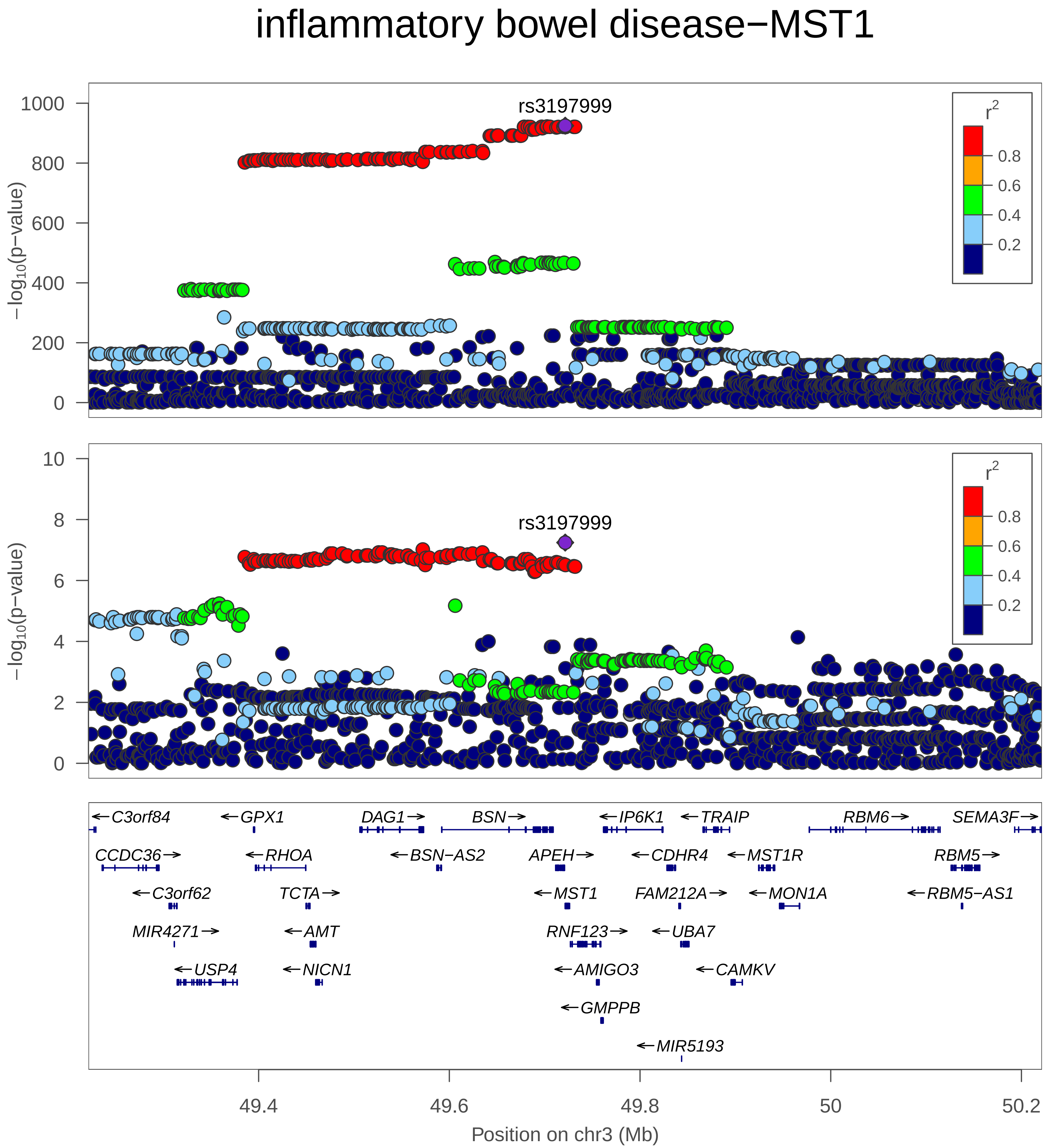

inflammatory bowel disease−MST1
rs3197999
−log10(p−value)
1000
800
600
400
200
0
r2
0.8
0.6
0.4
0.2
10
8
6
4
2
C3orf84
GPX1
DAG1
BSN
IP6K1
TRAIP
RBM6
SEMA3F
CCDC36
RHOA
BSN−AS2
APEH
CDHR4
MST1R
RBM5
C3orf62
TCTA
MST1
FAM212A
MON1A
RBM5−AS1
MIR4271
AMT
RNF123
UBA7
USP4
NICN1
AMIGO3
CAMKV
GMPPB
MIR5193
49.4
49.6
49.8
50
50.2
Position on chr3 (Mb)

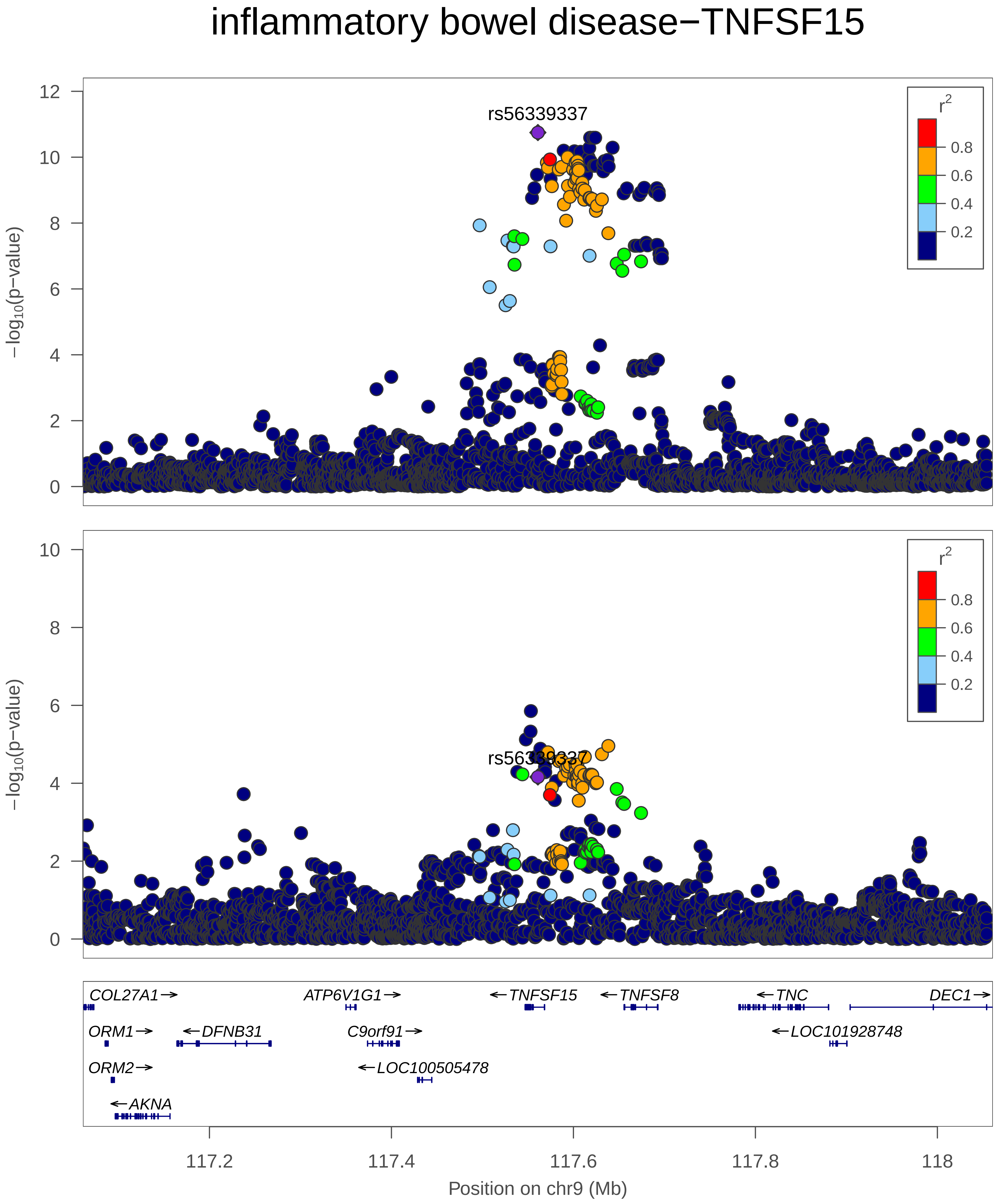
inflammatory bowel disease−TNFSF15
rs56339337
−log10(p−value)
r2
0.8
0.6
0.4
0.2
rs56339337
COL27A1→
ATP6V1G1→
←TNFSF15
←TNFSF8
←TNC
DEC1→
ORM1→
←DFNB31
C9orf91→
←LOC101928748
ORM2→
←LOC100505478
←AKNA
117.2
117.4
117.6
117.8
118
Position on chr9 (Mb)

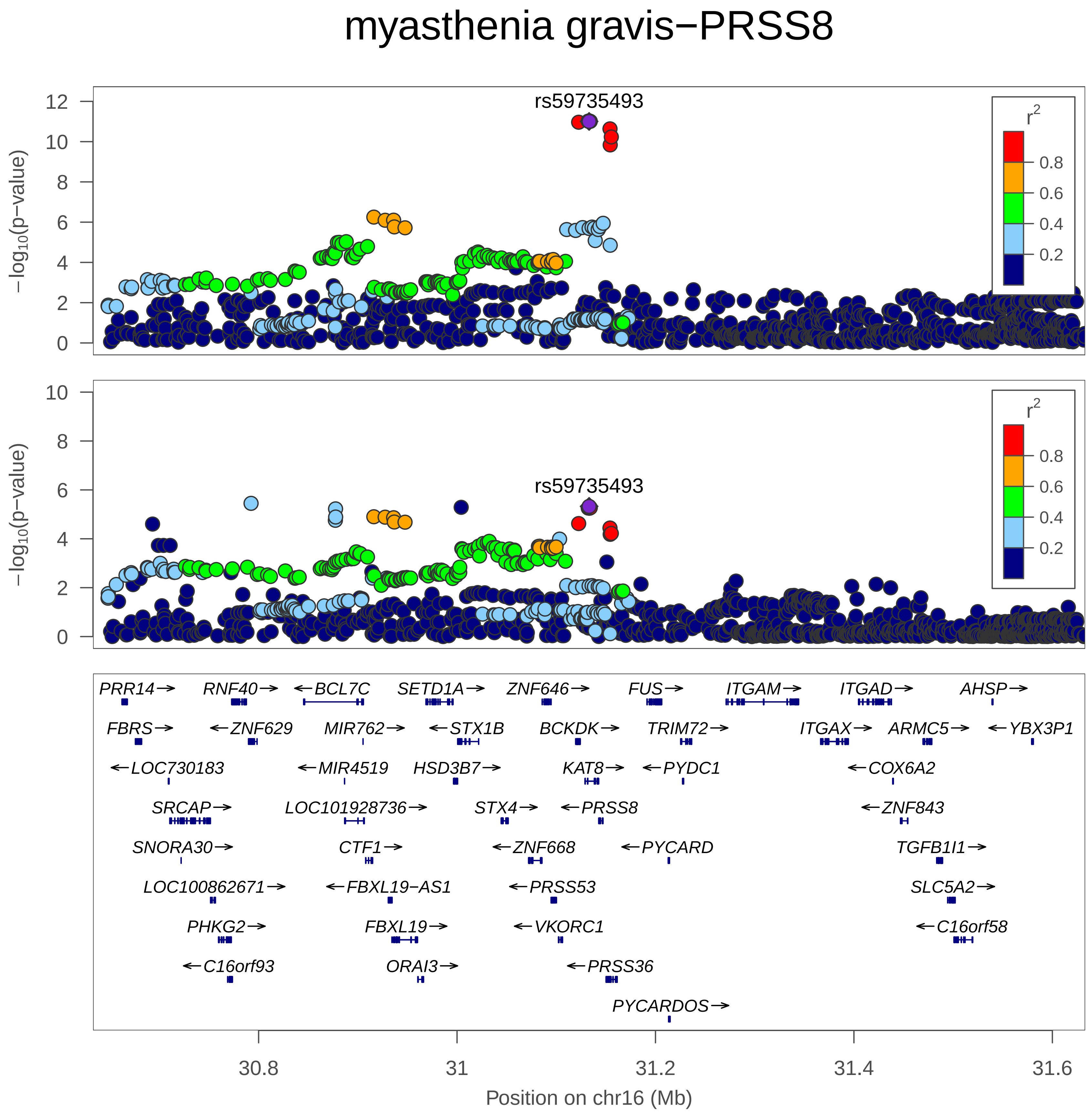

myasthenia gravis−PRSS8
rs59735493
−log10(p−value)
r2
0.8
0.6
0.4
0.2
PRR14→
RNF40→
←BCL7C
SETD1A→
ZNF646→
FUS→
ITGAM→
ITGAD→
AHSP→
FBRS→
←ZNF629
MIR762→
←STX1B
BCKDK→
TRIM72→
ITGAX→
ARMC5→
←YBX3P1
←LOC730183
←MIR4519
HSD3B7→
KAT8→
←PYDC1
←COX6A2
SRCAP→
LOC101928736→
STX4→
←PRSS8
←ZNF843
SNORA30→
CTF1→
←ZNF668
←PYCARD
TGFB1I1→
LOC100862671→
←FBXL19−AS1
←PRSS53
SLC5A2→
PHKG2→
FBXL19→
←VKORC1
←C16orf58
←C16orf93
ORAI3→
←PRSS36
PYCARDOS→
30.8
31
31.2
31.4
31.6
Position on chr16 (Mb)

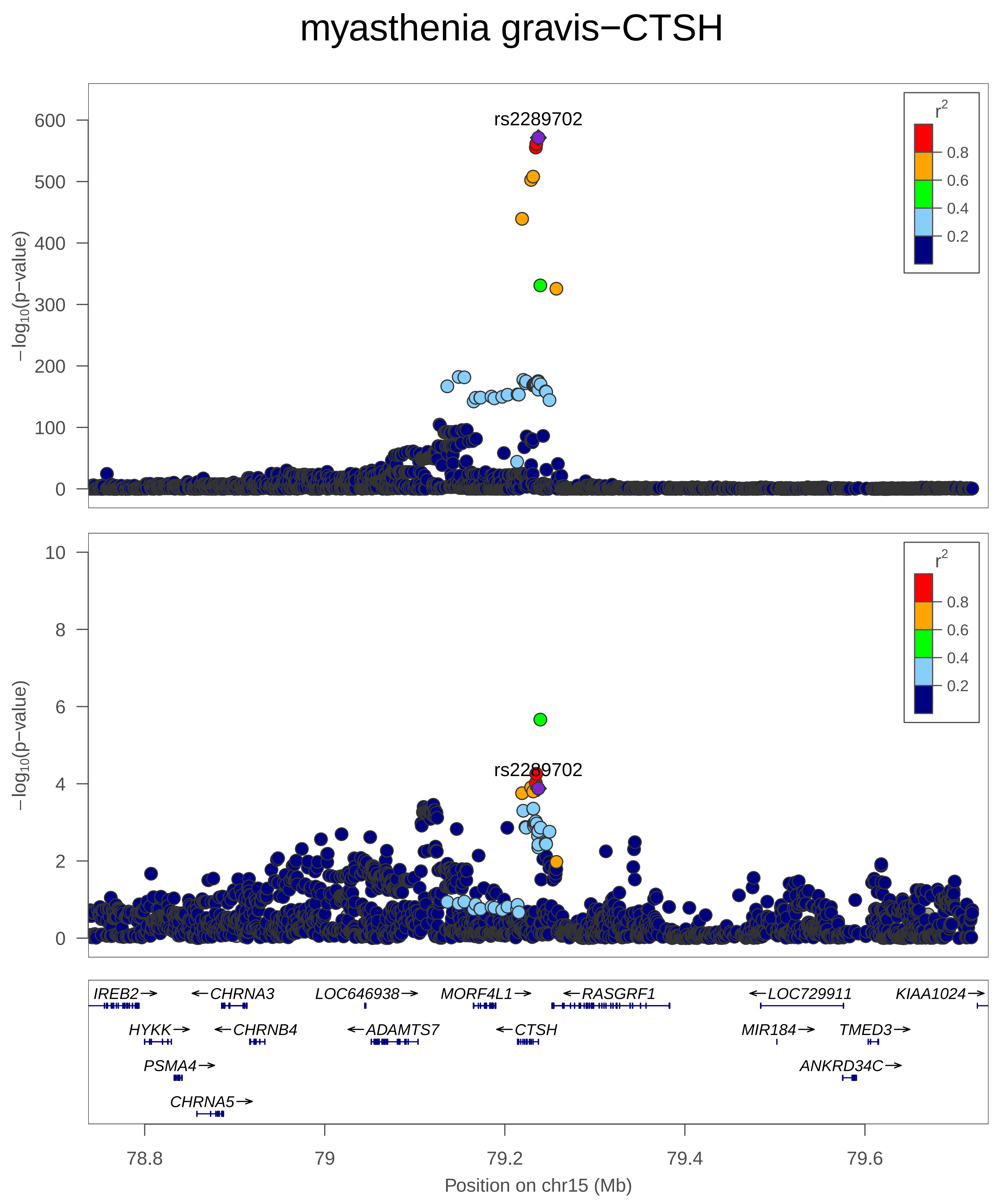
myasthenia gravis−CTSH
rs2289702
r2
0.8
0.6
0.4
0.2
600
500
400
300
200
100
0
−log10(p−value)
10
8
6
4
2
IREB2→
←CHRNA3
LOC646938→
MORF4L1→
←RASGRF1
←LOC729911
KIAA1024→
HYKK→
←CHRNB4
←ADAMTS7
←CTSH
MIR184→
TMED3→
PSMA4→
ANKRD34C→
CHRNA5→
78.8
79
79.2
79.4
79.6
Position on chr15 (Mb)

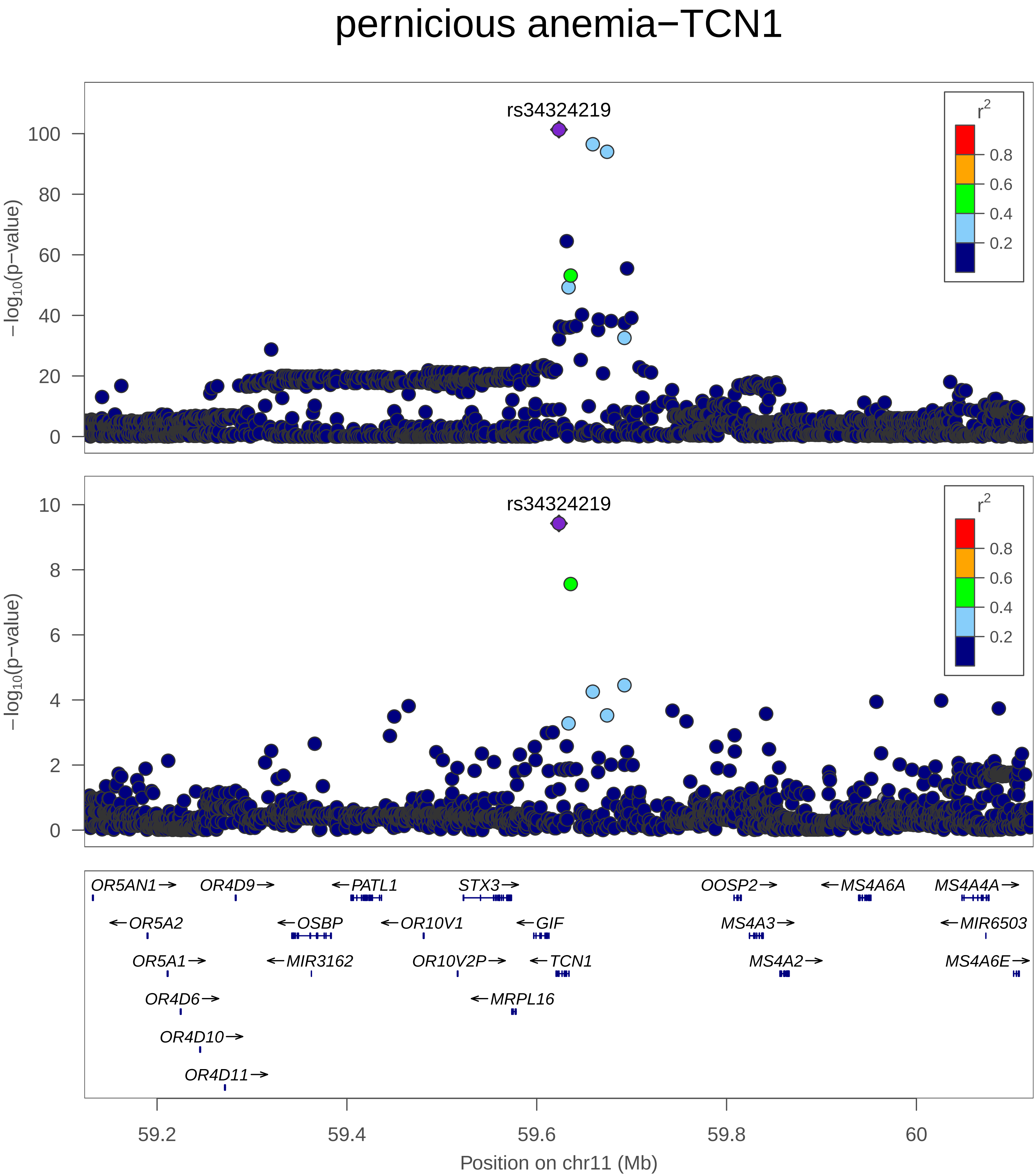

pernicious anemia−TCN1
rs34324219
r2
0.8
0.6
0.4
0.2
−log10(p−value)
100
80
60
40
20
0
rs34324219
10
8
6
4
2
0
OR5AN1→
OR4D9→
←PATL1
STX3→
OOSP2→
←MS4A6A
MS4A4A→
←OR5A2
←OSBP
←OR10V1
←GIF
MS4A3→
←MIR6503
OR5A1→
←MIR3162
OR10V2P→
←TCN1
MS4A2→
MS4A6E→
OR4D6→
←MRPL16
OR4D10→
OR4D11→
59.2
59.4
59.6
59.8
60
Position on chr11 (Mb)

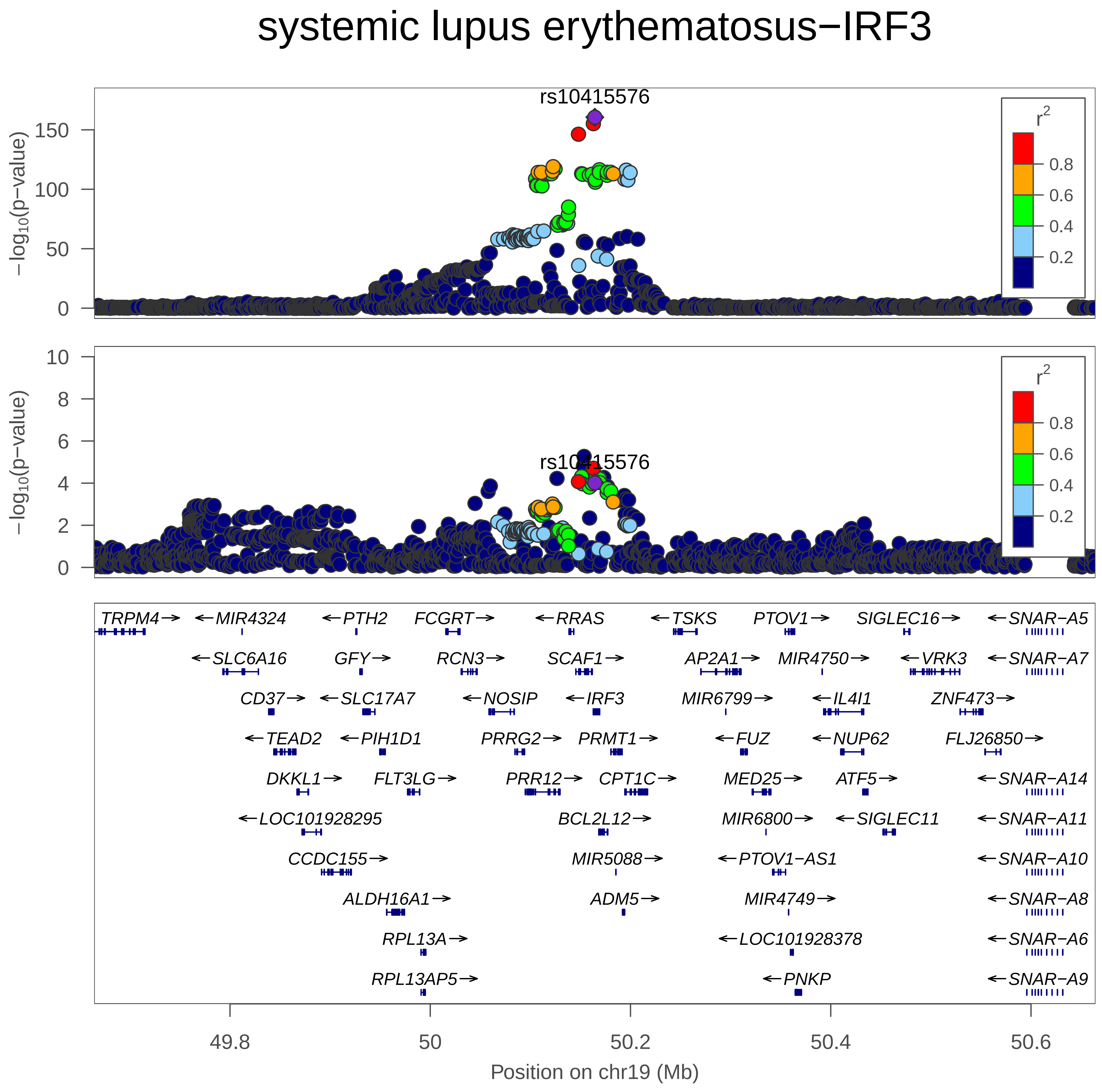
systemic lupus erythematosus−IRF3
rs10415576
−log10(p−value)
150
100
50
0
r2
0.8
0.6
0.4
0.2
rs10415576
−log10(p−value)
10
8
6
4
2
0
r2
0.8
0.6
0.4
0.2
TRPM4→
←MIR4324
←PTH2
FCGRT→
←RRAS
←TSKS
PTOV1→
SIGLEC16→
←SNAR−A5
←SLC6A16
GFY→
RCN3→
SCAF1→
AP2A1→
MIR4750→
←VRK3
←SNAR−A7
CD37→
←SLC17A7
←NOSIP
←IRF3
MIR6799→
←IL4I1
ZNF473→
←TEAD2
←PIH1D1
PRRG2→
PRMT1→
←FUZ
←NUP62
FLJ26850→
DKKL1→
FLT3LG→
PRR12→
CPT1C→
MED25→
ATF5→
←SNAR−A14
←LOC101928295
BCL2L12→
MIR6800→
←SIGLEC11
←SNAR−A11
CCDC155→
MIR5088→
←PTOV1−AS1
←SNAR−A10
ALDH16A1→
ADM5→
MIR4749→
←SNAR−A8
RPL13A→
←LOC101928378
←SNAR−A6
RPL13AP5→
←PNKP
←SNAR−A9
49.8
50
50.2
50.4
50.6
Position on chr19 (Mb)

systemic lupus erythematosus−NPPB

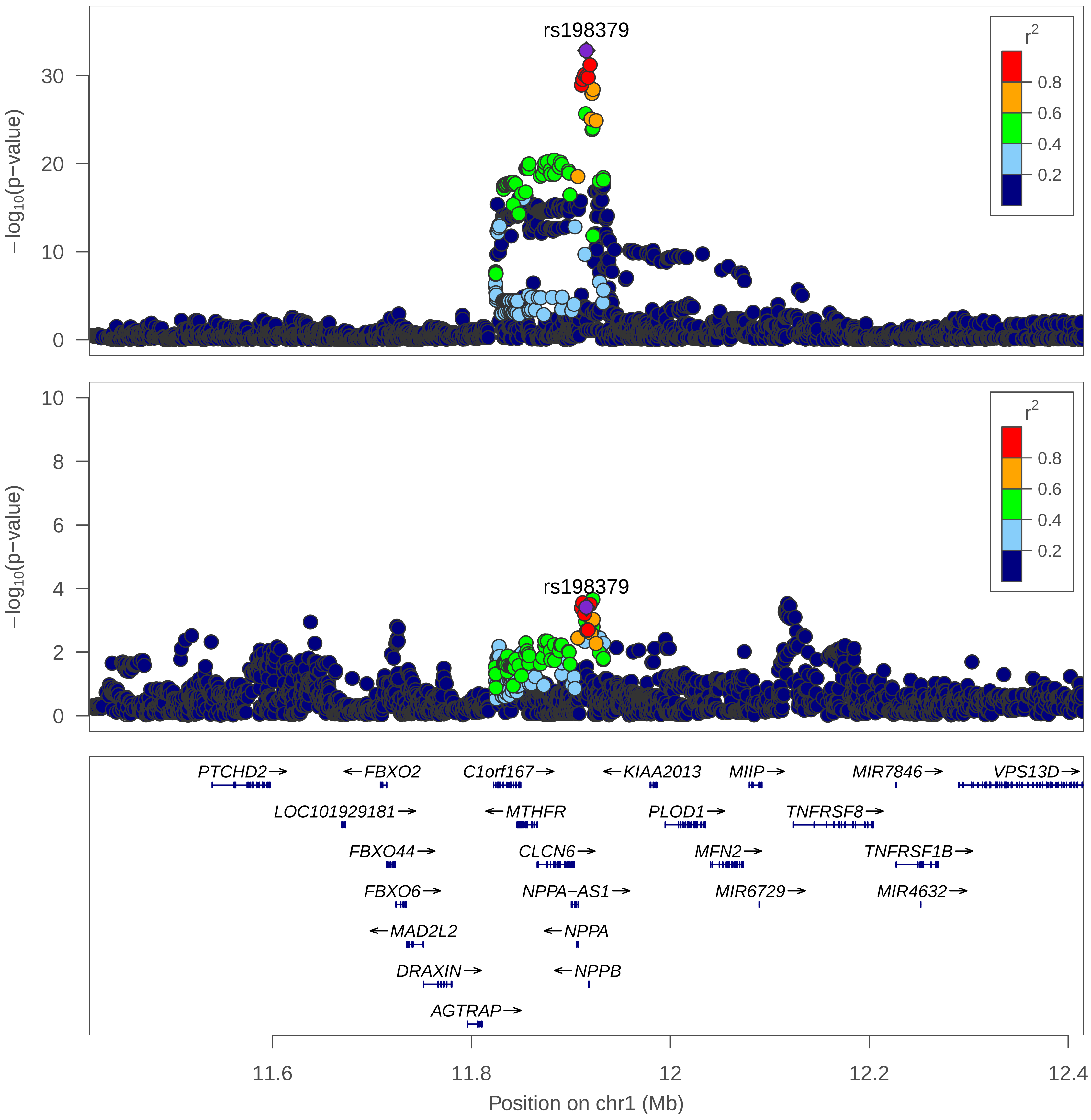

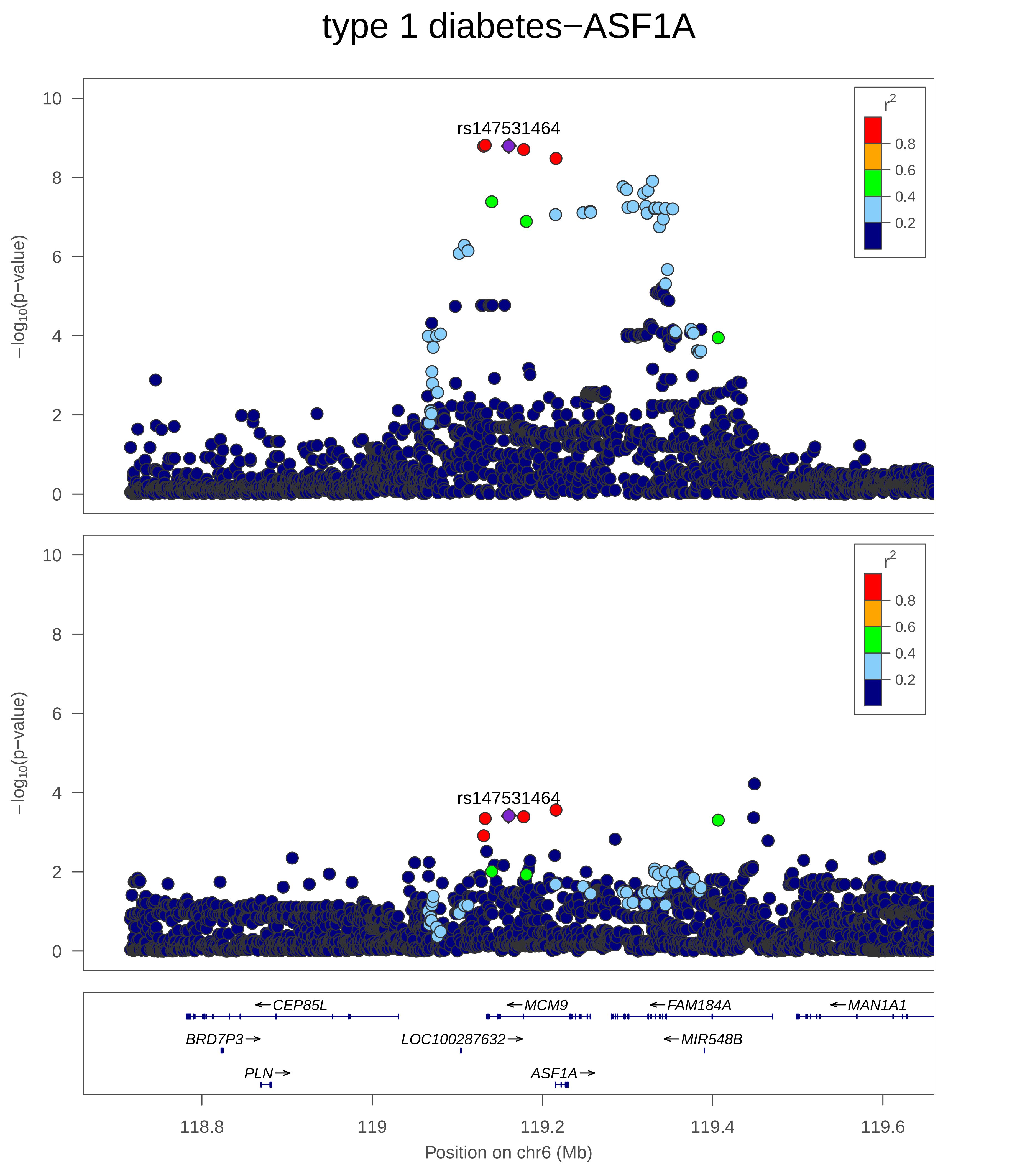
type 1 diabetes−ASF1A
rs147531464
r2
0.8
0.6
0.4
0.2
−log10(p−value)
10
8
6
4
2
0
rs147531464
CEP85L
MCM9
FAM184A
MAN1A1
BRD7P3
LOC100287632
MIR548B
PLN
ASF1A
118.8
119
119.2
119.4
119.6
Position on chr6 (Mb)

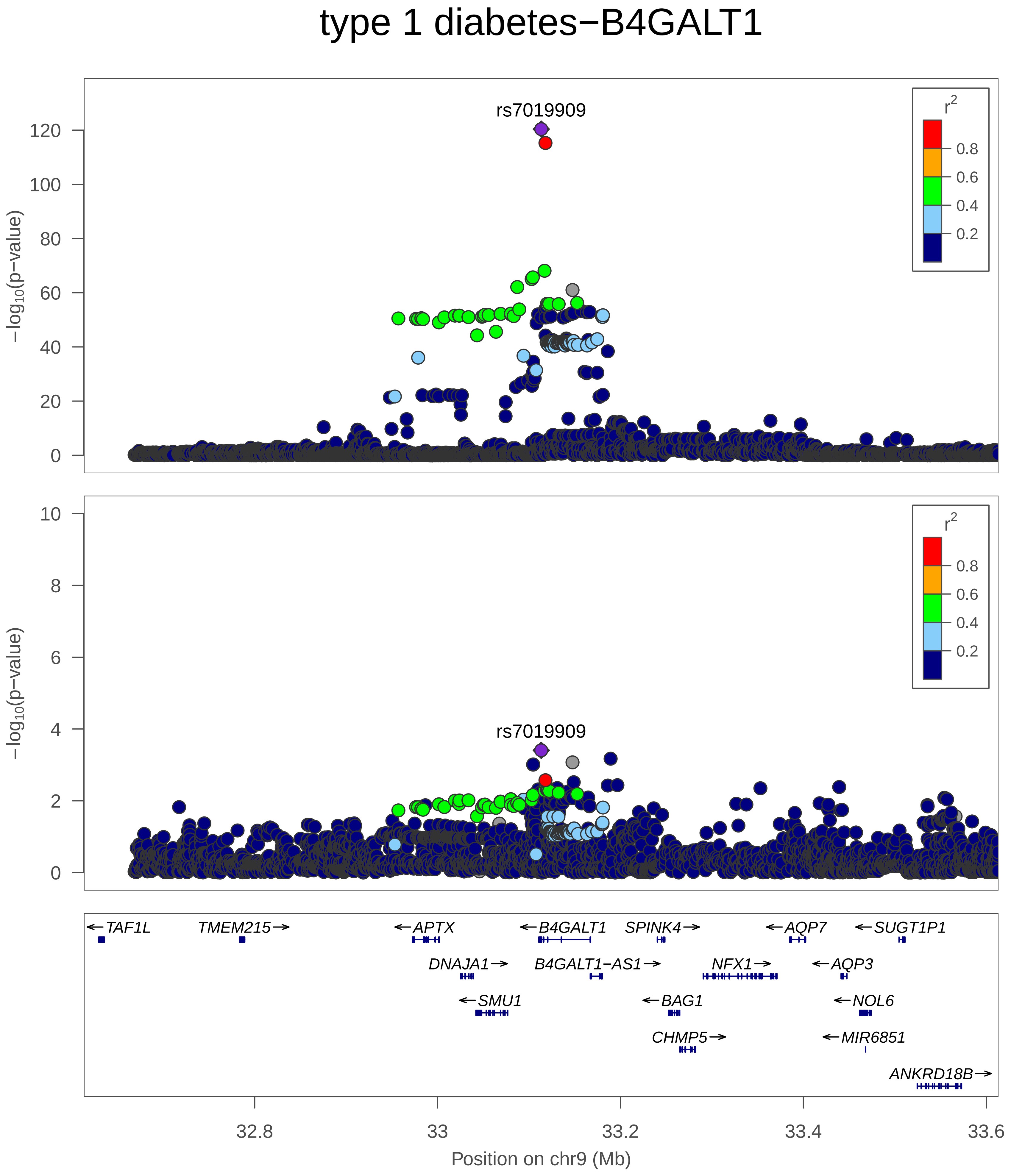

type 1 diabetes−B4GALT1
rs7019909
120
100
80
60
40
20
0
−log10(p−value)
r2
0.8
0.6
0.4
0.2
10
8
6
4
2
0
−log10(p−value)
rs7019909
r2
0.8
0.6
0.4
0.2
←TAF1L
TMEM215→
←APTX
←B4GALT1
SPINK4→
←AQP7
←SUGT1P1
DNAJA1→
B4GALT1−AS1→
NFX1→
←AQP3
←SMU1
←BAG1
←NOL6
CHMP5→
←MIR6851
ANKRD18B→
32.8
33
33.2
33.4
33.6
Position on chr9 (Mb)

type 1 diabetes−CCL25

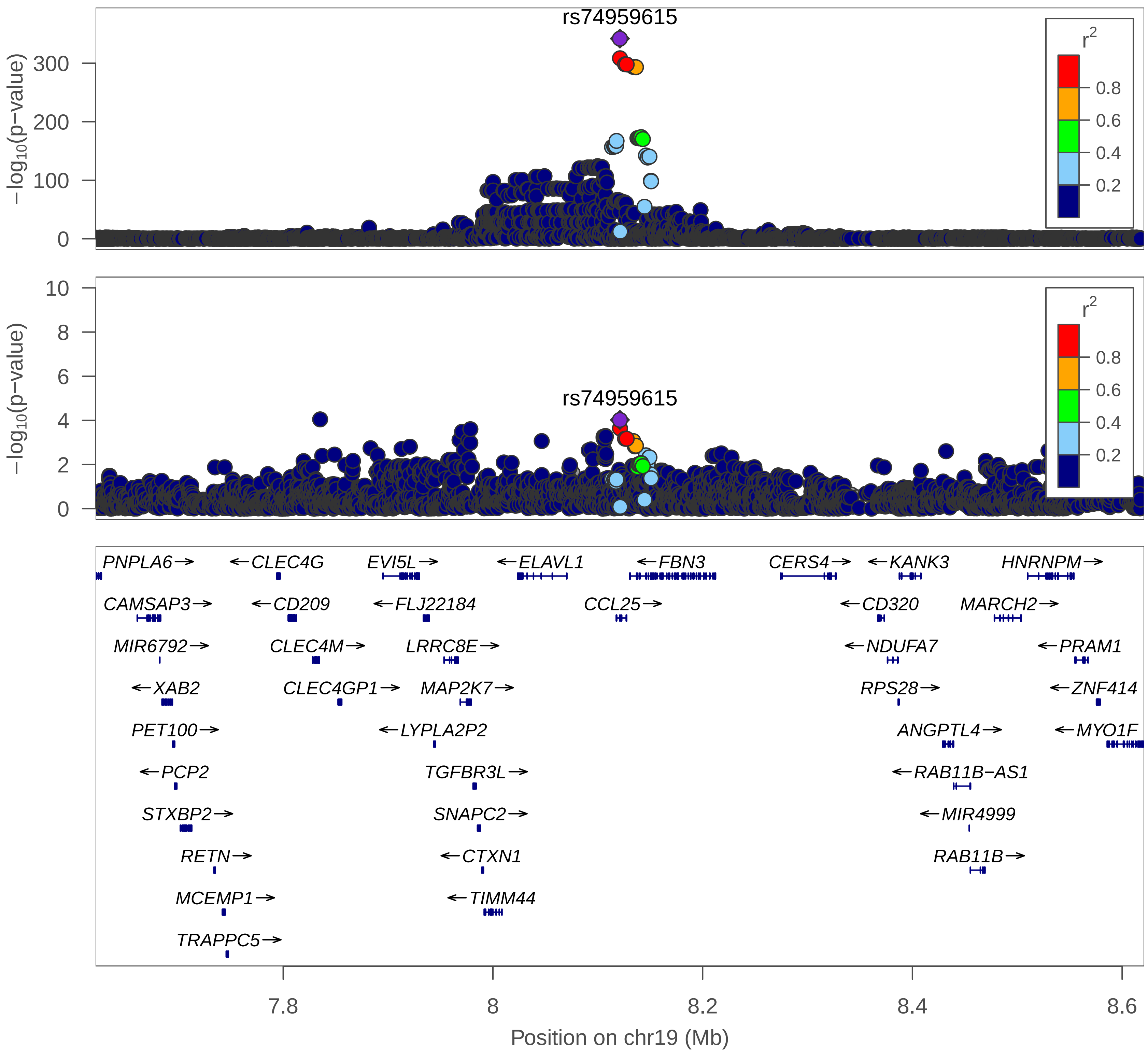

type 1 diabetes-IL27RA

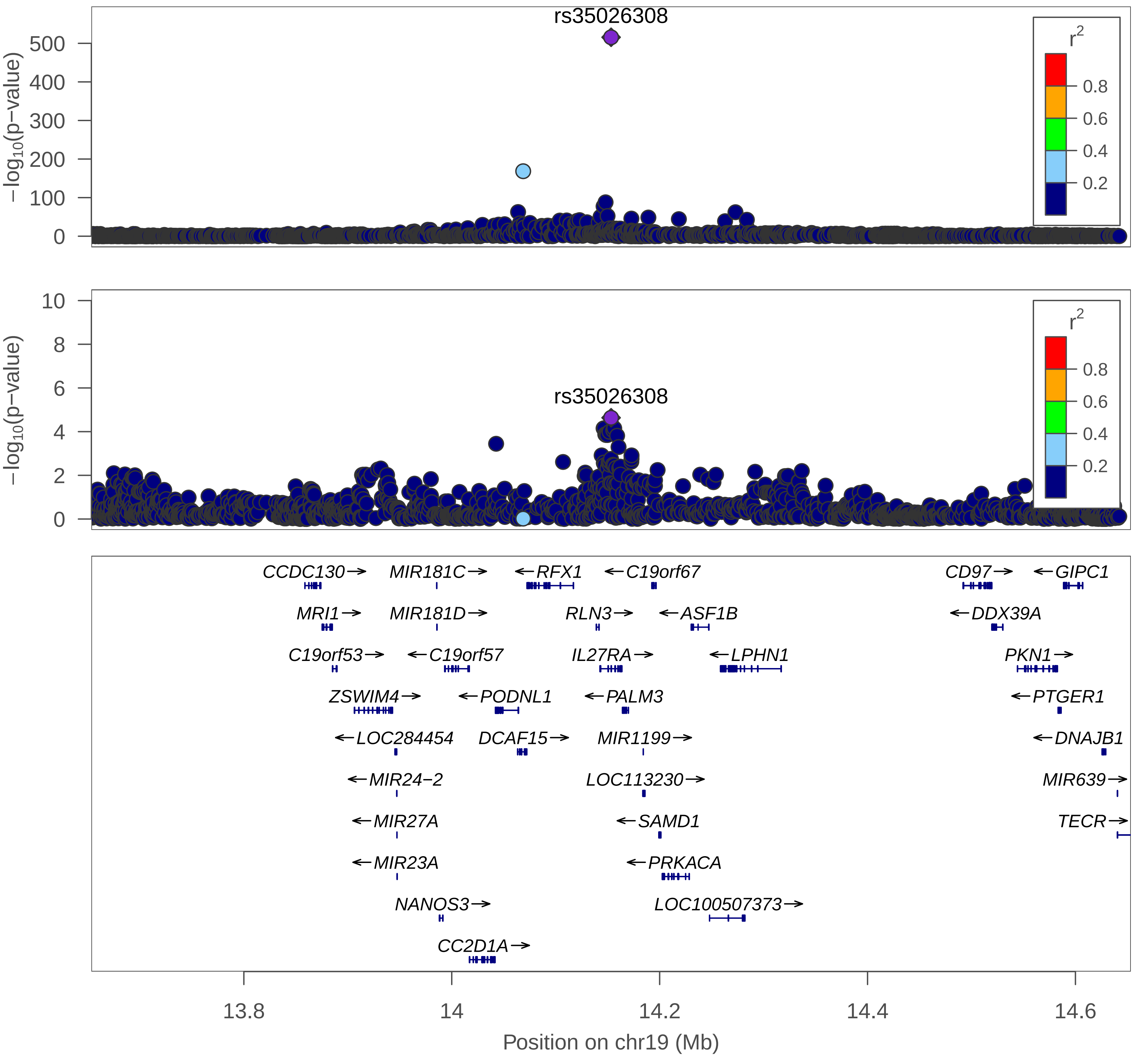

type 1 diabetes−CTSH

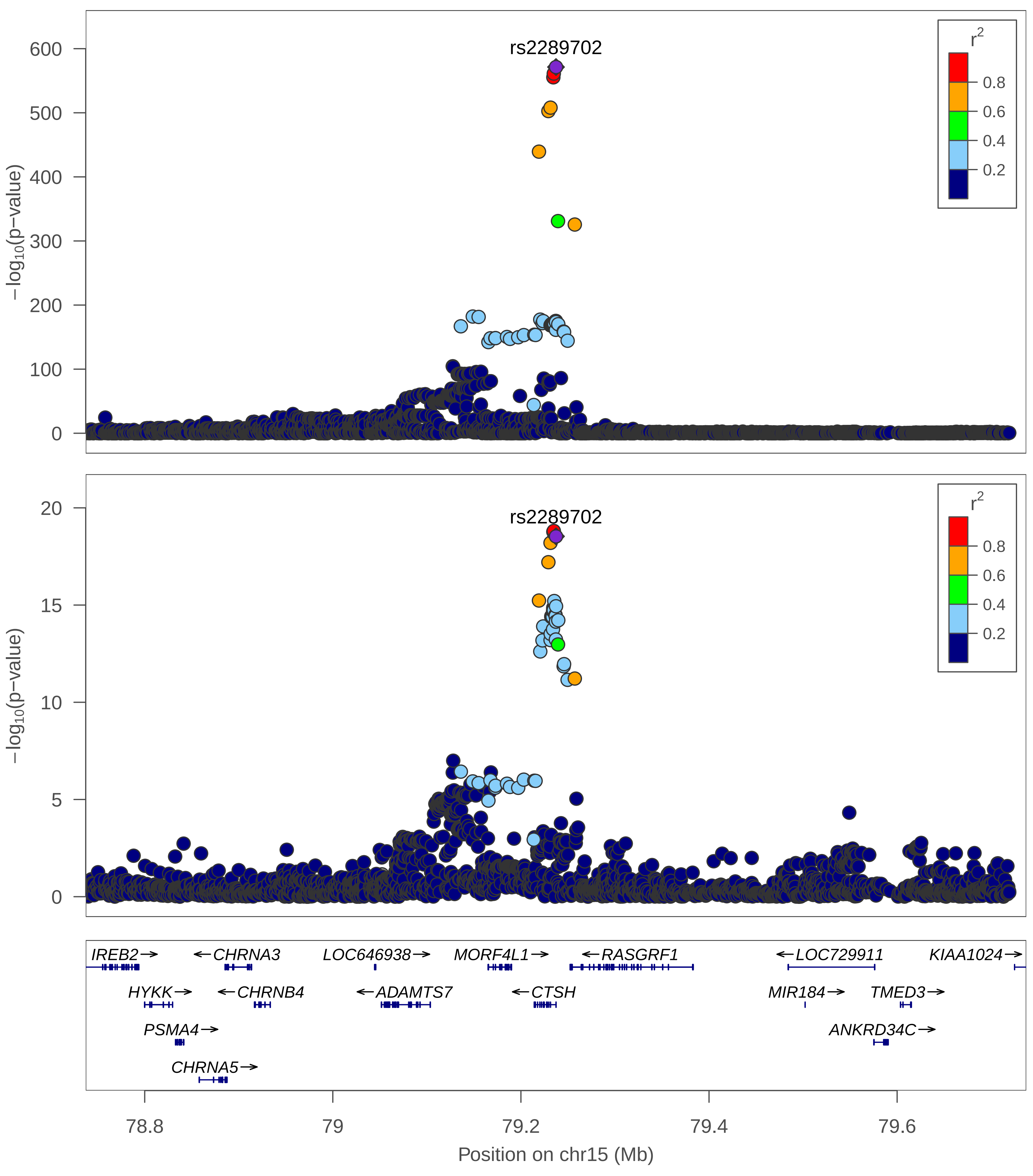

type 1 diabetes−INHBC

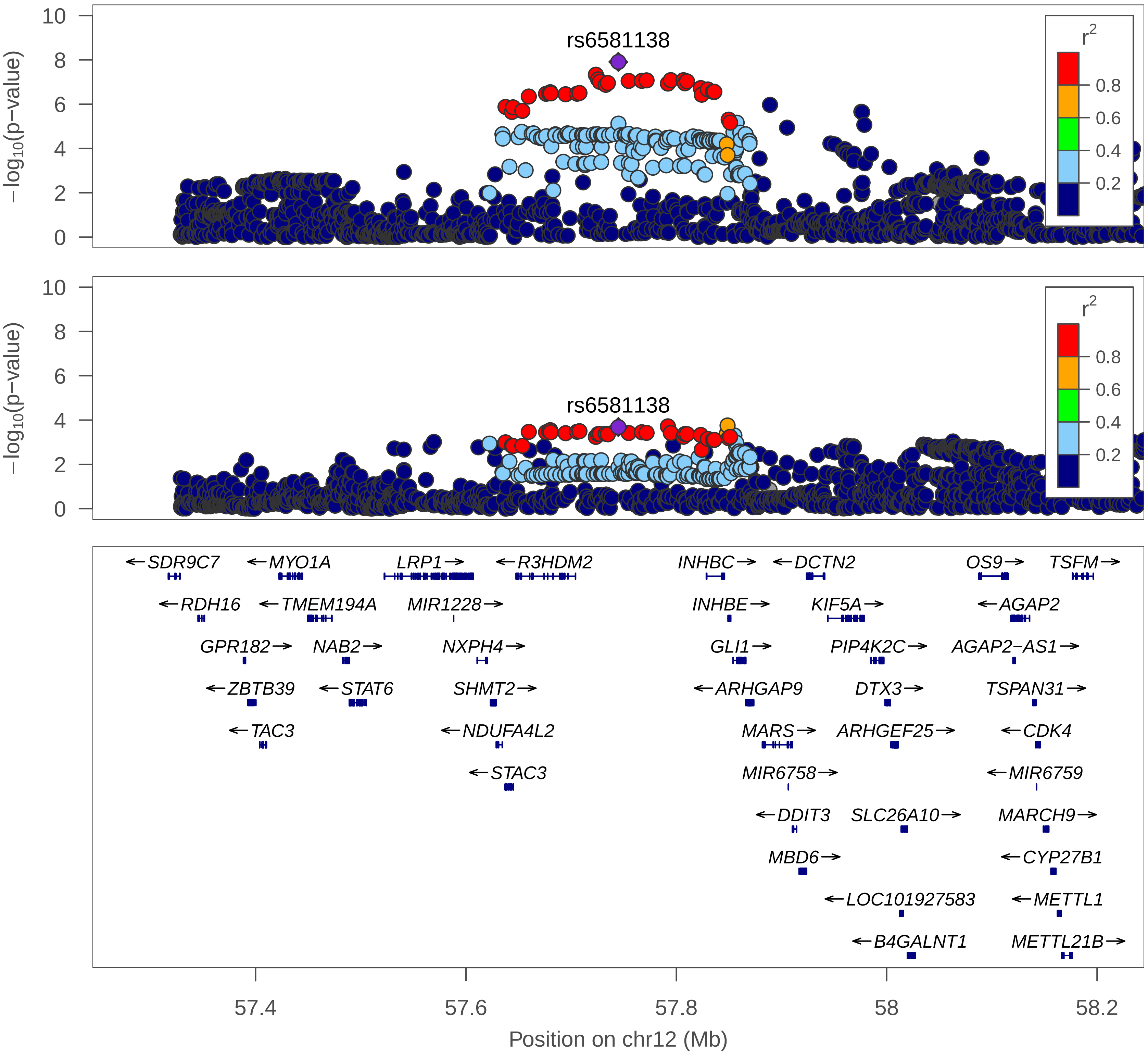

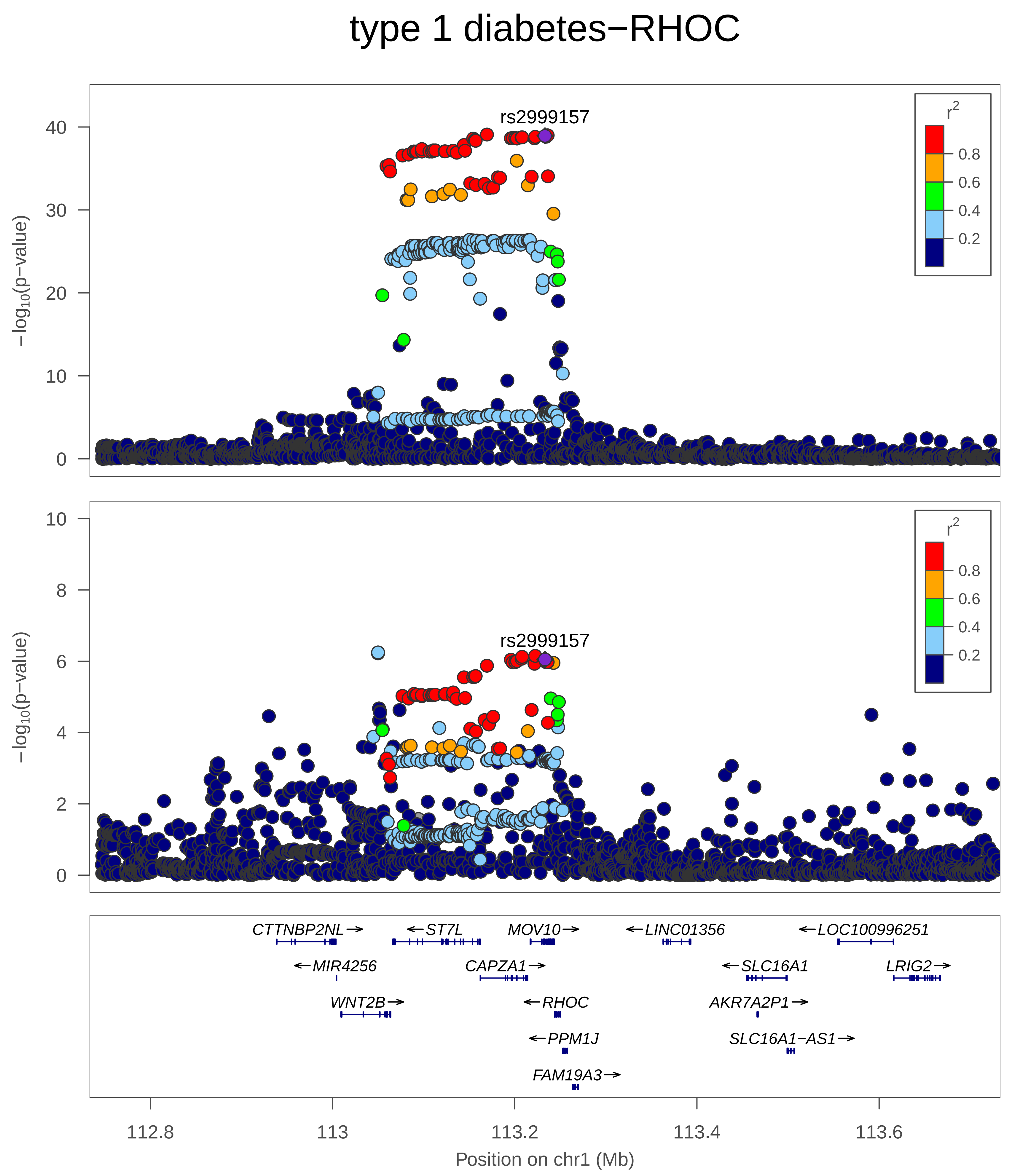
type 1 diabetes−RHOC
rs2999157
−log10(p−value)
r2
0.8
0.6
0.4
0.2
0
10
20
30
40
2
4
6
8
CTTNBP2NL
ST7L
MOV10
LINC01356
LOC100996251
MIR4256
CAPZA1
SLC16A1
LRIG2
WNT2B
RHOC
AKR7A2P1
PPM1J
SLC16A1−AS1
FAM19A3
112.8
113
113.2
113.4
113.6
Position on chr1 (Mb)

type 1 diabetes−SIRPG

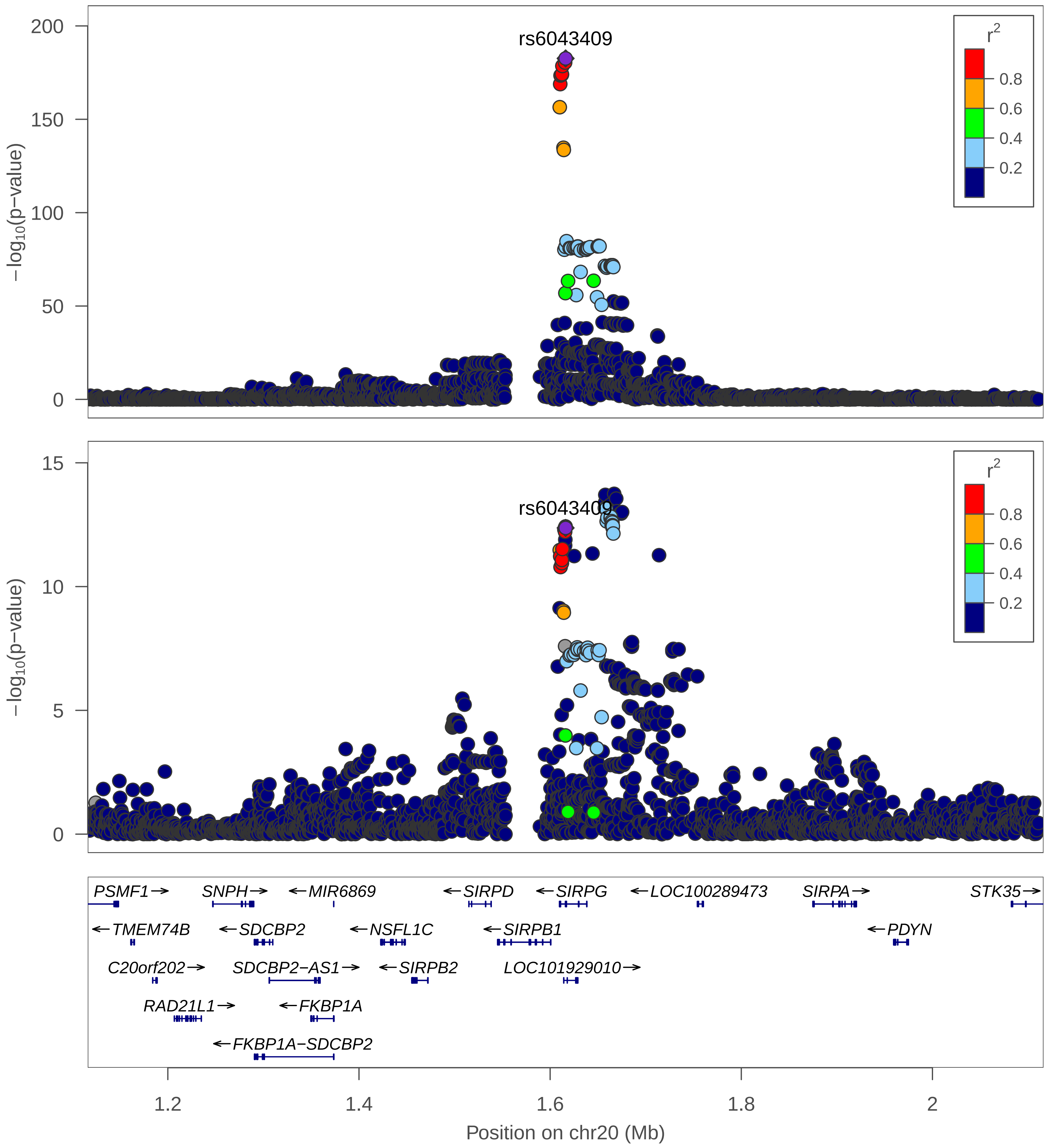

Supplement: Supplementary file 4 — Supplementary file4 (PDF 1170 KB)—Fig S3. LocusZoom plots of 21 protein-AID pairs identified from colocalization. [file 439_2023_2627_MOESM4_ESM.pdf]
